# A Phase I Double Blind, Placebo-Controlled, Randomized Study of a Multigenic HIV-1 Adenovirus Subtype 35 Vector Vaccine in Healthy Uninfected Adults

**DOI:** 10.1371/journal.pone.0041936

**Published:** 2012-08-03

**Authors:** Michael C. Keefer, Jill Gilmour, Peter Hayes, Dilbinder Gill, Jakub Kopycinski, Hannah Cheeseman, Michelle Cashin-Cox, Marloes Naarding, Lorna Clark, Natalia Fernandez, Catherine A. Bunce, Christine M. Hay, Sabrina Welsh, Wendy Komaroff, Lottie Hachaambwa, Tony Tarragona-Fiol, Eddy Sayeed, Devika Zachariah, James Ackland, Kelley Loughran, Burc Barin, Emmanuel Cormier, Josephine H. Cox, Patricia Fast, Jean-Louis Excler

**Affiliations:** 1 University of Rochester School of Medicine and Dentistry, Rochester, New York, United States of America; 2 IAVI Human Immunology Laboratory, Imperial College, London, United Kingdom; 3 International AIDS Vaccine Initiative (IAVI), New York, New York, United States of America; 4 Global BioSolutions, Melbourne, Victoria, Australia; 5 The EMMES Corporation, Rockville, Maryland, United States of America; University of Pittsburgh, United States of America

## Abstract

**Background:**

We conducted a phase I, randomized, double-blind, placebo-controlled trial to assess the safety and immunogenicity of escalating doses of two recombinant replication defective adenovirus serotype 35 (Ad35) vectors containing gag, reverse transcriptase, integrase and nef (Ad35-GRIN) and env (Ad35-ENV), both derived from HIV-1 subtype A isolates. The trial enrolled 56 healthy HIV-uninfected adults.

**Methods:**

Ad35-GRIN/ENV (Ad35-GRIN and Ad35-ENV mixed in the same vial in equal proportions) or Ad35-GRIN was administered intramuscularly at 0 and 6 months. Participants were randomized to receive either vaccine or placebo (10/4 per group, respectively) within one of four dosage groups: Ad35-GRIN/ENV 2×10^9^ (A), 2×10^10^ (B), 2×10^11^ (C), or Ad35-GRIN 1×10^10^ (D) viral particles.

**Results:**

No vaccine-related serious adverse event was reported. Reactogenicity events reported were dose-dependent, mostly mild or moderate, some severe in Group C volunteers, all transient and resolving spontaneously. IFN-γ ELISPOT responses to any vaccine antigen were detected in 50, 56, 70 and 90% after the first vaccination, and in 75, 100, 88 and 86% of Groups A–D vaccine recipients after the second vaccination, respectively. The median spot forming cells (SFC) per 10^6^ PBMC to any antigen was 78–139 across Groups A–C and 158–174 in Group D, after each of the vaccinations with a maximum of 2991 SFC. Four to five HIV proteins were commonly recognized across all the groups and over multiple timepoints. CD4+ and CD8+ T-cell responses were polyfunctional. Env antibodies were detected in all Group A–C vaccinees and Gag antibodies in most vaccinees after the second immunization. Ad35 neutralizing titers remained low after the second vaccination.

**Conclusion/Significance:**

Ad35-GRIN/ENV reactogenicity was dose-related. HIV-specific cellular and humoral responses were seen in the majority of volunteers immunized with Ad35-GRIN/ENV or Ad35-GRIN and increased after the second vaccination. T-cell responses were broad and polyfunctional.

**Trial Registration:**

ClinicalTrials.gov NCT00851383

## Introduction

HIV/AIDS is a worldwide public health threat causing high morbidity and mortality. At the end of 2010, the total number of people living with HIV was estimated to be 34 million, up 17% from 2001. This reflects the continued large number of new HIV infections and a significant expansion of access to antiretroviral therapy, which has helped reduce AIDS-related deaths, especially in recent years [1]. Despite promising but still fragile successes in prevention, care and treatment, the development of a safe and efficacious preventive HIV vaccine, as part of a comprehensive prevention program remains a global health priority, and the best tool for long-term control of the HIV epidemic [2], [3]. Although the nature of the immune response needed to confer protection against HIV infection is unknown, an effective immune response will likely comprise antibodies and T cells that neutralize free virus and/or recognize and eradicate cells infected with diverse strains of HIV before an infection becomes irreversibly established [4].

Monomeric gp120 envelope subunits failed to induce neutralizing antibodies against circulating isolates and to confer protection against HIV acquisition [5], [6]. Generation of broadly neutralizing antibodies is still a challenge [7], [8] despite the recent progress in isolating broad neutralizing monoclonal antibodies against HIV [9], [10], [11], [12], [13], [14], [15], [16]. Recent efforts have focused on the development of HIV vaccines capable of inducing broad cell-mediated responses that could reduce viral replication after infection (“T-cell vaccines”) [17], [18]. Although contradicted by some studies [19], control of viral replication could slow the rate of disease progression, as suggested by non-human primate (NHP) challenge studies [20], [21], [22], [23], [24], and/or reduce transmission of HIV from infected vaccine recipient to partner by reducing virus load in the infected person [25].

Replication-incompetent viral vectors, including adenoviruses and poxviruses are among current strategies for induction of cell-mediated immune (CMI) responses in humans. The Step (HVTN 502/Merck 023) and Phambili (HVTN 503) vaccine trials were the first human efficacy trials (phase IIb ‘test-of-concept’) to explore whether a vector-based HIV-1 prophylactic vaccine aimed at inducing CMI responses could prevent infection or reduce post-infection viremia. The Merck vaccine was composed of replication-incompetent adenovirus serotype 5 (MRKAd5 HIV-1) vectors expressing HIV-1 clade B non-envelope antigens. The Step study enrolled, predominantly high-risk populations including men who have sex with men (MSM) as well as heterosexual women in North and South America and Australia, and heterosexual women and men in the Caribbean [26], [27]. The Phambili study enrolled heterosexual men and women in South Africa [28]. HVTN 502/Merck 023 was unexpectedly halted for futility in achieving the study primary endpoints (follow-up continued for two years after interim analysis) with an HIV incidence greater in vaccine than in placebo recipients, mostly men having sex with men and subjects with pre-existing Ad5-specific neutralizing antibody titers. The biological basis for this observation remains unclear. Post-hoc multivariate analysis further suggested that the greatest increased risk was in men who had pre-existing Ad5-specific neutralizing antibodies and who were uncircumcised [29], [30]. Although the MRKAd5 HIV-1 vaccine induced IFN-γ ELISPOT responses, and polyfunctional T cells by flow cytometry in the majority of recipients, it did not result in a decreased viral load in HIV-infected individuals [27]. Moreover, the immune response was lower both in frequency and magnitude in individuals with pre-existing Ad5 antibody titer >18 [27], [31].

Recently, a phase IIb trial (RV144) of ALVAC-HIV and AIDSVAX® gp120 B/E prime-boost enrolling Thai volunteers at “community risk” for HIV infection showed that, by modified intent-to-treat analysis 3.5 years after initial vaccination, the vaccine regimen was 31.2% efficacious in preventing HIV infection. Six months after the last vaccination, HIV Env- and Gag-specific IFN-γ ELISPOT responses were detected in 19.7% of vaccine recipients, Env-specific intracellular cytokine staining in 34%, and lymphoproliferative responses and binding antibodies to Env in a majority of subjects. There was however, no effect on early post-infection HIV-1 RNA viral load or CD4+ T-cell count [32].

This paper describes a clinical study with an HIV vaccine based on adenovirus serotype 35 (Ad35) that was designed to overcome pre-existing humoral immunity, a hurdle faced by Ad5-based vaccines. Ad35 is a human adenovirus serotype with low seroprevalence. In addition, the prevalence and titers of Ad35 neutralizing antibodies are lower than those of Ad5 neutralizing antibodies in Africa, Europe, North America and Asia [33], [34]. In adults, Ad35 seroprevalence was 10.6%–17.8% in South Africa, 14.8% in Kenya, 5.4% in Uganda, and 17.1% Thailand [35]. Ad35 belongs to subtype B of adenoviruses, which use highly expressed CD46 as receptor, while Ad5 is a subtype C using the coxsackie-adenovirus receptor (CAR) [36].

Recombinant Ad35-based vectors have been studied alone or in prime-boost regimens with DNA or with another Ad vector, as vaccines against HIV (NCT00479999: HVTN 072 and NCT00801697: HVTN 077), tuberculosis (AERAS) [37] and malaria (NCT01018459, NCT01366534, NCT00371189). The vaccines are generally well tolerated and immunogenic. This report describes a phase I dose-escalation, placebo-controlled, randomized study of two Ad35 vectors, Ad35-GRIN containing HIV-1 subtype A *gag*, *reverse transcriptase, integrase* and *nef* genes and Ad35-ENV containing HIV-1 subtype A *env* gene tested in healthy HIV-uninfected adults at low-risk of HIV acquisition.

### Participants

Healthy HIV-uninfected male and female adults aged 18–50 years were recruited at the University of Rochester, NY, USA, through information presented via the Internet, at community organizations, hospitals, colleges, other institutions and/or advertisements to the general public. Volunteers reported low-risk behavior for HIV (i.e., no unprotected vaginal or anal sex with known HIV-infected person; no sex in exchange for money or drugs; no sexually transmitted infection within 6 months before enrollment); and they were willing to undergo HIV testing and receive results. Sexually active women participants agreed to use effective contraceptive methods at least until 4 months after the second vaccination and not to become pregnant. Only subjects with negative baseline serum neutralizing antibodies against Ad35 were enrolled.

### Interventions

Two recombinant replication-incompetent adenovirus serotype 35 (Ad35) vectors, Ad35-GRIN and Ad35-ENV, were generated for this study. HIV-1 isolates used for the vector constructs were obtained from the National Institute for Biological Standards and Control repository, United Kingdom. Ad35-GRIN contains HIV-1 subtype A *gag* (derived from HIV-1 isolate 01TZA173, Tanzania), *reverse transcriptase, integrase*, and *nef* (derived from HIV-1 isolate 00KE_MSA4070, Kenya) genes, designed as a fusion product, and codon-optimized for human cell expression and translation. Mutations were introduced into the *reverse transcriptase* and *integrase* sequences to abrogate functional activity. Ad35-ENV is expressing HIV-1 subtype A gp140 *env* gene (derived from HIV-1 isolate 01TZA341, Tanzania). Ad35-GRIN/ENV consists of two vectors Ad35-GRIN and Ad35-ENV formulated in a 1∶1 ratio. The Ad35 vectors were produced on an E1-complementing human cell line (HER96). Vaccines and placebo were both manufactured according to the principles of Good Manufacturing Practices (GMP) by Transgene SA (Illkirch, France).

The Ad35-GRIN and Ad35-GRIN/ENV vaccines were prepared in a formulation buffer composed of Tris 10 mM pH 8.5, Sucrose 342.3 g/L, 1 mM MgCl2, Tween80 54 mg/L and 150 mM NaCl in water for injection and filled into single use vials for intramuscular injection and presented as frozen sterile solutions. The dosage of the vaccine is expressed as a total of virus particles (vp). The formulation buffer was used as placebo. Each vector, Ad35-GRIN and Ad35-ENV, was formulated at three dosage levels: 1×10^9^ vp, 1×10^10^ vp and 1×10×^11^ vp for a final dosage of Ad35-GRIN/ENV of 2×10^9^ vp (low dose, LD, Group A), 2×10^10^ vp (mid dose, MD, Group B) and 2×10^11^ vp (high dose, HD, Group C). Ad35-GRIN alone was administered at 1×10^10^ vp (Group D). Ad35-GRIN/ENV and Ad35-GRIN were administered in a volume of 0.5 mL by needle injection in the deltoid muscle at 0 and 6 months. Participants were randomized within a dosage group to receive either vaccine or placebo in a 10∶4 ratio. Each dosage group was enrolled sequentially in dose-escalation manner. Group D was enrolled after all other groups following an amendment to the original protocol. The demographic characteristics of the study population are described in **Table 1**. The study screening was initiated in March 2009 and completed in August 2011, volunteers were followed for 18 months one year after the second immunization.

**Table 1:** Demographic Characteristics of the Study Population.

		Group A	Group B	Group C	Group D	
		Ad35- GRIN/ENV			Ad35-GRIN	
	Placebo	2×10^9^ vp	2×10^10^ vp	2×10^11^ vp	1×10^10^ vp	Total
Number of Volunteers	16	10	10	10	10	56
**Sex**						
Female	5 (31.3%)	3 (30.0%)	4 (40.0%)	7 (70.0%)	3 (30.0%)	22 (39.3%)
Male	11 (68.8%)	7 (70.0%)	6 (60.0%)	3 (30.0%)	7 (70.0%)	34 (60.7%)
**Race**						
American Indian	1 (6.3%)	0	0	0	1 (10.0%)	2 (3.6%)
Asian	1 (6.3%)	0	1 (10.0%)	0	0	2 (3.6%)
Black	0	2 (20.0%)	0	3 (30.0%)	0	5 (8.9%)
White	14 (87.5%)	8 (80.0%)	9 (90.0%)	7 (70.0%)	9 (90.0%)	47 (83.9%)
**Ethnicity**						
Hispanic or Latino	0	0	2 (20.0%)	0	2 (20.0%)	4 (7.1%)
Not Hispanic and Not Latino	16 (100.0%)	10 (100.0%)	8 (80.0%)	10 (100.0%)	8 (80.0%)	52 (92.9%)
**Age (yrs)**						
Mean	31.0	24.2	24.6	29.2	24.0	27.1
Range	18–48	19–44	18–34	20–43	20–29	18–48
**Vaccinations Received**						
First Vaccination	16 (100.0%)	10 (100.0%)	10 (100.0%)	10 (100.0%)	10 (100.0%)	56 (100.0%)
Second Vaccination	16 (100.0%)	9 (90.0%)	8 (80.0%)	9 (90.0%)	8 (80.0%)	50 (89.3%)
**Follow-up Status**						
Completed	15 (93.8%)	8 (80.0%)	8 (80.0%)	9 (90.0%)	8 (80.0%)	48 (85.7%)

### Objectives

The primary objective of the study was to evaluate the safety and tolerability of a recombinant Ad35 vectors expressing multiple HIV-1 proteins at different dosage levels, administered to healthy HIV-uninfected adults. Shedding of Ad35-GRIN/ENV and Ad35-GRIN after vaccination was also studied. The secondary objective was to evaluate the humoral and cellular immunogenicity of the vaccines at each dosage level and to explore a possible Env immunodominance by comparing immune responses directed against GRIN proteins between Groups B and D.

## Materials and Methods

The protocol for this trial and supporting CONSORT checklist are available as supporting information; see Checklist S1 and Protocol S1.

### Ethics Statement

This study was approved by the Western Institutional Review Board (WIRB). The study was conducted in accordance with International Conference on Harmonization - Good Clinical Practice (ICH-GCP) and Good Clinical Laboratory Practice (GCLP) [38]. All participants provided written informed consent.

### Safety Monitoring

Study participants were monitored by interim medical history, and by physical and laboratory assessments. Local (pain, tenderness, erythema/skin discoloration, induration, vesicle/ulceration, crust or scab) and systemic signs and symptoms (fever, chills, headache, nausea, vomiting, malaise, myalgia, arthralgia) were solicited for 14 days after each vaccination. Subjects were evaluated at the vaccination clinic on day 0 (pre- and 30 minutes post-vaccination) and on days 3, 7 and 14 post each vaccination to review their memory aids entries and record symptoms at that time (Clinic Assessments). In addition, study subjects were given 14-day memory aid cards and instructed to record their maximal symptoms experienced each day (Participant Assessments). Unsolicited adverse events (AEs) were recorded throughout the study, graded for severity (grade 1 = mild, grade 2 = moderate, grade 3 = severe, grade 4 = potentially life-threatening according to the Division of AIDS Adult Adverse Event Grading Toxicity Tables, version 1.0, December 2004) and classified by MedDRA (Medical Dictionary for Regulatory Activities). The AEs were assessed for relationship to study vaccines. There were five categories of relatedness: definitely, probably, possibly, unlikely and not related. Protocol deviations were monitored throughout the trial. The Safety Review Board (SRB) authorized the dose escalation after review of safety data of the lower dose group, based on a compilation of blinded data from the first 9 volunteers enrolled. Any severe and very severe events were provided to the SRB as an update prior to proceeding with the next dosage level.

### Ad35 Shedding

Ad35-GRIN/ENV and Ad35-GRIN shedding was studied by collecting oro-pharyngeal swabs and urine specimens in a subset of volunteers at days 0, 3, and 14 post 1st injection and prior to booster injection at month 6. In addition, specimens for adenovirus investigation were collected as clinically indicated within the first 14 days post immunization for any reported respiratory, genito-urinary, or diarrheal illness or conjunctivitis, unless another cause was revealed by the diagnostic investigations. Pairs of synthetic PCR primers specific for the ENV and GRIN inserts were used to identify samples that might contain the vaccine vector. Specimens were frozen on-site and batch tested using a molecular diagnostic PCR assay by Esoterix Clinical Trial Services (Cranford, NJ, USA).

### Testing Algorithm for HIV Infection

Study participants were tested for the presence of antibodies to HIV-1 and HIV-2 at the University of Washington Virology Specialty Laboratory (Seattle, WA, USA) using the Abbott HIVAB HIV-1/HIV-2 ELISA kit (Abbott Park, IL, USA) during study screening (within 42 days of enrollment) and on the day of enrollment prior to the first injection. Subsequently, at study weeks 4, 24 (prior to second injection), 32, and 52 serologic testing was performed for all participants at a blinded laboratory using the Bio-Rad Genetic Systems HIV 1/2 Plus O ELISA kit (Redmond, WA, USA). If a post-enrollment ELISA was positive, additional testing was done for the participant to distinguish vaccine-induced responses from true infection, including a repeat Bio-Rad Genetic Systems HIV 1/2 Plus O ELISA, a Bio-Rad Genetic Systems HIV-1 Western Blot (Redmond, WA, USA) and an Abbott m2000 Real Time PCR HIV-1 RNA kit (Abbott Park, IL, USA). Subsequently, at the final study visit, a full diagnostic panel of serologic and virologic testing was conducted for all participants in order to inform them of the likelihood of subsequent confusion regarding the use of HIV serology for diagnostic purposes. This panel included a Bio-Rad Genetic Systems HIV 1/2 Plus O EIA (Redmond, WA, USA), Abbott Architect HIV Ab/Ag Combo EIA (Abbott Park, IL, USA), Bio-Rad Genetic Systems HIV-1 Western Blot (Redmond, WA, USA), and Abbott m2000 Real Time PCR HIV-1 RNA kit (Abbott Park, IL, USA).

### Immunology Assays

All the primary immunogenicity assays (IFN-γ ELISPOT, Ad35 neutralization and Env/Gag ELISA) are validated and run according to GCLP guidelines, and the flow cytometry assay is qualified. The IAVI Human Immunology Laboratory (HIL) participates in EQA and/or IQA panels for IFN-γ ELISPOT, flow cytometry and PBMC preparation and passes the required criteria [39], [40], [41].

### Ad35 Neutralizing Antibody Assay

Presence of pre-existing antibody to Ad35 at screening was a criterion for exclusion and was determined for each volunteer prior to enrollment. Anti-Ad35 neutralization titers were measured using heat-inactivated serum samples from 4 weeks after the first vaccination and 2 weeks after the second vaccination in a previously described, qualified cell-based assay [42]. Briefly, A549 cells were seeded in a 96 well plate in the presence of serially diluted serum. Following addition of luciferase-encoding Ad35 reporter virus and incubation at 37°C for 24 hours, luciferase activity in cell lysates was measured using a Victor3 multi-label plate reader (Perkin Elmer, Waltham, MA, USA) upon addition of a substrate. Anti-Ad35 titers were calculated as the serum dilution allowing a 90% reduction of luciferase activity in infected cells (EC90). An EC90 cutoff of 16 was set where a positive response was defined as EC90 ≥16 and a negative response as EC90<16. Samples for the Ad35 neutralization assay or the EnvA ELISA were run batched by groups and visits. Only samples used to determine pre-existing immunity to the Ad35 were run immediately upon receipt from the clinic to allow speedy enrolment. For the Ad35 neutralization, each run includes titration of a known positive control and negative control sera. The titer from the positive control allows a comparison between assays. Similarly, known positive and negative controls were run on each ELISA plate.

### HIV-specific Binding Antibodies

An ELISA assay was used to measure HIV specific Env-gp140 and Gag-p24 antibody responses at baseline and at indicated times post-vaccination (**Table 2**). End-point titration of serum was performed in 96-well medium binding plates (Greiner Bio-one, Frickenhausen, Germany) coated with preparations of 2.5 µg/mL purified recombinant subtype B Gag p24 (Aalto Bio Reagents Ltd., Dublin, Ireland) or 5 µg/mL subtype A Env UG037 (Polymun Scientific Immunbiologische Forschung, Vienna, Austria). Titers were determined by sequential incubation of antigen with serum followed by HRP-labeled anti-human IgG and TMB (3, 59, 5, 59-tetra-methylbenzidine) substrate. After addition of stop solution, the optical density (OD) at 450 nm was measured for 5-fold serially diluted samples starting at 1/100. The titer was calculated as the most dilute serum concentration above the OD cut off of 0.3 and 0.2 respectively for Env and Gag p24 and reported as reciprocal dilution.

**Table 2:** Number of Volunteers Enrolled, Vaccination Schedule, Sample Collection and Assessment time points.

Group^a^	Vaccines/Dosage	Pre^b^	W0	W2	W4	W24	W25	W26	W28	W32	W38	W52, 64 & 72
**A (**10/4)	Ad35-GRIN/ENV 2×10^9^ vp	c	d,e,f	d	c,d,e	d,e	d	c,d,e,f	d,e	d	d,e	d,e
**B** (10/4)	Ad35-GRIN/ENV 2×10^10^ vp	c	d,e,f	d	c,d,e	d,e	d	c,d,e,f	d,e	d	d,e	d,e
**C** (10/4)	Ad35-GRIN/ENV 2×10^11^ vp	c	d,e,f	d	c,d,e	d,e	d	c,d,e,f	d,e	d	d,e	d,e
**D** (10/4)	Ad35-GRIN 1×10^10^ vp	c	d,e,f	d	c,d,e	d,e	d	c,d,e,f	d,e	d	d,e	d,e

W  =  week, W0 and W24 are vaccination visits.

a (V/P) Number of Vaccine recipients/number of Placebo recipients per group.

b Screen window up to 65 days prior to enrollment for anti-Ad35 antibodies.

c Serum neutralizing antibodies against Ad35.

d Vaccine-induced HIV-1 specific IFN-γ ELISPOT responses.

e Vaccine-induced HIV-1 specific humoral immune responses (Env and p24 Gag ELISA).

f Polychromatic Flow Cytometry.

### HIV-1 Neutralizing Antibodies

Sera were tested for neutralizing activity against HIV-1 two weeks post-second vaccination at Monogram Biosciences, Inc. (South San Francisco, CA, USA) as described elsewhere [43]. The high-throughput assay utilizes a panel of recombinant viruses pseudotyped with HIV envelope proteins covering a range of neutralization sensitivity and geographic diversity as follows; MGRM-A-001, MGRM-A-003, 94UG103 and 92RW020 (subtype A); JR-CSF, NL-4-3 and SF162 (subtype B); MGRM-C-026 (subtype C) and 94UG114 (subtype D). Neutralizing activity is expressed as the percent inhibition of viral replication (luciferase activity) at each antibody dilution compared with an antibody-negative control. Titers were calculated as the reciprocal of the plasma dilution conferring 50% inhibition (IC_50_).

### Peripheral Blood Mononuclear Cell Sample Preparation and Peptide Stimuli

Peripheral blood mononuclear cells (PBMC) were isolated using density gradient separation from heparinized whole blood, frozen in a mixture of fetal bovine serum (Sigma-Aldrich, St Louis, MO, USA) and DMSO (90∶10 ratio) using a Kryo 560-16 rate controlled freezer (Planer, Sunbury-On-Thames, UK). PBMC were stored and shipped in vapor phase liquid nitrogen to the IAVI HIL, Imperial College, London [44], [45]. PBMC were thawed, overnight rested and counted using a Vi-Cell XR counter (Beckman Coulter, UK) for ELISPOT and flow cytometric analyses using 6 HIV-1 15mer peptide pools, overlapping by 11 at 1.5 µg/mL per peptide, matching the vaccine inserts; one pool each representing Gag, Pol/Int, RT, Nef and 2 pools representing the Env sequence were used as stimuli.

### IFN-γ ELISPOT Assay

A validated IFN-γ ELISPOT assay was the primary immunogenicity readout for this study and was conducted on participant specimens obtained just prior to each vaccination, at weeks 2 and 4 after each vaccination and other timepoints as indicated in **Table 2**. PBMC were plated in quadruplicate at 2×10^5^ viable cells per well in the IFN-γ ELISPOT assay with HIV-1 peptide pools at 1.5 µg/mL, a peptide pool consisting of Cytomegalovirus (CMV) pp65 peptides also at 1.5 µg/mL, PHA at 10 µg/mL and a mock stimulus (DMSO/medium) as previously described [44], [45]. Spot forming cells (SFC) were counted using an automated AID ELISPOT reader (Autoimmun Diagnostika, Strassberg, Germany). The number of SFC/10^6^ PBMC had to satisfy the following criteria: 1) Average number of background-subtracted spots in a single pool >38 SFC; 2) For each pool, if the number of replicates is greater than one, the coefficient of variation (Standard Deviation/Mean) between replicates should be <70%; 3) Mean count must be >4 times mean background; 4) Mean background must be ≤50 SFC/10^6^ PBMC. Assays with mean background >55 SFC/10^6^ PBMC were considered failures and excluded from further analysis if a repeat test confirmed this. In cases where a response to a peptide pool was found to be positive at baseline, subsequent responses to this pool were not included in the determination of response rate.

The IAVI HIL has a validated ELISPOT assay and samples are run in batches in approximate chronological order of receipt. To ensure stability of the assay over time, a replicate reference quality control (QC) PBMC is run on each assay day, and an internal QC CMV antigen and PHA for each volunteer immunogenicity study visit is run. The coefficient of variation (CV) has remained stable over time with a CV of <30%. Each volunteer’s CMV, PHA and mock responses have remained stable over time with low CV.

### Polychromatic Flow Cytometry

The antigen-specific phenotype and cytokine secretion profiles were assessed at baseline and 2 weeks post-second vaccination in Groups B-D using a qualified polychromatic flow cytometry (PFC) panel. PBMC were co-incubated with HIV-1 peptide pools, 1 µg/ml SEB (Sigma-Aldrich, St. Louis, MO, USA) or mock stimuli, CD107a PECy5, BD Golgistop (Becton Dickinson, San Jose, CA, USA) and Brefeldin A (Sigma-Aldrich, Poole Dorset, UK) for 6 hours at 37°C. Cells were stained for viability with 100 µL LIVE/DEAD® Fixable Violet Dead Cell Stain Kit (Invitrogen, Eugene, OR, USA), and then surface stained by anti-CD4 QD605, anti-CD8 pacific orange, anti-CD19 pacific blue (Invitrogen, Paisley, UK), anti-CD27 APC-H7, anti-CD14 pacific blue, anti-CD57 FITC, anti-B7 integrin PE (Becton Dickinson, San Jose, CA), and anti-CD45RO ECD (Beckman Coulter, High Wycombe, UK). Finally cells were stained intracellularly with anti-CD3 QD655 (Invitrogen, Paisley, UK), anti-IFN-γ PE Cy7, anti-TNF-α A700 and anti-IL-2 APC (Becton Dickinson, San Jose, CA, USA). At least 750,000 events were acquired on a custom-built BD LSR II cytometer. Data were analyzed and presented using FlowJo (version 8.8 Treestar), PESTLE and SPICE (version 5.1, http://exon.niaid/nih.gov/SPICE/) software [46]. The percentage of cytokine-producing cells after antigen stimulation was considered positive if it fulfilled the following three criteria: 1) the response was at least two times greater than the percentage of cytokine-producing cells in the mock pool at the same post-vaccination time point, 2) the response to the same antigen was negative at pre-vaccination baseline, and 3) for each cytokine the response was superior or equal to the 97.5 percentile of all baseline responses to that peptide (Groups B–D). Data were not included for analysis if cell viability was less than 80% at the time of assay set-up or if fewer than 50,000 events were present in the CD3+ gate or less than 10,000 events in either CD4+ or CD8+ gates. In order to be included in the sample analysis, all samples had to test positive for cytokine production when stimulated by staphylococcal enterotoxin B (SEB, Sigma-Aldrich, St Louis, MO, USA).

For the PFC assay, batches of baseline and matched post-vaccine samples are thawed on the same day and data accrual is performed within 18 hours. Daily QC of the LSRII is performed as described previously [47]. Daily voltages are tracked over time to ensure consistency between individual runs. Additionally, the responses to CMV antigen of a control PBMC sample are pre-determined by testing over 20 individual timepoints. An aliquot of this control is then run in parallel to clinical trial samples to ensure that responses do not vary more than 2 SD during the study.

### Sample Size and Pause Rules

#### Safety interim analyses

Blinded summary tables and listings of adverse events, including solicited reactogenicity events, were presented to an independent Safety Review Board (SRB). Approval for dose-escalation was made by the SRB after review of unblinded two-week safety data after first injection from the first nine participants in Groups A and B.

#### Randomization and blinding

This study was a double-blind, randomized, placebo-controlled, dose-escalation Phase I clinical trial. The randomization schedule was prepared by statisticians at the Data Coordinating Center, EMMES Corporation. The randomization list was sent to the site pharmacist of record for dispensing of vaccine and placebo in a double-blind fashion. Study site staff, volunteers, and laboratories remained blinded with respect to the allocation of placebo or vaccine.

#### Statistical methods

The planned sample size of 56 volunteers (40 vaccine/16 placebo) was considered to be appropriate for an exploratory clinical trial for evaluating safety while also providing relevant information on vaccine-induced immune responses. If none of the 10 volunteers in any active vaccine group experienced a serious adverse event related to the Investigational Product, then the upper 95% confidence limit for the rate of these adverse events in the population was 30.8% (by the Clopper-Pearson method). Due to the small sample size, the trial had limited power to rule out smaller differences in safety and immunogenicity results. For comparison of active vaccine (N = 40) versus placebo (N = 16), there was 80% power to detect a statistically significant (p<0.05) difference of 28.5% and 32.7% if the event rate in the placebo group was 1% or 5%, respectively (Fisher’s exact 1-tailed test). Fisher’s exact test (for categorical variables) and Kruskal-Wallis Test (for continuous variables) were used to compare the balance and/or values of baseline characteristics between the study groups. All safety and immunogenicity comparisons were made using Fisher’s exact, 2-tailed tests of the proportions of volunteers with an endpoint, unless otherwise stated. The safety comparisons were based on the maximum severity per volunteer. All tests are 2-tailed; statistical significance is defined as a p<0.05. However, when pairwise comparisons among the study groups were conducted, p<0.01 was considered to be statistically significant. Analyses were performed using SAS version 9.2, (SAS, Cary, NC, USA).

## Results

### Participant Flow and Recruitment

**Figure 1:**
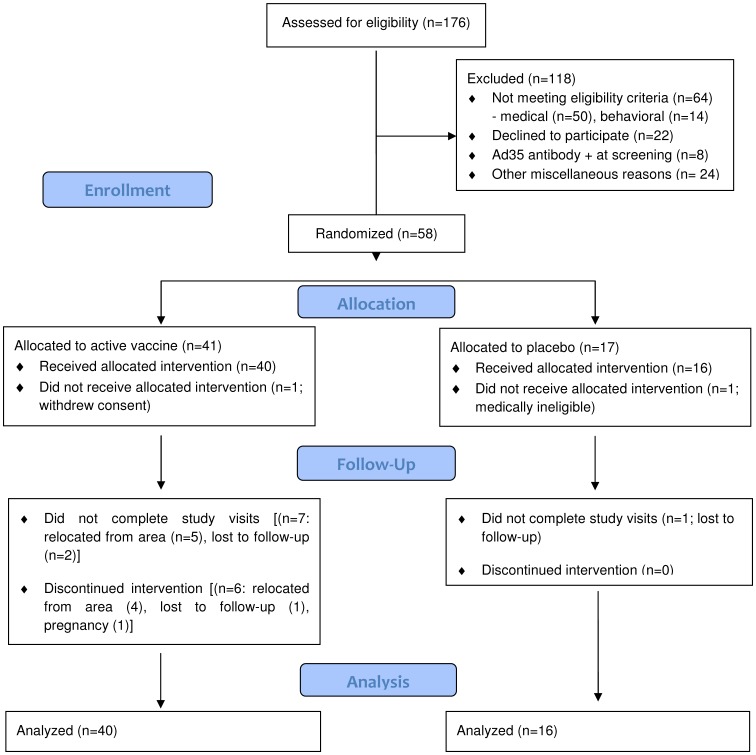
CONSORT Flow Diagram. Number of individuals assessed for eligibility, enrolled and randomized to study vaccine(s) and respective placebo, followed-up and analyzed.

A total of 176 adults between 18-50 years of age were screened at the study site in order to enroll 56 participants into the protocol (**Figure 1**). The most common reasons for not qualifying for the study were 1) did not meet the eligibility requirements due to medical and/or behavioral reasons (n = 64, 36.4%), 2) declined to participate after receiving a full explanation of study procedures (n = 22, 12.5%) and 3) presence of pre-existing anti-Ad35 neutralizing antibodies at screening (n = 8, 4.5%). Six enrolled study participants did not receive the second injection at 6 months (however two of them completed the study follow-up; 1 due to pregnancy after the 12-week study visit and the other due to relocation that made attendance at the frequent early post-vaccination follow-up visits impossible). Eight subjects did not complete study visits, five for personal reasons and three for loss to follow-up (**Figure 1**). **Table 1** describes the demographic characteristics of the enrolled volunteers.

### Protocol Deviations

There were 123 minor protocol deviations in this study, mostly involving isolated inability to obtain complete collections of biological specimens (i.e., shortage of blood volume due to poor venous access or lack of urine specimen due to subjects’ inability to void), or minor deviations involving protocol-specified study visit windows or schedule compliance. In addition, routine oro-pharyngeal specimens for Ad35 viral shedding were not collected from the first 9 and first 5 participants in study Groups B and C, respectively, but adequate routine specimens were collected from the first 9 subjects in study Groups A and D and from all study subjects who presented with respiratory tract symptoms. The interpretation of the data presented here is not affected by the protocol deviations.

### Vaccine Safety

#### Solicited events

**Figure 2:**
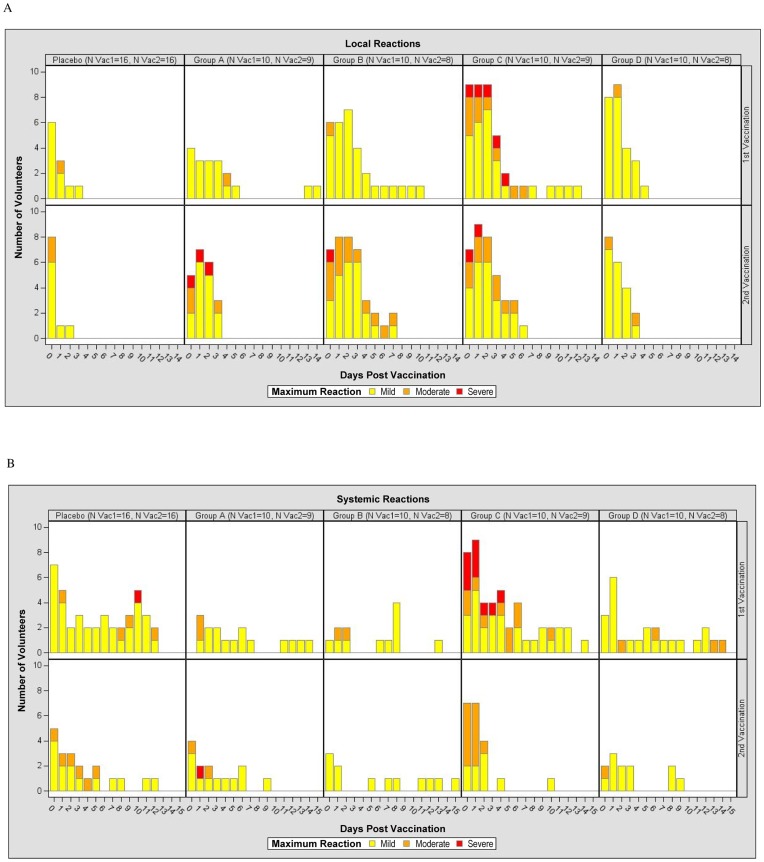
Time Course of Local and Systemic Reactions Post First (Vac1) and Post Second (Vac2) Vaccination per Maximum Severity Assessment for Placebo, Group A (2×10^9^ vp), Group B (2×10^10^ vp), Group C (2×10^11^ vp) and Group D (1×10^10^ vp). The Y-axis of Figure 2 represents the number of volunteers experiencing reactogenicity events (panel A for local reactions and panel B for systemic reactions post first and second vaccinations, upper and lower rows respectively) for each group, while the X-axis represents the days of occurrence of the events, Day 0 being the day of vaccination. Volunteers did a self-assessment of reactogenicity with a memory card on Day 0 (evening of vaccination) and daily through Day 13, reviewed by the investigator at Days 3, 7 and 14. The figure shows the maximum severity assessment grade recorded as per the volunteer’s and clinic’s assessments combined. The severity grade of the reactogenicity events is indicated by color codes (mild: yellow; moderate: orange; severe: red).

The time course by study group for maximal symptoms localized to the injection site and for maximal systemic symptoms after each injection is shown in **Figure 2**, panels A and B, and panels C and D, respectively.

#### Local Reactogenicity

The majority of local vaccination-site reactions graded as mild or moderate after the first and second injections; the overall frequency of any local reaction was 80% in Group A, 100% in Groups B, C and D, and 81.3% among the placebo recipients (**Figure 2A**, Table S1 ****). Severe reactions were observed in one subject from Group A post-second vaccination (pain and tenderness), one subject from Group B post-second vaccination (pain), and three subjects in Group C: two post-first vaccination (pain and tenderness in both) and two subjects (one of them with reactogenicity post-first vaccination) post-second vaccination (tenderness). These reactions were self-limited and resolved within 1–3 days. The occurrence of moderate or severe reactions did not seem to increase after the second vaccination in any dose group. Erythema at the injection site was unusual in all groups. Induration (present, less than grade 1) was observed in one volunteer post-second vaccination in Group A. Formation of a scab (present, less than grade 1) was observed in one volunteer post-second vaccination in Group B and in one volunteer post-first vaccination in Group D, each resolving within 1–3 days.

#### Systemic reactogenicity

Overall, the frequency of any systemic reaction was 60% in Group A, 70% in Group B, 100% in Group C, 80% in Group D, and 68.9% in Placebo. These reactions were mild or moderate among participants in Groups A, B and D, except in one volunteer in Group A who had severe myalgia, arthralgia and malaise post-second vaccination In addition, one placebo recipient reported a severe headache after the first vaccination. Of note, 5 subjects (50%) in Group C, who received the highest dose, had severe systemic reactions that resulted in cancellation of scheduled activities (**Figure 2B** **, Table S2)**. As assessed by the volunteer and validated by the clinic, of those who had severe symptoms in Group C, one had symptoms that lasted an unusually long time: severe malaise, chills, myalgia and headache for 5 days, with severe injection-site pain and tenderness for 3 days, all beginning on Day 0, and fully resolving at Day 8. The other four subjects experienced more brief symptoms. One had severe myalgia on Day 1, which resolved the next day; one volunteer had severe chills, malaise and fever (39.9°C) on Day 1 only. Another volunteer had severe chills and malaise with moderate fever (38.9°C) at Day 1. The other subject had severe headache and pain for one day and severe tenderness for 2 days. Among these latter four subjects, all reactions began about 12 hours after vaccination, diminished in severity over the ensuing 12 hours, and resolved or became mild within 2–3 days. The participant who experienced the prolonged ‘flu-like illness’ was tested at day 3 after onset for influenza as pandemic H1N1 influenza was known to be circulating in the community at the time, but oro-pharyngeal culture and PCR were negative. Evidence of intercurrent influenza illness was also sought for all other study participants who experienced moderate to severe systemic symptoms after vaccination, but all results were negative. Of note, all subjects in Group C (except for the woman who became pregnant) agreed to receive their second vaccination at 6 months, which was much better tolerated with mostly mild and moderate reactions (Table S11 and S2).

#### Ad35 shedding

No Ad35 shedding, as assessed by recombinant vector-specific PCR, was detected in oro-pharyngeal swabs and urine specimens obtained routinely from any study group (328 samples: 130, 4, 46 and 148 in Groups A, B, C and D, respectively). In addition, no oro-pharyngeal swab samples tested from volunteers presenting with upper or lower respiratory symptoms (99 samples: 18, 10, 43 and 78 in Groups A, B, C and D, respectively) were positive for Ad35.

#### Unsolicited adverse events

A total of 171 non-serious adverse events (AEs) were reported by study participants during the course of the study (61 in Placebo, 25 in Group A, 27 in Group B, 30 in Group C and 28 in Group D). The frequency was not dose-related. One hundred and eight events were graded as mild (grade 1) in severity and 61 were graded as moderate (grade 2). Only 2 events were assessed as severe (grade 3): deep vein thrombosis and anxiety disorder; both were considered unrelated to the vaccine. Ten events were considered as possibly related to vaccine (diarrhea, injection site hemorrhage, 2 influenza-like illness, upper respiratory tract infection, pharyngo-laryngeal pain, pharyngitis, naso-pharyngitis, and 2 nasal congestion), one as probably related (injection site anesthesia) and one definitely related (injection site swelling). All other events were considered unrelated or unlikely to be related to vaccination.

#### Laboratory abnormalities

Moderate or greater abnormal clinical laboratory values were observed in five volunteers: elevated aspartate aminotransferase of moderate grade in two subjects (one in Group A prior to second vaccination and one in Group B 98 days post-second vaccination), and moderate grade low hemoglobin in two female participants. One subject from Group D had severe elevation of alanine aminotransferase 24 weeks after the first injection, just prior to the second vaccination. All of these abnormalities were judged to be unrelated to the study vaccinations and returned to values within the normal range during study follow-up.

#### Serious adverse event

Two Serious Adverse Events (SAE) were reported during the study; both were unrelated to vaccination. One was a subject who was hospitalized for accidental trauma of the knee. The second SAE was a subject with cholelithiasis which was treated by cholithectomy.

#### Social impact

One participant experienced difficulties in enlisting into the Armed Services due to vaccine-induced HIV seropositivity (VISP). After intervention of the research team, and of a representative from the US Military HIV Research Program for additional HIV testing, the participant was confirmed HIV-uninfected and able to join the Armed Services approximately six months later. Another participant was found to be seropositive for HIV as a part of routine health care visit having failed to mention his/her participation in the study. This person was also found to have VISP rather than true infection when the study team was consulted. Lastly a third participant attempted to donate blood and was denied due to his/her HIV status. Through additional testing this individual had VISP, but is no longer able to donate blood due to the current Red Cross guidelines.

#### Pregnancies

One woman in the high-dose Ad35-GRIN/ENV group who utilized an intrauterine device for contraception was found to be pregnant 5 months after the first vaccination. She did not receive her second study vaccination but continued with follow-up and completed the study on schedule. She delivered a healthy full-term baby.

#### Incident HIV infections

No incident HIV infection was detected among enrolled study participants throughout the study.

### Vaccine Immunogenicity

#### IFN-γ ELISPOT response

IFN-γ ELISPOT was the primary assay assessing cellular responses among groups in this study and was performed longitudinally as indicated in **Table 2** **,** responses at weeks 25, 32 and 52 are not reported here. The median percent viability of 599 PBMC samples after processing was 97.8% (range 82–100%); after storage, shipment and thawing of PBMC for the ELISPOT assay, the median percent viability of 565 PBMC samples up to 1 year after vaccination was 97.7% (range 77.7–99.7%). Eleven of 600 (<2%) ELISPOT samples tested failed to meet laboratory quality criteria.

#### Frequency of positive responses

The frequency of participants by study group with positive IFN-γ ELISPOT responses 2 and 4 weeks after each vaccination, as well as 14 and 48 weeks after the second vaccination is shown in Table 3. IFN-γ ELISPOT responses to any vaccine antigen were detected in 50%, 50% and 78% at 2 weeks and in 50%, 56% and 70% of vaccine recipients at 4 weeks post-first vaccination, in groups A, B and C respectively. Responses to any vaccine antigen in groups A–C were also detected in 86%, 100% and 89% of vaccine recipients at 2 weeks post-second vaccination and in 75%, 100% and 88% of vaccine recipients at 4 weeks post-second vaccination, respectively. Responses to Ad35-GRIN alone (Group D) were present in 89% and 90% of participants at 2 and 4 weeks after the first vaccination and in 86% of participants 2 and 4 weeks after the second vaccination. At 48 weeks after the second vaccination, IFN-γ ELISPOT responses to any vaccine antigen in Groups A–D were detected in 57%, 100%, 63% and 88% of vaccine recipients, respectively.

**Table 3:** Overall IFN-γ ELISPOT response rate for the 6 peptide pools in vaccine groups at selected time points with cryopreserved PBMC.

	Ad35-GRIN/ENV	Ad35- GRIN/ENV	Ad35-GRIN/ENV	Ad35-GRIN
Time Point	2×10^9^ vp	2×10^10^ vp	2×10^11^ vp	1×10^10^ vp
Overalla, b, c	10/10 (100%)	9/10 (90%)	10/10 (100%)	9/10 (90%)
Pre-Vaccination	0/9	0/10	0/10	0/10
2 weeks Post 1^st^ vaccination	4/8 (50%)	5/10 (50%)	7/9 (78%)	8/9 (89%)
4 weeks Post 1^st^ vaccination	5/10 (50%)	5/9 (56%)	7/10 (70%)	9/10 (90%)
2 weeks Post 2^nd^ vaccination	6/7 (86%)	8/8 (100%)	8/9 (89%)	6/7 (86%)
4 weeks Post 2^nd^ vaccination	6/8 (75%)	7/7 (100%)	7/8 (88%)	6/7 (86%)
14 weeks Post 2^nd^ vaccination	5/9 (56%)	8/8 (100%)	7/9 (78%)	7/8 (88%)
48 weeks Post 2^nd^ vaccination	4/7 (57%)	8/8 (100%)	5/8 (63%)	7/8 (88%)

a Not all samples from all time points were tested due to assay failure, missed visits or volunteer withdrawal.

b A volunteer is defined as a responder if they score positive to any pool.

c One placebo recipient had a positive ENV ELISPOT at baseline; otherwise all tests for placebo recipients were negative (data not shown in table).

No assays were positive among vaccine recipients prior to the first vaccination. There was one placebo recipient with a low ENV response at baseline, which persisted at all post-vaccination timepoints. Conversely, considering all timepoints examined with this assay during the study, only 2 of 40 vaccine recipients failed to have a positive response during at least one visit. Overall IFN-γ ELISPOT response rate in vaccine recipients (90%) was significantly higher than the rate in placebo recipients (0%) at 2 weeks post second vaccination (p<0.0001).

Repeatedly positive IFN-γ ELISPOT responses over time among individuals in all vaccine groups were commonly observed; the median number of positive assays per participant was 7.5, 9, 9 and 10 over 8–10 available visits for Groups A–D, respectively (data not shown). Breadth of the response as defined by the median number of positive responses among vaccinees to any of the six peptide pools for Groups A–C was 3.5, 5 and 5, respectively. For Group D, where only 4 peptide pools were tested, the median number of positive responses among vaccinees was 4. **Figure S1** shows the frequency and distribution of peptide pool responses over time in each group, providing an additional perspective of the breadth of the response to the vaccine regimens. In this case, five pools are included in this analysis; Gag, Pol/Int, RT, Nef and Env pools. Responses of up to 4 pools were commonly observed across all the groups and over multiple timepoints indicating that good breadth was seen early and persisted over time.

### Magnitude of Positive Responses

**Figure 3:**
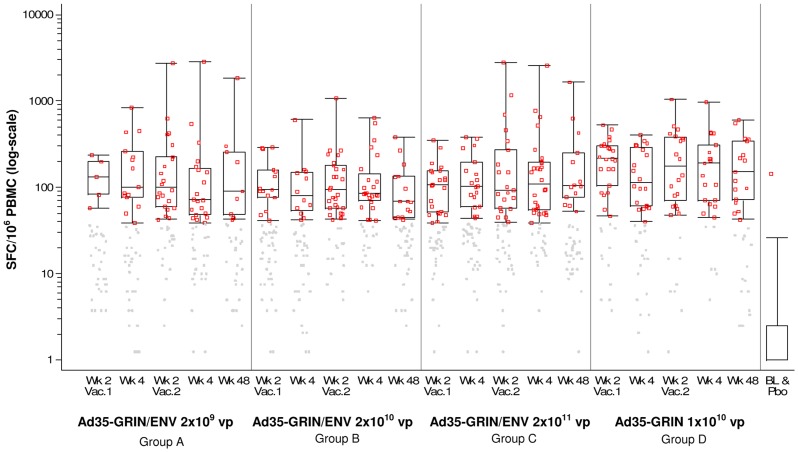
IFN-γ ELISPOT Response Magnitude (SFC/10^6^ PBMC) and Responder Rate (%) to Any HIV Antigen by Time Post Vaccination (X-axis) and Dose Groups. Gray dots: response below the cut-off to any of the 6 peptide pools; red dots: response above the cut-off to any of the 6 peptide pools. For the vaccine groups, the overlaid box plot summarizes the positive responses (i.e., the median, 1^st^ and 3^rd^ quartiles and minimum/maximum). For the baseline (BL) and placebo (Pbo) group, the box plot summarizes the negative responses and the red dot displays the single positive response at baseline.

**Figure 4:**
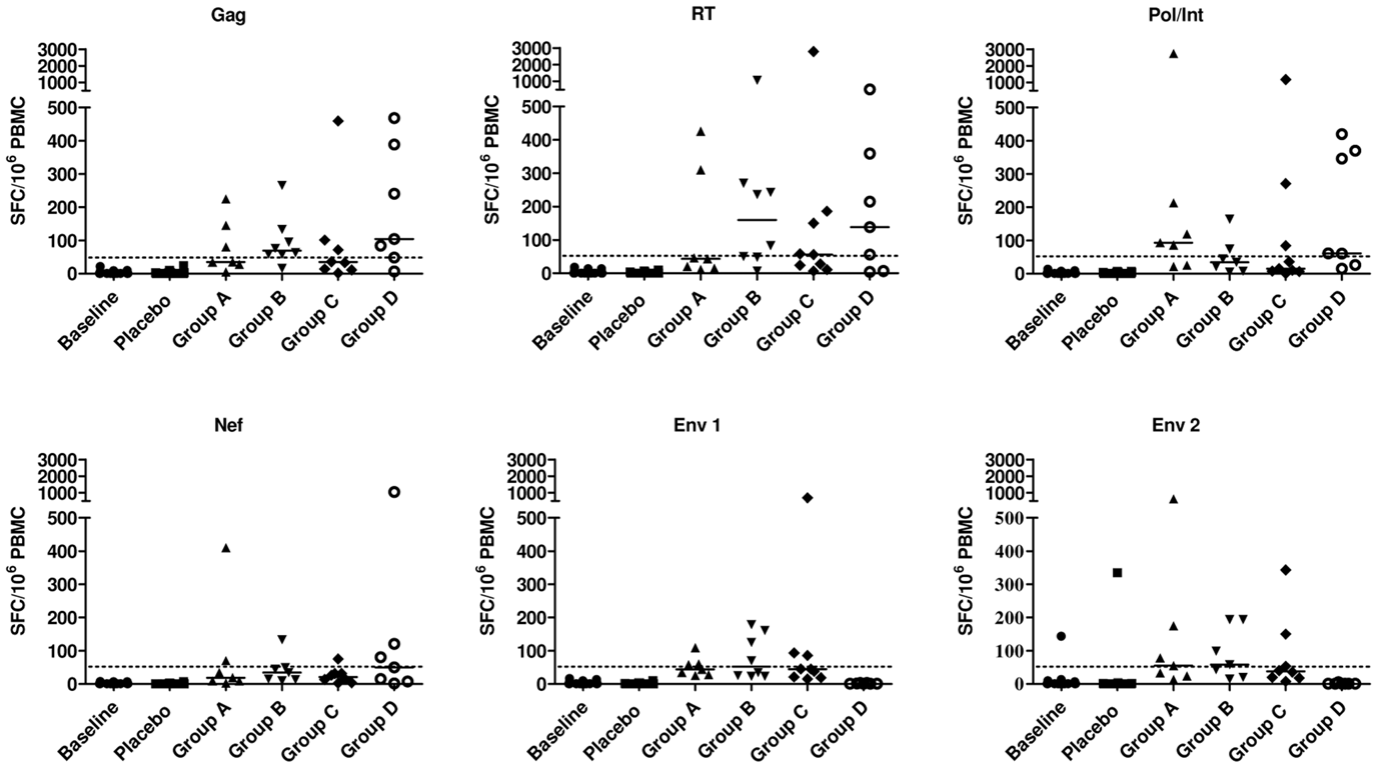
IFN-γ ELISPOT Response Magnitude by peptide pool. The panels show individual background-subtracted IFN-γ ELISPOT counts to each antigen at 2 weeks post the second vaccination for each peptide pool and by each vaccine group. The horizontal lines indicate median values for each group. SFC  =  spot forming cells.

The median magnitude (log-scale) of positive IFN-γ ELISPOT responses across all peptide pools by vaccination group is shown in **Figure 3**. Each group that received Ad35-GRIN/ENV had a median response magnitude to any antigen ranging from 84 to 114 SFC/10^6^ PBMC, with some significant differences between the three dosage levels. The median SFC/10^6^ PBMC to any antigen was 78–139 across Groups A–C and 158–174 in Group D after each of the vaccinations (**Table S3**). Responses were seen across all proteins with ranges from ∼100 SFC to ∼3000 SFC/10^6^ PBMC. Overall, post-vaccination, there was a significant difference in the response among Ad35-GRIN recipients (Group D) and Ad35-GRIN/ENV recipients (in Groups B and C) for Gag, Pol/Int and Nef pools (Figure 4). The Gag response in Group D was significantly higher than in Group B (p = 0.001) and Group C (p = 0.007). In addition, Pol/Int response in Group D was significantly higher than in Group B (p = 0.0001), and Nef response in Group D was significantly higher than in Group B (p = 0.001), and Group C (p = 0.001). Responses of high magnitude to Gag, RT and Pol/Int were seen in several individuals from multiple groups at the peak of the response (2 or 4 weeks post-second vaccine), but the magnitude of IFN-γ ELISPOT responses to Nef and Env were generally lower (**Figure 4** **and** **Table S3**). There did not appear to be any pattern across the groups with regard to the frequency of responses to individual antigens, indicating no particular immunodominance. However, in Group D, the balance of responses to the four antigens was similar at 2 weeks post-second vaccine; 86% response rate to Gag, 71% for RT and Pol/Int and 57% to Nef. Group D also had the highest Nef response rate compared with Groups A–C.

### Polychromatic Flow Cytometry

Polychromatic flow cytometry (PFC) assays to characterize the responding T-cell phenotype and assess polyfunctionality were performed on cryopreserved PBMCs at study baseline and 2 weeks after the second vaccination for Groups B–D. For PFC assays, the mean cell recovery for vials of 10^7^ PBMC/mL was 8.2×10^6^ (4.9–16.5) with a mean viability of 94.7% (82.3–99.3%). All samples tested positive for cytokine production when stimulated by SEB. The gating strategy is shown in **Figure S2**. HIV-1 specific CD8+ T-cell responses were more commonly observed than CD4+ T-cell responses, and displayed polyfunctional phenotype with IFN-γ, TNF-α production and CD107 expression dominating the response and with a low frequency of IL-2 production (Tables 4 and 5). No obvious dose response effect on magnitude was observed but, CD8+ responses were greater in Group D than in Groups B or C. The magnitude of both CD4+ and CD8+ cytokine positive T cells were greater post-vaccination than at baseline or in the placebo group (**Tables 4** **and** **5**). The magnitude of the responses detected by PFC and IFN-γ ELISPOT were comparable; individuals with high magnitude responses to Gag, RT and Pol/Int in the IFN-γ ELISPOT also had high PFC responses. For example, there was one individual in Group D with a magnitude of 2.18% Nef-specific CD8+ T cells who also had an IFN-γ ELISPOT response of 1060 SFC/10^6^ at the same timepoint. The magnitude of responses to Nef and Env were generally lower in both the PFC and IFN-γ ELISPOT. CD4+ T-cell response rates to the individual peptide pools were low and sporadic (only 1 or 2 responders to one pool in each group). There were no Env-specific CD8+ T-cell responses observed in Groups B or C, but CD8+ T-cell responses were seen to Gag and RT in Groups B–D (**Table S4**). The numbers of samples available in each group precluded statistical analysis of response rate between groups and protein inserts.

**Table 4:** Polychromatic flow cytometry of Env-specific T cells.

		ENV-specific CD8+ T cells				ENV-specific CD4+ T cells			
		IFN-γ	CD107a	TNF-α	IL-2	IFN-γ	CD107a	TNF-α	IL-2
aBaseline	cMedian	0.023	0.030	0.012	0.027	0.013	0.006	0.012	0.034
	cRange	0–0.224	0–0.100	0–0.099	0–0.115	0–0.150	0–0.028	0–0.358	0–0.313
Positive/tested (%)		2/25 (8.0%)	0/25 (0%)	1/25 (4.0%)	0/25 (0.0%)	3/25 (12.0%)	0/25 (0%)	1/25 (4.0%)	1/25 (4.0%)
bPlacebo	cMedian	0.005	0.028	0.009	0.012	0.023	0.003	0.013	0.026
	cRange	0–0.121	0.008–0.088	0–0.057	0.002–0.127	0–0.099	0.002–0.034	0.003–0.033	0.015–0.125
Positive/tested (%)		0/7 (0%)	0/7 (0%)	0/7 (0%)	0/7 (0.0%)	1/7 (14.3%)	1/7 (14.3%)	0/7 (0%)	0/7 (0.0%)
bGroup B	cMedian	0.065	0.041	0.041	0.030	0.042	0.008	0.072	0.072
	cRange	0–0.176	0–0.156	0.010–0.197	0.014–0.079	0.017–0.095	0–0.034	0.029–0.157	0.022–0.149
Positive/tested (%)		1/8 (12.5%)	1/8 (12.5%)	1/8 (12.5%)	0/8 (0.0%)	1/8 (12.5%)	0/8 (0%)	2/8 (25.0%)	3/8 (37.5%)
bGroup C	cMedian	0.050	0.035	0.048	0.047	0.061	0.010	0.047	0.052
	cRange	0.026–0.157	0.008–0.273	0.025–0.165	0.017–0.093	0.020–0.264	0–0.032	0.015–0.780	0.032–0.205
Positive/tested (%)		1/9 (11.1%)	1/9 (11.1%)	1/9 (11.1%)	0/9 (0.0%)	1/9 (11.1%)	1/9 (11.1%)	1/9 (11.1%)	1/9 (11.1%)

a All samples at baseline,

b Samples at 2 weeks post second vaccination,

c Frequency of positive cells.

**Table 5:** Polychromatic flow cytometry of GRIN-specific T cells.

		GRIN-specific CD8+ T cells				GRIN-specific CD4+ T cells			
		IFN-γ	CD107a	TNF-α	IL-2	IFN-γ	CD107a	TNF-α	IL-2
Baseline	cMedian	0.087	0.083	0.073	0.077	0.043	0.022	0.064	0.086
	cRange	0–0.304	0–0.541	0–0.543	0.010–0.640	0–0.265	0–0.091	0.009–0.360	0.027–0.590
Positive/tested (%)		2/36 (5.6%)	1/36 (2.8%)	1/36 (2.8%)	0/36 (0.0%)	0/36 (0.0%)	2/36 (5.6%)	0/36 (0.0%)	0/36 (0.0%)
Placebo	cMedian	0.077	0.076	0.103	0.165	0.040	0.025	0.082	0.191
	cRange	0.010–0.380	0.024–0.208	0–0.860	0.015–1.330	0–0.296	0.005–0.087	0.034–0.780	0.042–1.220
Positive/tested (%)		0/11 (0%)	0/11 (0%)	0/11 (0%)	0/11 (0%)	0/11 (0.0%)	1/11 (9.1%)	0/11 (0.0%)	0/11 (0.0%)
Group B	cMedian	0.247	0.277	0.158	0.156	0.127	0.018	0.111	0.160
	cRange	0.068–2.142	0.079–1.357	0.079–1.911	0.065–0.671	0.052–0.256	0–0.044	0.029–0.203	0.085–0.245
Positive/tested (%)		4/8 (50.0%)	4/8 (50.0%)	3/8 (37.5%)	2/8 (25.0%)	1/8 (12.5%)	0/8 (0.0%)	3/8 (37.5%)	3/8 (37.5%)
Group C	cMedian	0.280	0.109	0.160	0.131	0.105	0.019	0.092	0.128
	cRange	0.060–2.710	0.059–2.670	0.030–2.836	0.034–0.331	0.007–0.491	0.004–0.062	0.026–0.324	0.042–0.219
Positive/tested (%)		4/9 (44.4%)	3/9 (33.3%)	3/9 (33.3%)	4/9 (44.4%)	1/9 (11.1%)	1/9 (11.1%)	1/9 (11.1%)	0/9 (0.0%)
Group D	cMedian	0.564	0.341	0.408	0.236	0.129	0.012	0.208	0.191
	cRange	0.027–3.361	0.022–1.370	0.051–3.019	0.121–0.529	0.048–0.318	(0.010–0.033)	0.127–0.372	0.166–0.370
Positive/tested (%)		6/7 (85.7%)	6/7 (85.7%)	6/7 (85.7%)	3/7 (42.9%)	3/7 (42.9%)	0/7 (0%)	5/7 (71.4%)	1/7 (14.3%)

a All samples at baseline,

b Samples at 2 weeks post second vaccination,

c Frequency of positive cells.

### HIV-specific Antibody Responses

**Figure 5:**
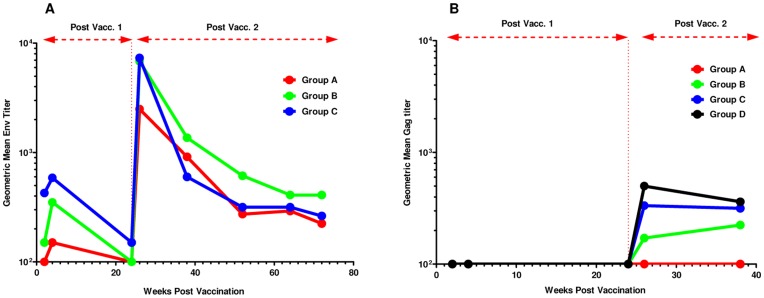
Magnitude of HIV-specific antibodies. The geometric mean of a) anti-ENV Subtype A (UG037)- and b) anti-p24 (IIIB)-specific antibody titers is shown at baseline, at 4 and 24 weeks post-first vaccination, and at 2, 14, 28, 40 and 48 weeks post-second vaccination.

HIV-specific IgG antibody responses to a subtype A UG037 gp140 protein were measured at baseline, 4 and 24 weeks after the first vaccination and 2, 4, 14, 28, 40 and 48 weeks after the second vaccination. One of 12 subjects (8%) who received placebo had a positive response at all time points including baseline, while none of the subjects in Groups A–C (0/9, 0/8, 0/9, respectively) had positive responses at baseline. The majority (80–100%) of vaccinated volunteers in Groups A–C had a positive IgG gp140 ELISA at 4 weeks post-first immunization while all had positive responses at two weeks post-second immunization **(** **Figure 5** **,** **Table S5**) as defined by a titer greater than or equal to 100 at the assay cut-off. The positive response rate at both 4 weeks post-first vaccination and 2 weeks post-second vaccination was significantly higher in the vaccine groups compared to placebo (p<0.0001). Geometric mean titers (and range) were calculated among those responders with quantifiable titers. There was a significant rise in Env antibody titer post-second vaccination in the vaccine groups (signed rank test; p<0.0001), based on the log titer difference between baseline and 2 weeks post-second vaccine. Antibody titers fell significantly from 4 weeks post first vaccination to prior to the second vaccination (p = 0.0002) and going from 2 weeks to 14 weeks after the second vaccination (p<0.0001). Antibody titers peaked 2 weeks after the second vaccination and slowly decreased over time. There was no significant difference in the distribution of titers (p = 0.07) at 2 weeks post-second vaccination among the vaccine groups.

Subtype B Gag p24-specific IgG antibody responses were also measured at baseline, 2, 4 and 24 weeks after the first vaccination and 2 and 14 weeks after the second vaccination. Such antibodies were detected in none of the vaccinated individuals at 4 weeks post-first immunization and in 0%, 38%, 89% and 71% respectively of vaccine recipients from Groups A, B, C and D at 2 weeks after the second vaccination as defined by a titer greater than or equal to 100 at the assay cut-off. (**Figure 5**, **Table S5**). Geometric mean titers (and range) were calculated among those responders with quantifiable titers. The positive response rate at 2 weeks post-second vaccine was significantly higher in Groups B, C and D compared to Group A and placebo (p<0.0001). There was a significant rise in antibody titer post-second vaccination in Groups B, C and D (signed rank test; p = 0.001), based on the log titer difference between 2 weeks post first vaccination and 4 weeks post-second vaccination. In Groups B, C and D, the antibody titer did not significantly change going from 2 to 14 weeks post-second vaccination (p = 0.63). There was no significant difference in the distribution of titers (p = 0.08) at 2 weeks post-second vaccination between Groups B, C and D.

### HIV Neutralizing Antibody Response

None of the samples had neutralizing titers above the cut-off of the assay against any of the 9 viruses tested (data not shown).

### Ad35-specific Neutralizing Antibody Response

Samples were tested for the presence of Ad35-specific neutralizing antibodies at baseline, 4 weeks post-first vaccination and 2 weeks post-second vaccination. Responses were seen in only 10–30% of participants in Groups A–D after the first vaccination and were generally of low titer; the median titer among those that scored positive in the assay at that time was 56, 171, 32 and 40 in the four groups, respectively. (**Table S5, Figure S3**). However, Ad35 neutralization was observed more frequently after the second vaccination, with a dose-dependent trend towards an increased response rate, with 33%, 38%, 89% of individuals detected with Ad35-specific neutralizing antibodies observed respectively, for Groups A–C (p = 0.08). Nevertheless, the geometric mean titers among positive volunteers remained relatively modest and were not significantly different among the vaccination groups with geometric mean titers of 406, 31, 57 and 81 in Groups A–D, respectively. All placebo recipients tested negative at each of the visits (data not shown). As reference, the median Ad35 neutralizing titer among 6 potential study participants who had evidence of previous natural Ad35 infection at study screening was 47.5 (range: 25–858). These subjects were not enrolled into the study per protocol exclusion criteria (data not shown).

### HIV Serology at the End of the Study

At their last study visit, all 48 volunteers who remained in follow-up (15 from the placebo group, 8 from Group A, 8 from Group B, 9 from Group C, and 8 from Group D) were tested for HIV. All volunteers tested negative for Abbott m2000 Real Time PCR HIV-1 RNA. All placebo recipients and Group D volunteers tested negative by ELISA (Bio-Rad Genetic Systems HIV 1/2 Plus O EIA and Abbott Architect HIV Ab/Ag Combo EIA) and Bio-Rad Genetic Systems HIV-1 Western Blot. In Groups A, B, and C, respectively 25%, 38%, 78% of the volunteers tested positive with HIV 1/2 Plus O EIA and 50%, 100% and 56% with Abbott Architect HIV Ab/Ag Combo EIA.

## Discussion

This study shows that Ad35-GRIN/ENV is safe and immunogenic when administered twice to Ad35-seronegative healthy volunteers with doses from 2×10^9^ to 2×10^11^ vp. Reactogenicity was dose-related with frequent moderate to severe systemic symptoms consisting of headache, malaise and chills that began within 12–24 hours of initial vaccination. All reactions were self-limited and resolved within hours (systemic symptoms) to a few days (injection site symptoms).

Ad35-GRIN/ENV at all dose levels was found to be immunogenic, producing IFN-γ ELISPOT responses in 90% of all vaccinated individuals although the magnitude was relatively modest to individual peptide pools. Anti-gp140 binding antibodies were seen in all vaccine recipients in Groups A–C. The median and range of Env and Nef ELISPOT responses were lower than those seen to Gag, RT and Int and likely because the PFC assay is less sensitive than the ELISPOT assay, we saw fewer responses to Env and Nef. The IAVI-HIL ELISPOT assay has a positive cut off value of 38 SFC per million PBMC. Assuming 50% of PBMC are T cells, the 38 SFC value would equate to 0.0076% of CD3+ T cells producing IFN-γ. The median range of Env ELISPOT was 60–108 SFC per million PBMC across groups A–C which equates to 0.012 to 0.0216% of CD3+ T cells responding. Such values are below the limit of detection of our current ICS assay. Because of the lower sensitivity of the PFC assays, responses at 2 weeks post-2^nd^ vaccination were less frequently positive than the IFN-γ ELISPOT assays, but indicated that the cellular response was primarily mediated by CD8+ T lymphocytes and was typically polyfunctional, with TNF-α and CD107 detected in addition to IFN-γ. A trend of a dose-response effect in the proportion of subjects with positive IFN-γ ELISPOT assays was seen after a single injection (the small size of the study groups precluded an assessment of statistical significance); however, the booster vaccination at 6 months induced responses in nearly all the participants in all groups. There was no clear indication of an increase in magnitude or breadth (the latter by the number of peptide pools recognized) in IFN-γ ELISPOT responses by dose or as a result of the 6-month booster immunization.

HIV gp140-specific binding antibodies were frequently seen after the first immunization among recipients of the Ad35-GRIN/ENV with a titer significantly increased with the 6-month boost in all groups (p<0.0004), but declined appreciably within the following 6 months. In addition, anti-Ad35 neutralizing antibodies were induced at a low frequency and low titer after the first immunization, but the 6-month boost increased the proportion of responders significantly, particularly among those who received the 2×10^11^ vp dose.

The presence of pre-existing Ad neutralizing antibodies and the impact of such antibodies on reducing HIV-specific immune responses after multiple immunizations remains a major concern for the use of Ad vectors. In this study where volunteers enrolled were all Ad35-specific neutralizing antibody negative at baseline, we found that even after a second immunization with Ad35 vectors, Ad35-specific neutralizing antibody titers remained low, regardless of the dose. HIV Env- and Gag-specific antibodies were significantly boosted after the second immunization with modest increases in cellular responses, suggesting that HIV-specific cellular and humoral responses were not impacted by Ad35-specific neutralizing antibodies induced by the first Ad35 vector immunization. We cannot however exclude the interference of cross-reactive preexisting Ad-specific T-cell immune responses on the vaccine-induced T-cell responses [48]. The low prevalence and titers of Ad35 compared with Ad5 neutralizing antibodies as well as the induction of adequate cellular and humoral responses shown in this study supports the use of Ad35 vectors in heterologous or homologous prime-boost strategies [49].

This study also explored the potential phenomenon of an Env immunodominance as suggested in animals [50], [51], [52] and other human studies [53], [54] by comparing IFN-γ ELISPOT responses with GRIN peptide pools between Group B and Group D (who received 2×10^10^ vp of Ad35-GRIN/ENV or 1×10^10^ vp of Ad35-GRIN, respectively). Although the small size of the groups precluded firm conclusions, the frequency of positive IFN-γ ELISPOT assays in Group D appeared higher after the first injection relative to Group B. Furthermore, overall, the magnitude of positive responses was significantly higher in Group D (vaccinated in the absence of Env genes) compared to Group B for Gag, Pol/Int and Nef pools. There was insufficient evidence from this small study to evaluate the mechanism of possible Env interference, however as indicated above with the low Ad35 neutralization titers even after the second boost and at the highest dose, we could not see an impact on insert specific immune responses. Understanding how best to administer antigens to avoid immunodominance, competition and the effects of pre-existing or post immunization vector specific responses is an ongoing issue for development of HIV, malaria, Cancer, TB and other vaccines [55], [56], [57], [58], [59].

Interestingly, one study participant who received placebo in this trial demonstrated IFN-γ ELISPOT responses and anti-gp140 binding antibody at baseline and all timepoints throughout the trial in spite of testing negative in the licensed diagnostic kit Abbott Architect HIV Ab/Ag Combo EIA at the University of Rochester at study entry and negative HIV RNA levels. This person did not disclose any behavior associated with high-risk for HIV infection and had not participated in previous HIV vaccine clinical trials. The reasons for these findings are still under investigation.

This study adds to the accumulating literature of safe and immunogenic candidate vaccines that could be included in combination vaccine regimens. Similar to the Ad5 vector tested in the Step Study, the Ad35 vector induced a high frequency of HIV-specific CD8+ T-cell responses, however, our data suggests that the Ad35-GRIN/ENV candidate induced a broader response in terms of the number of protein regions recognized. In the original Step study, 218 of 354 (62%) of individuals recognized two to three HIV proteins with a median of 1 protein per person [26], [27]. Further assessment of the breadth of response was performed in 72 subjects who participated in an earlier Phase I trial of the same vaccine utilized in the Step study. In the latter study, using mini-pools containing eight 9-amino acid (aa) peptides spanning 16 aa of the vaccine sequence, the median (and mean) of positive mini-pool responses per subject was 1 (1) for Gag, 1 (1) for Nef and 2 (3) for Pol [60]. It is unclear what this may mean in terms of potential vaccine efficacy, but the Step Study did show evidence that vaccine-elicited T cells had an impact on the HIV-1 strains that established infection (a sieve effect) [61] and thus it is reasonable to conclude that a broadened antiviral effect could be beneficial. In addition, this study suggests that improved non-envelope IFN-γ ELISPOT responses may be induced if vaccination with Env proteins or Env encoding vectors are separated in time or space.

As noted above, the community-based trial conducted in Thailand (RV144) that employed a combination regimen of a recombinant canarypox vector expressing HIV-1 *gag, protease, env* genes boosted by a recombinant HIV-1 gp120 envelope protein subunit showed protection against HIV acquisition however without a measurable effect on viremia or the CD4+ T-cell count in vaccinated and infected subjects. The vaccination regimen induced modest levels of HIV-specific cellular immune responses, mediated primarily by CD4+ T cells and high titers of HIV-envelope specific binding antibodies [32]. The Step and RV144 results reinforce the need to develop new vaccines or vaccine combinations able to induce more effective immune responses, in particular at the mucosal level where HIV transmission events occur [3]. Combination regimens using heterologous vectors in prime-boost and inserts aiming at broadening CD4+ and CD8+ T-cell responses such as mosaics [62] and conserved sequences [63] are promising avenues. Indeed, three other phase I clinical trials, one with Ad35-GRIN in combination with an adjuvanted core HIV protein vaccine (IAVI B002; NCT01264445), a second with Ad35-ENV in combination with Ad26-ENVA (IAVI B003-IPCAVD004-HVTN091; NCT01215149), both of which have completed vaccinations, and a third with Ad35-GRIN/ENV in prime-boost regimens with DNA administered by electroporation and adjuvanted with IL-12 have been initiated (IAVI B004; NCT01496989). These trials are designed to increase the breadth in terms of epitope coverage, improving the CD4+ and CD8+ T-cell responses and functional antibody responses. In spite of the lack of HIV neutralizing antibodies, additional studies on the fine specificity of the Env antibody response induced by Ad35-GRIN/ENV are also warranted following the recent findings that IgG binding antibody to a scaffolded HIV-1 gp120 V1V2 protein encompassing the gut homing marker α4β7 integrin binding site was identified in a *post-hoc* analysis as a correlate of risk for acquisition of HIV infection in RV144 [64]. In conclusion, the Ad35-GRIN/ENV candidate was generally well tolerated and immunogenic at a dose of 2×10^10^ vp and has moved forward into additional studies to further assess safety and immunogenicity and should also be considered as a potential component of a regimen tested in efficacy trials.

## Supporting Information

Figure S1 **Breadth of response by IFN-γ ELISPOT assay.** The numbers inside the stacked bars represent the percent of volunteers responding to 1, 2, 3, 4 or 5 different HIV-peptide pools (Gag, RT, Pol/Int, Nef and Env) as indicated on the first row of the X-axis. The second row of the X-axis shows the time point examined. The colors represent data from Groups A–D, respectively. The inserted table shows the number of volunteers per group at each time point that contributed ELISPOT data for the bar graph.
(TIF)

Figure S2 **Flow gating strategy.** **A.** Quality control gating. A time vs. CD4 QD605 is first applied to ensure acquisition of data occurred without blockages and in this example to remove micro aggregates formed by the CD4 QD605 antibody. Following this a FSC-H vs. FSC-A gate is applied in order to exclude doublets and cell clumps. Once the lymphocyte population is selected a dump gate is applied to ensure that non-viable cells as well as B cells and monocytes are excluded from analysis. A generous CD3 vs. cytokine gate is applied to include any down-regulated antigen specific cells. The example shown here is for IFN-γ but all cytokines are evaluated. **B.** CD4, CD8 and cytokine gating. The CD4 and CD8 gates are applied in a similar manner, generous CD4 and CD8 gates are applied vs. cytokine and contaminating cells are removed subsequently by more stringent gating. Each cytokine is gated vs. the opposite lineage and polyfunctional responses are assessed using the Boolean function of FlowJo.
(TIF)

Figure S3 **Ad35-specific neutralizing antibody titers pre-vaccination, at 4 weeks post-first and 2 weeks post-second vaccination.** Each dot in the scatter plot represents an individual Ad35 neutralization titer. EC90 titers below the assay cut-off are plotted at the cutoff value of 16. At each time point for each of the vaccine groups, the middle horizontal bar shows the median value and the horizontal bars to the top and bottom of the median represent the 75% and 25% quartile values.
(TIF)

Tables S1 Frequency of local reactions per maximum severity assessment.
(DOCX)

Table S2 Frequency of systemic reactions per maximum severity assessment.
(DOCX)

Table S3 Median and range of positive IFN-γ ELISPOT responses (SFC/10^6^ PBMC) across all visits.
(DOCX)

Table S4 CD4 and CD8 positive response rates to any antigen by polychromatic flow cytometry.
(DOCX)

Table S5 Summary of antibody response frequencies.
(DOCX)

Protocol S1 **Trial Protocol.**
(PDF)

Checklist S1 **CONSORT Checklist.**
(DOC)
