# Supplementary figures and images for: A Phase I Double Blind, Placebo-Controlled, Randomized Study of a Multigenic HIV-1 Adenovirus Subtype 35 Vector Vaccine in Healthy Uninfected Adults
Source: PLoS One. 2012 Aug 3;7(8):e41936. doi: 10.1371/journal.pone.0041936 (PMC3411704; doi:10.1371/journal.pone.0041936)

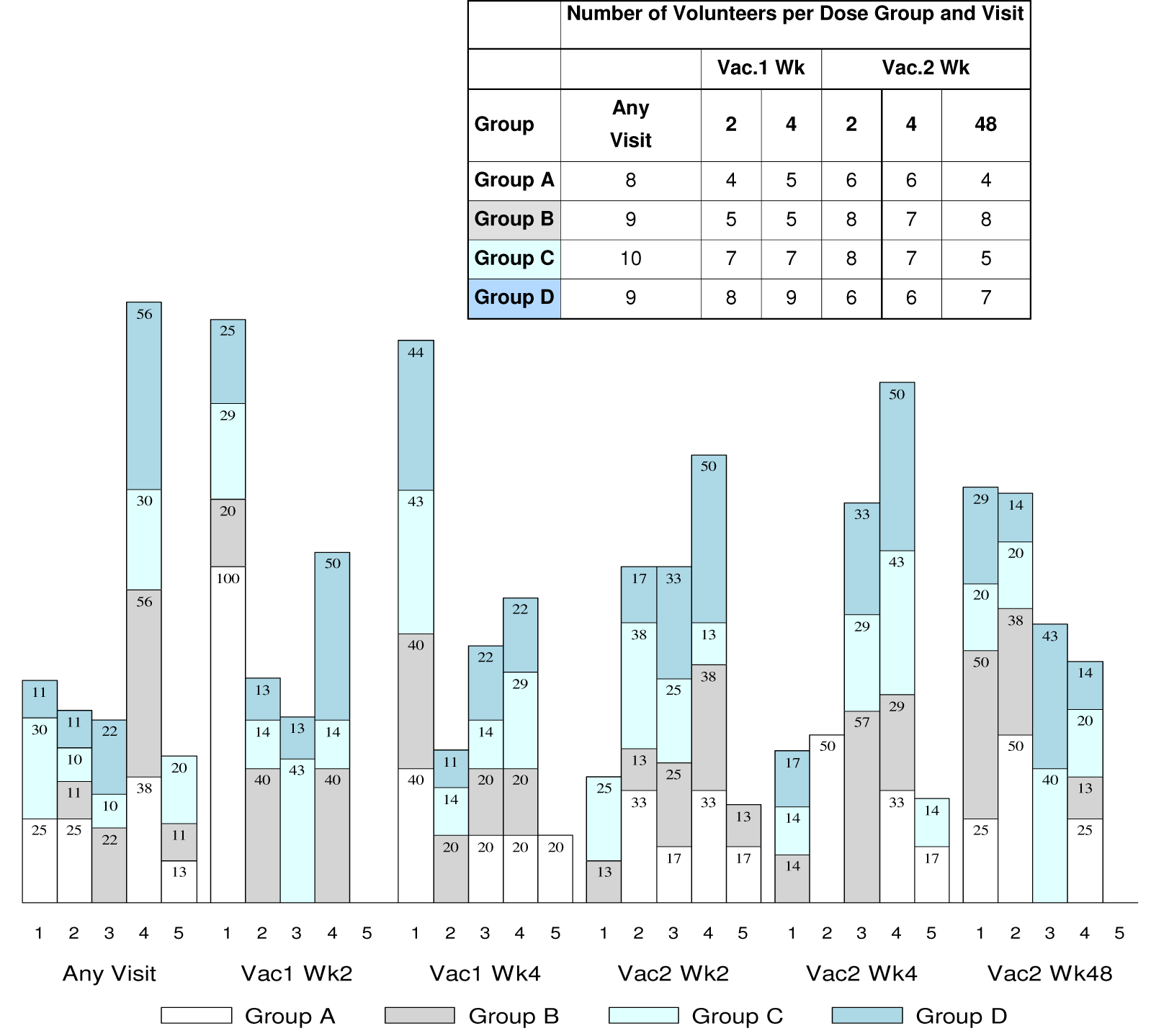

Supplement: Figure S1 — Breadth of response by IFN-γ ELISPOT assay. The numbers inside the stacked bars represent the percent of volunteers responding to 1, 2, 3, 4 or 5 different HIV-peptide pools (Gag, RT, Pol/Int, Nef and Env) as indicated on the first row of the X-axis. The second row of the X-axis shows the time point examined. The colors represent data from Groups A–D, respectively. The inserted table shows the number of volunteers per group at each time point that contributed ELISPOT data for the bar graph. (TIF) [file pone.0041936.s001.tif]

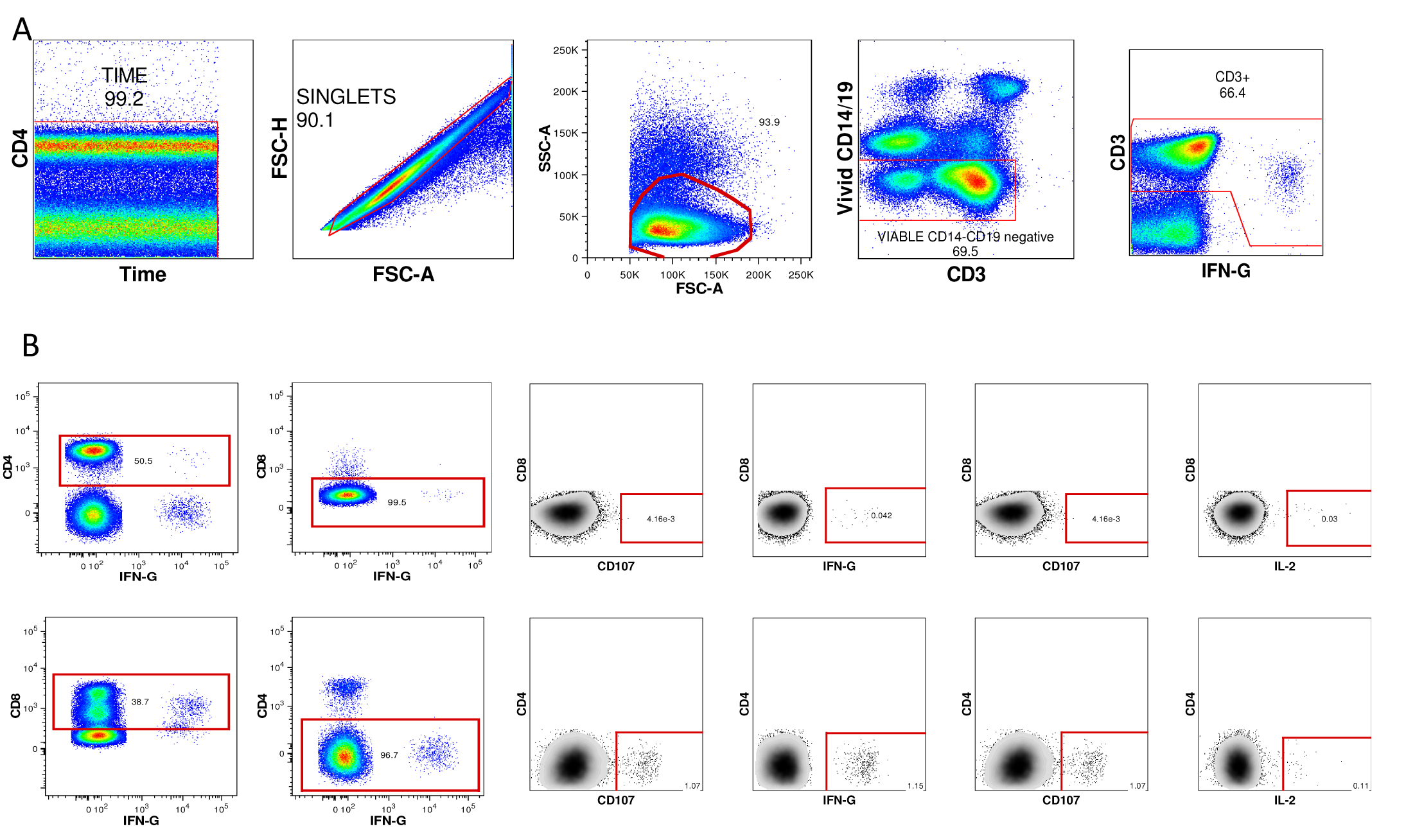

Supplement: Figure S2 — Flow gating strategy. A. Quality control gating. A time vs. CD4 QD605 is first applied to ensure acquisition of data occurred without blockages and in this example to remove micro aggregates formed by the CD4 QD605 antibody. Following this a FSC-H vs. FSC-A gate is applied in order to exclude doublets and cell clumps. Once the lymphocyte population is selected a dump gate is applied to ensure that non-viable cells as well as B cells and monocytes are excluded from analysis. A generous CD3 vs. cytokine gate is applied to include any down-regulated antigen specific cells. The example shown here is for IFN-γ but all cytokines are evaluated. B. CD4, CD8 and cytokine gating. The CD4 and CD8 gates are applied in a similar manner, generous CD4 and CD8 gates are applied vs. cytokine and contaminating cells are removed subsequently by more stringent gating. Each cytokine is gated vs. the opposite lineage and polyfunctional responses are assessed using the Boolean function of FlowJo. (TIF) [file pone.0041936.s002.tif]

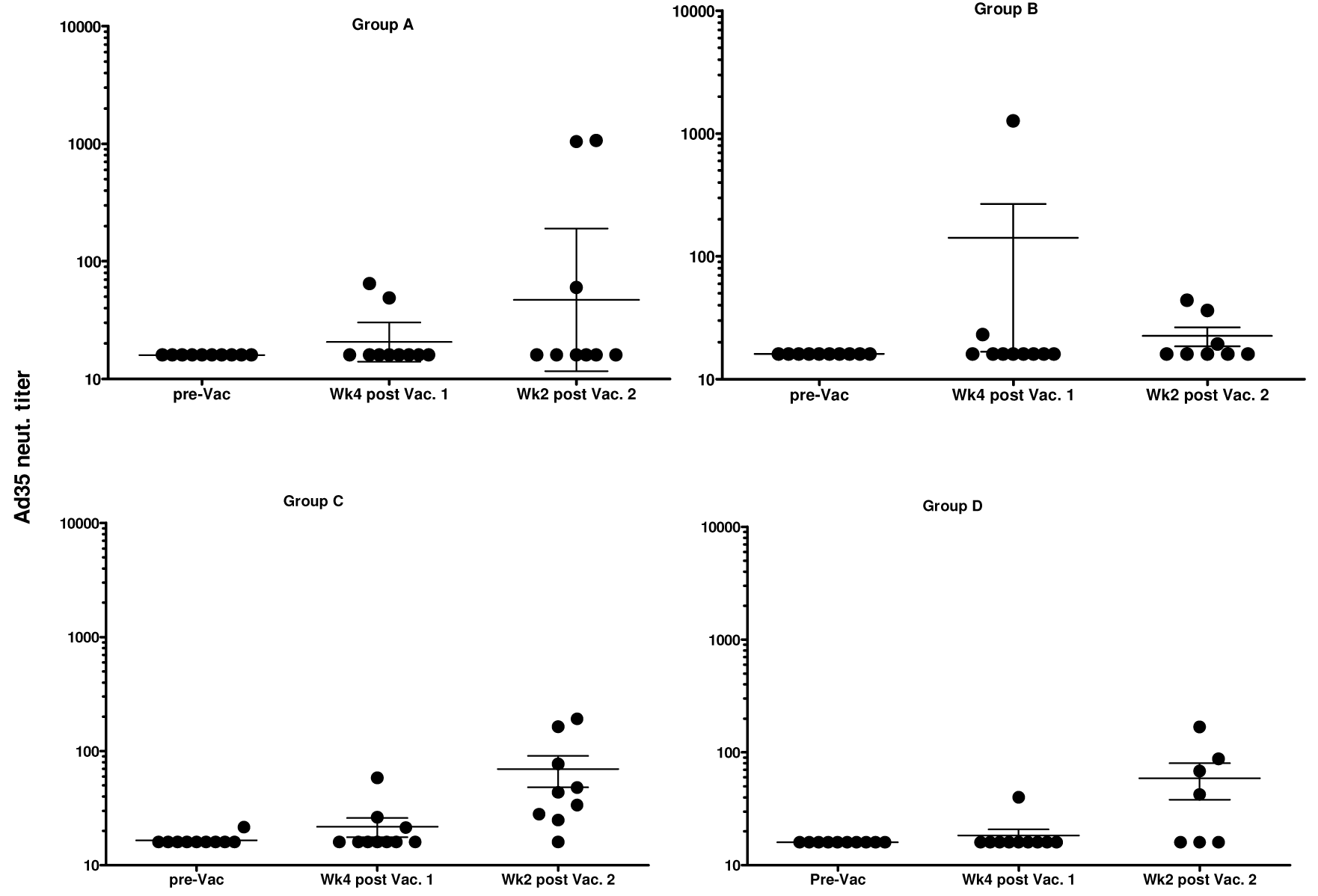

Supplement: Figure S3 — Ad35-specific neutralizing antibody titers pre-vaccination, at 4 weeks post-first and 2 weeks post-second vaccination. Each dot in the scatter plot represents an individual Ad35 neutralization titer. EC90 titers below the assay cut-off are plotted at the cutoff value of 16. At each time point for each of the vaccine groups, the middle horizontal bar shows the median value and the horizontal bars to the top and bottom of the median represent the 75% and 25% quartile values. (TIF) [file pone.0041936.s003.tif]
