# Supplementary material for: A Phase I Double Blind, Placebo-Controlled, Randomized Study of a Multigenic HIV-1 Adenovirus Subtype 35 Vector Vaccine in Healthy Uninfected Adults
Source: PLoS One. 2012 Aug 3;7(8):e41936. doi: 10.1371/journal.pone.0041936 (PMC3411704; doi:10.1371/journal.pone.0041936)
Supplement: Tables S1 — Frequency of local reactions per maximum severity assessment. (DOCX) [file pone.0041936.s004.docx]

**Table S1. Frequency of Local Reactions per Maximum Severity Assessment.**

|  | | Placebo | | | Ad35-GRIN/ENV (2x10^9^ vp) | | | Ad35-GRIN/ENV (2x10^10^ vp) | | | Ad35-GRIN/ENV (2x10^11^ vp) | | | Ad35-GRIN (1x10^10^ vp) | | |
| --- | --- | --- | --- | --- | --- | --- | --- | --- | --- | --- | --- | --- | --- | --- | --- | --- |
|  | | Vac1 | Vac2 | Cumulative | Vac1 | Vac2 | Cumulative | Vac1 | Vac2 | Cumulative | Vac1 | Vac2 | Cumulative | Vac1 | Vac2 | Cumulative |
|  | | N=16 | N=16 | N=16 | N=10 | N=9 | N=10 | N=10 | N=8 | N=10 | N=10 | N=9 | N=10 | N=10 | N=8 | N=10 |
| Maximum Reaction | Mild | 7 (43.8%) | 6 (37.5%) | 10 (62.5%) | 5 (50.0%) | 4 (44.4%) | 5 (50.0%) | 8 (80.0%) | 4 (50.0%) | 5 (50.0%) | 4 (40.0%) | 4 (44.4%) | 3 (30.0%) | 9 (90.0%) | 6 (75.0%) | 7 (70.0%) |
|  | Moderate | 1 (6.3%) | 2 (12.5%) | 3 (18.8%) | 1 (10.0%) | 2 (22.2%) | 2 (20.0%) | 1 (10.0%) | 3 (37.5%) | 4 (40.0%) | 4 (40.0%) | 3 (33.3%) | 4 (40.0%) | 1 (10.0%) | 2 (25.0%) | 3 (30.0%) |
|  | Severe | 0 | 0 | 0 | 0 | 1 (11.1%) | 1 (10.0%) | 0 | 1 (12.5%) | 1 (10.0%) | 2 (20.0%) | 2 (22.2%) | 3 (30.0%) | 0 | 0 | 0 |
| Pain | Mild | 5 (31.3%) | 5 (31.3%) | 9 (56.3%) | 2 (20.0%) | 5 (55.6%) | 5 (50.0%) | 7 (70.0%) | 4 (50.0%) | 6 (60.0%) | 6 (60.0%) | 3 (33.3%) | 6 (60.0%) | 9 (90.0%) | 6 (75.0%) | 9 (90.0%) |
|  | Moderate | 1 (6.3%) | 1 (6.3%) | 2 (12.5%) | 1 (10.0%) | 0 | 0 | 1 (10.0%) | 1 (12.5%) | 2 (20.0%) | 1 (10.0%) | 3 (33.3%) | 2 (20.0%) | 1 (10.0%) | 0 | 1 (10.0%) |
|  | Severe | 0 | 0 | 0 | 0 | 1 (11.1%) | 1 (10.0%) | 0 | 1 (12.5%) | 1 (10.0%) | 2 (20.0%) | 0 | 2 (20.0%) | 0 | 0 | 0 |
| Tenderness | Mild | 6 (37.5%) | 6 (37.5%) | 9 (56.3%) | 4 (40.0%) | 4 (44.4%) | 5 (50.0%) | 8 (80.0%) | 5 (62.5%) | 6 (60.0%) | 4 (40.0%) | 5 (55.6%) | 3 (30.0%) | 7 (70.0%) | 6 (75.0%) | 6 (60.0%) |
|  | Moderate | 0 | 1 (6.3%) | 1 (6.3%) | 0 | 2 (22.2%) | 2 (20.0%) | 1 (10.0%) | 3 (37.5%) | 4 (40.0%) | 4 (40.0%) | 2 (22.2%) | 4 (40.0%) | 1 (10.0%) | 2 (25.0%) | 3 (30.0%) |
|  | Severe | 0 | 0 | 0 | 0 | 1 (11.1%) | 1 (10.0%) | 0 | 0 | 0 | 2 (20.0%) | 2 (22.2%) | 3 (30.0%) | 0 | 0 | 0 |
| Erythema* | Present | 1 (6.3%) | 0 | 1 (6.3%) | 0 | 2 (22.2%) | 2 (20.0%) | 0 | 1 (12.5%) | 1 (10.0%) | 1 (10.0%) | 0 | 1 (10.0%) | 1 (10.0%) | 1 (12.5%) | 0 |
|  | Mild | 0 | 0 | 0 | 0 | 0 | 0 | 0 | 2 (25.0%) | 2 (20.0%) | 0 | 0 | 0 | 1 (10.0%) | 1 (12.5%) | 2 (20.0%) |
|  | Moderate | 0 | 0 | 0 | 0 | 0 | 0 | 0 | 1 (12.5%) | 1 (10.0%) | 0 | 2 (22.2%) | 2 (20.0%) | 0 | 0 | 0 |
| Induration | Present | 0 | 0 | 0 | 0 | 1 (11.1%) | 1 (10.0%) | 0 | 0 | 0 | 0 | 0 | 0 | 0 | 0 | 0 |
| Crust or Scab | Present | 0 | 0 | 0 | 0 | 0 | 0 | 0 | 1 (12.5%) | 1 (10.0%) | 0 | 0 | 0 | 1 (10.0%) | 0 | 1 (10.0%) |

Vac1: First vaccination at Day 0

Vac2: Second Vaccination at Month 6

Present: For erythema/skin discoloration* and induration, a reaction is considered ‘present’ if the corresponding measurement is less than the criteria for mild. For formation of crust or scab, any measured reaction is considered ‘present’ as there is no toxicity table grading used for this reaction.
