# Supplementary material for: A Phase I Double Blind, Placebo-Controlled, Randomized Study of a Multigenic HIV-1 Adenovirus Subtype 35 Vector Vaccine in Healthy Uninfected Adults
Source: PLoS One. 2012 Aug 3;7(8):e41936. doi: 10.1371/journal.pone.0041936 (PMC3411704; doi:10.1371/journal.pone.0041936)
Supplement: Table S2 — Frequency of systemic reactions per maximum severity assessment. (DOCX) [file pone.0041936.s005.docx]

**Table S2: Frequency of Systemic Reactions per Maximum Severity Assessment**

|  | | Placebo | | | Ad35-GRIN/ENV (2x10^9^ vp) | | | Ad35-GRIN/ENV (2x10^10^ vp) | | | Ad35-GRIN/ENV (2x10^11^ vp) | | | Ad35-GRIN (1x10^10^ vp) | | |
| --- | --- | --- | --- | --- | --- | --- | --- | --- | --- | --- | --- | --- | --- | --- | --- | --- |
|  | | Vac1 | Vac2 | Cumulative | Vac1 | Vac2 | Cumulative | Vac1 | Vac2 | Cumulative | Vac1 | Vac2 | Cumulative | Vac1 | Vac2 | Cumulative |
|  | | N=16 | N=16 | N=16 | N=10 | N=9 | N=10 | N=10 | N=8 | N=10 | N=10 | N=9 | N=10 | N=10 | N=8 | N=10 |
| Maximum Reaction | Mild | 8 (50.0%) | 4 (25.0%) | 5 (31.3%) | 3 (30.0%) | 4 (44.4%) | 4 (40.0%) | 3 (30.0%) | 4 (50.0%) | 5 (50.0%) | 2 (20.0%) | 2 (22.2%) | 1 (10.0%) | 3 (30.0%) | 5 (62.5%) | 5 (50.0%) |
|  | Moderate | 2 (12.5%) | 3 (18.8%) | 5 (31.3%) | 2 (20.0%) | 0 | 1 (10.0%) | 2 (20.0%) | 0 | 2 (20.0%) | 2 (20.0%) | 5 (55.6%) | 4 (40.0%) | 3 (30.0%) | 1 (12.5%) | 3 (30.0%) |
|  | Severe | 1 (6.3%) | 0 | 1 (6.3%) | 0 | 1 (11.1%) | 1 (10.0%) | 0 | 0 | 0 | 5 (50.0%) | 0 | 5 (50.0%) | 0 | 0 | 0 |
| Chills | Mild | 2 (12.5%) | 3 (18.8%) | 4 (25.0%) | 1 (10.0%) | 0 | 1 (10.0%) | 1 (10.0%) | 2 (25.0%) | 3 (30.0%) | 3 (30.0%) | 0 | 3 (30.0%) | 0 | 0 | 0 |
|  | Moderate | 0 | 0 | 0 | 0 | 0 | 0 | 0 | 0 | 0 | 1 (10.0%) | 3 (33.3%) | 3 (30.0%) | 1 (10.0%) | 0 | 1 (10.0%) |
|  | Severe | 0 | 0 | 0 | 0 | 0 | 0 | 0 | 0 | 0 | 3 (30.0%) | 0 | 3 (30.0%) | 0 | 0 | 0 |
| Malaise | Mild | 7 (43.8%) | 3 (18.8%) | 6 (37.5%) | 1 (10.0%) | 0 | 0 | 2 (20.0%) | 3 (37.5%) | 4 (40.0%) | 2 (20.0%) | 3 (33.3%) | 4 (40.0%) | 3 (30.0%) | 2 (25.0%) | 3 (30.0%) |
|  | Moderate | 1 (6.3%) | 1 (6.3%) | 2 (12.5%) | 1 (10.0%) | 0 | 1 (10.0%) | 0 | 0 | 0 | 3 (30.0%) | 3 (33.3%) | 3 (30.0%) | 2 (20.0%) | 1 (12.5%) | 3 (30.0%) |
|  | Severe | 0 | 0 | 0 | 0 | 1 (11.1%) | 1 (10.0%) | 0 | 0 | 0 | 3 (30.0%) | 0 | 3 (30.0%) | 0 | 0 | 0 |
| Myalgia | Mild | 2 (12.5%) | 1 (6.3%) | 3 (18.8%) | 1 (10.0%) | 1 (11.1%) | 1 (10.0%) | 2 (20.0%) | 1 (12.5%) | 3 (30.0%) | 2 (20.0%) | 2 (22.2%) | 2 (20.0%) | 4 (40.0%) | 3 (37.5%) | 5 (50.0%) |
|  | Moderate | 0 | 0 | 0 | 1 (10.0%) | 0 | 0 | 0 | 0 | 0 | 1 (10.0%) | 2 (22.2%) | 2 (20.0%) | 0 | 0 | 0 |
|  | Severe | 0 | 0 | 0 | 0 | 1 (11.1%) | 1 (10.0%) | 0 | 0 | 0 | 3 (30.0%) | 0 | 3 (30.0%) | 0 | 0 | 0 |
| Headache | Mild | 6 (37.5%) | 3 (18.8%) | 5 (31.3%) | 4 (40.0%) | 5 (55.6%) | 5 (50.0%) | 2 (20.0%) | 2 (25.0%) | 3 (30.0%) | 4 (40.0%) | 1 (11.1%) | 3 (30.0%) | 2 (20.0%) | 3 (37.5%) | 3 (30.0%) |
|  | Moderate | 1 (6.3%) | 2 (12.5%) | 3 (18.8%) | 1 (10.0%) | 0 | 1 (10.0%) | 1 (10.0%) | 0 | 1 (10.0%) | 3 (30.0%) | 4 (44.4%) | 5 (50.0%) | 1 (10.0%) | 0 | 1 (10.0%) |
|  | Severe | 1 (6.3%) | 0 | 1 (6.3%) | 0 | 0 | 0 | 0 | 0 | 0 | 2 (20.0%) | 0 | 2 (20.0%) | 0 | 0 | 0 |
| Fever, Subjective | Mild | 0 | 2 (12.5%) | 2 (12.5%) | 1 (10.0%) | 0 | 1 (10.0%) | 0 | 0 | 0 | 3 (30.0%) | 1 (11.1%) | 3 (30.0%) | 0 | 0 | 0 |
|  | Moderate | 0 | 0 | 0 | 0 | 0 | 0 | 0 | 0 | 0 | 3 (30.0%) | 0 | 3 (30.0%) | 0 | 0 | 0 |
|  | Severe | 0 | 0 | 0 | 0 | 0 | 0 | 0 | 0 | 0 | 1 (10.0%) | 0 | 1 (10.0%) | 0 | 0 | 0 |
| Max Temperature* | Mild | 1 (6.3%) | 1 (6.3%) | 1 (6.3%) | 0 | 0 | 0 | 2 (20.0%) | 0 | 2 (20.0%) | 4 (40.0%) | 3 (33.3%) | 4 (40.0%) | 0 | 2 (25.0%) | 2 (20.0%) |
|  | Moderate | 0 | 0 | 0 | 0 | 0 | 0 | 0 | 0 | 0 | 2 (20.0%) | 0 | 2 (20.0%) | 0 | 0 | 0 |
|  | Severe | 0 | 0 | 0 | 0 | 0 | 0 | 0 | 0 | 0 | 1 (10.0%) | 0 | 1 (10.0%) | 0 | 0 | 0 |
| Nausea | Mild | 1 (6.3%) | 3 (18.8%) | 2 (12.5%) | 1 (10.0%) | 1 (11.1%) | 2 (20.0%) | 0 | 1 (12.5%) | 1 (10.0%) | 1 (10.0%) | 0 | 1 (10.0%) | 2 (20.0%) | 0 | 2 (20.0%) |
|  | Moderate | 1 (6.3%) | 1 (6.3%) | 2 (12.5%) | 0 | 0 | 0 | 0 | 0 | 0 | 2 (20.0%) | 0 | 2 (20.0%) | 0 | 0 | 0 |
| Vomiting | Mild | 1 (6.3%) | 0 | 1 (6.3%) | 0 | 0 | 0 | 0 | 0 | 0 | 0 | 0 | 0 | 1 (10.0%) | 0 | 1 (10.0%) |
|  | Moderate | 0 | 1 (6.3%) | 1 (6.3%) | 0 | 0 | 0 | 0 | 0 | 0 | 0 | 0 | 0 | 1 (10.0%) | 0 | 1 (10.0%) |
| Arthralgia | Mild | 0 | 0 | 0 | 1 (10.0%) | 0 | 0 | 0 | 0 | 0 | 0 | 0 | 0 | 0 | 0 | 0 |
|  | Moderate | 0 | 0 | 0 | 0 | 0 | 0 | 1 (10.0%) | 0 | 1 (10.0%) | 1 (10.0%) | 0 | 1 (10.0%) | 0 | 0 | 0 |
|  | Severe | 0 | 0 | 0 | 0 | 1 (11.1%) | 1 (10.0%) | 0 | 0 | 0 | 0 | 0 | 0 | 0 | 0 | 0 |

Maximum Temperature* Mild (≥37.7), Moderate (≥38.7), Severe (≥39.4), Very Severe (>40.5)

Vac1: First vaccination at Day 0

Vac2: Second Vaccination at Month 6
