# Supplementary material for: A Phase I Double Blind, Placebo-Controlled, Randomized Study of a Multigenic HIV-1 Adenovirus Subtype 35 Vector Vaccine in Healthy Uninfected Adults
Source: PLoS One. 2012 Aug 3;7(8):e41936. doi: 10.1371/journal.pone.0041936 (PMC3411704; doi:10.1371/journal.pone.0041936)
Supplement: Table S3 — Median and range of positive IFN-γ ELISPOT responses (SFC/106 PBMC) across all visits. (DOCX) [file pone.0041936.s006.docx]

Table S3: Median and Range of Positive IFN-γ ELISPOT Responses (SFC/10^6^ PBMC) across All Visits.

|  |  | Post Vac.1 | | | Post Vac.2 | | |
| --- | --- | --- | --- | --- | --- | --- | --- |
| Dose Group | Pool | N | Median | Min-Max | N | Median | Min-Max |
| Group A | Any GRIN | 24 | 156 | 39 - 836 | 97 | 143 | 39 - 2991 |
|  | Any ENV | 8 | 108 | 58 - 225 | 44 | 86 | 39 - 856 |
|  | Any | 32 | 139 | 39 - 836 | 141 | 109 | 39 - 2991 |
| Group B | Any GRIN | 28 | 91 | 39 - 606 | 112 | 85 | 39 - 1159 |
|  | Any ENV | 14 | 60 | 42 - 159 | 57 | 81 | 40 - 580 |
|  | Any | 42 | 78 | 39 - 606 | 169 | 84 | 39 - 1159 |
| Group C | Any GRIN | 34 | 98 | 39 - 389 | 114 | 106 | 39 - 2793 |
|  | Any ENV | 25 | 99 | 40 - 289 | 66 | 104 | 40 - 701 |
|  | Any | 59 | 99 | 39 - 389 | 180 | 106 | 39 - 2793 |
| Group D | Any GRIN | 59 | 158 | 40 - 528 | 152 | 174 | 39 - 1149 |
|  | Any | 59 | 158 | 40 - 528 | 152 | 174 | 39 - 1149 |
