# Supplementary material for: A Phase I Double Blind, Placebo-Controlled, Randomized Study of a Multigenic HIV-1 Adenovirus Subtype 35 Vector Vaccine in Healthy Uninfected Adults
Source: PLoS One. 2012 Aug 3;7(8):e41936. doi: 10.1371/journal.pone.0041936 (PMC3411704; doi:10.1371/journal.pone.0041936)
Supplement: Table S4 — CD4 and CD8 positive response rates to any antigen by polychromatic flow cytometry. (DOCX) [file pone.0041936.s007.docx]

**Table S4.** CD4 and CD8 positive response rates to any antigen by polychromatic flow cytometry

|  | CD8+ T-cell response rates, number positive/total (% response rate) | | | | |
| --- | --- | --- | --- | --- | --- |
| Function | Baseline | Placebo | Group B | Group C | Group D |
|  | Pre-Vac.1 | 2 Wks Post 2^nd^ vaccination | | | |
| CD107a | 1/36 (2.8) | 0/11 | 4/8 (50) | 3/9 (33.3) | 6/7 (85.7) |
| IFNγ | 4/36 (11.1) | 0/11 | 4/8 (50) | 4/9 (44.4) | 6/7 (85.7) |
| IL-2 | 0/36 (0) | 0/11 | 2/8 (25) | 4/9 (44.4) | 3/7 (42.9) |
| TNFα | 2/36 (5.6) | 0/11 | 3/8 (37.5) | 3/9 (33.3) | 6/7 (85.7) |
|  | CD4+ T-cell response rates, number positive/total # (% response rate) | | | | |
| Function | Baseline | Placebo | Group B | Group C | Group D |
|  | Pre-Vaccination | 2 Wks Post 2^nd^ vaccination | | | |
| CD107a | ^a^2/36 (5.6) | 1/11 (9.1) | 0/8 | 1/9 (11.1) | 0/7 |
| IFNγ | 3/36 (8.3) | 1/11 (9.1) | 2/8 (25) | 2/9 (22.2) | 3/7 (42.9) |
| IL-2 | 1/36 (2.8) | 0/11 | 5/8 (62.5) | 1/9 (11.1) | 1/7 (14.3) |
| TNFα | 1/36 (2.8) | 0/11 | 4/8 (50) | 1/9 (11.1) | 5/7 (71.4) |

^a^Positive response defined as mock-subtracted response > 2 x MOCK and > 97.5th percentile over all baseline CD4 or CD8

Group B: Ad35-GRIN/ENV, 2x10^10^ vp

Group C: Ad35-GRIN/ENV, 2x10^11^ vp

Group D: Ad35-GRIN, 1x10^10^ vp
