# Supplementary material for: A Phase I Double Blind, Placebo-Controlled, Randomized Study of a Multigenic HIV-1 Adenovirus Subtype 35 Vector Vaccine in Healthy Uninfected Adults
Source: PLoS One. 2012 Aug 3;7(8):e41936. doi: 10.1371/journal.pone.0041936 (PMC3411704; doi:10.1371/journal.pone.0041936)
Supplement: Table S5 — Summary of antibody response frequencies. (DOCX) [file pone.0041936.s008.docx]

**Table S5. Summary of antibody response frequencies**

|  | Number positive/Number tested (% positive) | | | | | |  |
| --- | --- | --- | --- | --- | --- | --- | --- |
|  | Env ELISA | | Gag ELISA | | Ad35 Neutralization | |  |
|  | 4 w Post 1^st^ | 2 w Post 2^nd^ | 4 w Post 1^st^ | 2 w Post 2^nd^ | 4 w Post 1^st^ | 2 w Post 2^nd^ |  |
| Group A | 8/10 (80) | 9/9 (100) | 0/10 | 0/9 (0) | 2/10 (20) | 3/9 (33) |  |
| Group B | 9/10 (90) | 8/8 (100) | 0/10 | 3/8 (38) | 0/10 (0) | 3/8 (38) |  |
| Group C | 10/10 (100) | 9/9 (100) | 0/10 | 8/9 (89) | 3/10 (30) | 8/9 (89) |  |
| Group D |  |  | 0/10 | 5/7 (71) | 1/10 (10) | 4/7 (57) |  |

Group A: Ad35-GRIN/ENV, 2x10^9^ vp

Group B: Ad35-GRIN/ENV, 2x10^10^ vp

Group C: Ad35-GRIN/ENV, 2x10^11^ vp

Group D: Ad35-GRIN, 1x10^10^ vp
