# Supplementary material for: A Phase I Double Blind, Placebo-Controlled, Randomized Study of a Multigenic HIV-1 Adenovirus Subtype 35 Vector Vaccine in Healthy Uninfected Adults
Source: PLoS One. 2012 Aug 3;7(8):e41936. doi: 10.1371/journal.pone.0041936 (PMC3411704; doi:10.1371/journal.pone.0041936)
Supplement: Protocol S1 — Trial Protocol. (PDF) [file pone.0041936.s009.pdf]

Ad35-GRIN/ENV US Phase I Trial

**Protocol Title:**	A Phase I placebo-controlled, double-blinded (in terms of vaccine or placebo), randomized dose-escalation trial to evaluate the safety and immunogenicity of Ad35-GRIN/ENV and Ad35-GRIN HIV Vaccines in healthy adult volunteers.
**Protocol Number:**	IAVI B001
**Phase:**	Phase I
**Regulatory Investigational Product Number:**	**BB-IND# 13876**
**Sponsor:**	International AIDS Vaccine Initiative (IAVI)<br>110 William Street, 27th Floor<br>New York, New York 10038-3901<br>USA
**Sponsor Status:**	Non-Profit Organization
**Collaborating Sites:**	University of Rochester Medical Center<br>601 Elmwood Avenue<br>Rochester, NY 14642, USA
**Date of Protocol**	**16 October 2009**
**Version:**	**2.0**

The **CONFIDENTIAL INFORMATION** in this document is provided to you as an Investigator, potential Investigator, or Consultant, for review by you, your staff, and applicable institutional review boards (IRBs) and/or independent ethics committees (IECs). It is understood that the information will not be disclosed to others, except to the extent necessary to obtain ethical and regulatory approval from the respective committee's agencies and informed consent from those persons to whom the Investigational Product may be administered.

SYNOPSIS

**TITLE:**	A Phase I placebo-controlled, double-blinded (in terms of vaccine or placebo), randomized dose-escalation trial to evaluate the safety and immunogenicity of Ad35-GRIN/ENV and Ad35-GRIN HIV Vaccines in healthy adult volunteers.
**PROTOCOL NUMBER:**	IAVI B001
**Regulatory Investigational Product Number**:	**BB-IND# 13876**
**PHASE:**	Phase I
**SPONSOR:**	International AIDS Vaccine Initiative (IAVI)<br>110 William Street, 27th Floor<br>New York, New York 10038-3901,<br>USA
**Sponsor Status:**	**Non-Profit Organization**
**OBJECTIVES:**	***Primary:***<br>• To evaluate the safety of Ad35-GRIN/ENV administered intramuscularly at 0 and 6 months<br>• To evaluate the safety of Ad35-GRIN administered intramuscularly at 0 and 6 months<br><br>***Secondary:***<br>• To evaluate the immunogenicity of Ad35-GRIN/ENV administered intramuscularly at 0 and 6 months<br>• To evaluate the immunogenicity of Ad35-GRIN administered intramuscularly at 0 and 6 months<br><br>**Other**<br>• To study Ad35-GRIN/ENV shedding
**ENDPOINTS:**	***Primary***:<br><br>***Safety:***<br>• Proportion of volunteers with severe and very severe local reactogenicity events (pain, tenderness, erythema, skin discoloration, skin damage (vesiculation/ulceration), induration, formation of crust or scab)<br>• Proportion of volunteers with severe and very severe systemic reactogenicity events (fever, chills, headache, nausea, vomiting, malaise, myalgia, arthralgia)<br>• Proportion of volunteers with severe and very severe other adverse events (including laboratory abnormalities)<br>• Proportion of volunteers with vaccine-related Serious Adverse Events<br>• Proportion of volunteers with mild and moderate local and systemic reactogenicity events

	• Proportion of volunteers with mild and moderate other adverse events<br><br>***Secondary***:<br>***Immunogenicity:***<br>• Proportion of volunteers with HIV-1 specific T-cell responses by ELISPOT assay. If robust responses occur, they will be characterized by multiparameter flow cytometry for detection of intracellular cytokines, functional, surface and memory markers<br>• Proportion of volunteers showing *in vitro* inhibition of HIV replication<br>• Proportion of volunteers with antibodies to HIV antigens<br>• Proportion of volunteers with neutralizing antibodies to Ad35<br>• Proportion of volunteers with Ad35 vector-specific cell-mediated response assessed by ELISPOT assay
**STUDY DESIGN TABLE**	(see table below)
**METHODS:**	See Schedule of Procedures; Appendix A
**STUDY POPULATION:**	Healthy male or female adults 18-50 years of age, who are not infected with HIV-1 or HIV-2; who are available for the duration of the trial and willing to undergo HIV testing and use an effective method of contraception; who report low-risk behavior for HIV infection; and who, in the opinion of the principal investigator or designee, understand the study and can provide written informed consent.<br><br>Principal exclusion criteria include: HIV-1 or HIV-2 infection; pregnancy and lactation; a chronic disease which in the opinion of the investigator makes the volunteer unsuitable for the trial; recent

Study design table:

		Dose	N	Months	
				0	6
**A**	Ad35-GRIN/ENV	$2x10^9$ vp	10/4	X	X
**B**	Ad35-GRIN/ENV	$2x10^{10}$ vp	10/4	X	X
**C**	Ad35-GRIN/ENV	$2x10^{11}$ vp	10/4	X	X
**D**	Ad35-GRIN	$1x10^{10}$ vp	10/4	X	X

	vaccination or receipt of a blood product or experimental agent; and previous severe vaccine reaction or having previously received an HIV vaccine candidate.
**NUMBER OF VOLUNTEERS:**	Approximately 56 volunteers (four dose groups of 10 vaccine and 4 placebo recipients each).

**DESCRIPTION OF INVESTIGATIONAL PRODUCT (Vaccine and Placebo):**	Ad35-GRIN/ENV consists of two vectors Ad35-GRIN and Ad35-ENV formulated in a 1:1 ratio and filled into single use vials for intramuscular injection<br>• Ad35-GRIN is a recombinant replication-incompetent adenovirus serotype 35 that contains HIV-1 subtype A *gag*, *reverse transcriptase, integrase*, and *nef* genes.<br>• Ad35-ENV is a recombinant replication-incompetent adenovirus serotype 35 that contains HIV-1 subtype A gp140 *env* gene<br><br>Ad35 placebo is composed of 1 mM $MgCl_2$, Tween 80 - 54 mg/L, 1M Saccharose, 150mM NaCl, 10mm Tris/HCl, in Water For Injection (WFI), Final pH 8.5.

Vaccine/ Placebo	Dosage Level	Total Injected Volume	Route of Administration
Ad35-GRIN/ENV	$2x10^9$, $2x10^{10}$, and $2x10^{11}$ vp per dose	0.5 mL	IM
Ad35-GRIN	$1x10^{10}$ vp per dose	0.5 mL	IM
Ad35 Placebo	NA	0.5 mL	IM

IM= Intramuscular

Product appearance: Placebo is colorless. Vaccine is a whitish liquid and limpid or slightly turbid liquid depending on the virus concentration.

**RANDOMIZATION and DOSAGE ESCALATION:**	Participants will be randomized to receive either vaccine or placebo within a dose group (A, B, C, D). There is no randomization on the dose. The Safety Review Board will authorize the dose-escalation after review of safety data of the lower dose group.<br><br>Prior to enrolment into the mid-dose group, the SRB will review the safety of the low dose, after the first vaccination, based on a compilation of blinded data from the first 9 volunteers enrolled. Any severe and very severe events will be provided to the SRB as an

	update prior to proceeding with the next dosage level.<br><br>Enrolment into the high dose group will depend on the review of the safety data of the mid-dose group, following the procedure described above.
**BLINDING:**	Study site staff and volunteers will not be blinded with respect to the dose group but will be blinded to the allocation of placebo or vaccine.
**DURATION OF STUDY PARTICIPATION:**	Volunteers will be screened up to 42 days before vaccination and volunteers in all groups will be followed for 12 months after receiving their last vaccination (18 months total participation). It is anticipated that it will take approximately 5 months to enroll this study.

**EVALUATION FOR INTERCURRENT HIV INFECTION:**	Volunteers will be tested for HIV antibodies by ELISA according to the Schedule of Procedures. If the ELISA is positive, a pre-defined testing algorithm will be followed to determine whether antibodies have been induced by the vaccine or the volunteer has become infected with HIV through exposure in the community. HIV testing at additional time points may be performed at the discretion of the volunteer and principal investigator as medical or social circumstances arise.
**STATISTICAL CONSIDERATIONS:**	Data will be recorded on the Case Report Form (CRF). At the end of the study, a full analysis will be prepared according to a pre-specified data analysis plan.<br><br>Safety and tolerability will be addressed by examining overall rates of reactogenicity events and severe and very severe adverse events and SAEs that might be associated with vaccination and the number of volunteers who experience these events. All clinical and routine laboratory data will be included in the safety analysis. Volunteers will be classified as responders or non-responders based on the results of the immune assays.

TABLE OF CONTENTS

ABBREVIATIONS **9**

CONTACT INFORMATION **11**

1.1 SPONSOR CONTACT INFORMATION 11
1.2 INVESTIGATOR CONTACT INFORMATION 12

2.0 SIGNATURE PAGE **13**

3.0 INTRODUCTION AND BACKGROUND INFORMATION **14**

3.1 STUDY RATIONALE 14
3.1.1 Experience with Adenoviral Vector Serotype 5 HIV Vaccines 16
3.1.2 Adenoviral Vector Serotype 35 Selection 16

4.0 STUDY OBJECTIVES **18**

4.1 PRIMARY OBJECTIVES 18
4.2 SECONDARY OBJECTIVES 18
4.3 OTHER 18

5.0 STUDY ENDPOINTS AND STUDY DESIGN **18**

5.1 STUDY ENDPOINTS 18
5.1.1 Primary Endpoints 18
5.1.2 Secondary Endpoints 19
5.2 STUDY DESIGN 19
5.2.1 Duration of the Study 20
5.2.2 Study Population 20
5.2.3 Inclusion Criteria 20
5.2.4 Exclusion Criteria 22
5.2.5 Recruitment of Study Volunteers 23

6.0 STUDY VISITS **24**

6.1 SCREENING PERIOD 24
6.2 VACCINATION VISIT 25
6.3 POST-VACCINATION VISITS 26
6.4 ADDITIONAL FOLLOW-UP VISITS 26
6.5 UNSCHEDULED VISITS/CONTACT 26
6.6 FINAL VISIT/EARLY TERMINATION VISIT 27

7.0 STUDY PROCEDURES **27**

7.1 INFORMED CONSENT PROCESS 27
7.2 MEDICAL HISTORY AND PHYSICAL EXAMINATION 28
7.3 HIV RISK ASSESSMENT, HIV TESTING AND HIV TEST COUNSELLING 29
7.4 FAMILY PLANNING COUNSELLING 29
7.5 BLOOD COLLECTION 29
7.6 COMPENSATION FOR PARTICIPATION 29
7.7 RANDOMIZATION AND BLINDING 29
7.8 UNBLINDING PROCEDURE FOR INDIVIDUAL VOLUNTEERS 30

8.0 INVESTIGATIONAL PRODUCT **30**

8.1 DESCRIPTION 30
8.1.1 Active vaccine 31
8.1.2 Placebo 31
8.2 SHIPMENT AND STORAGE 32

8.3 DISPENSING AND HANDLING 32
8.4 ADMINISTRATION 32
8.5 ACCOUNTABILITY AND DISPOSAL 32

9.0 ASSESSMENTS 33

9.1 SAFETY ASSESSMENTS 33
9.1.1 Local reactogenicity 33
9.1.2 Systemic reactogenicity 33
9.1.3 Other adverse events 33
9.1.4 Concomitant Medications 34
9.1.5 Routine laboratory parameters 34
9.1.6 Specific screening tests: 35
9.2 IMMUNOGENICITY ASSESSMENTS 35
9.2.1 Antibody Responses 35
9.2.2 Cellular Responses 35
9.2.3 PBMC, Serum and Plasma Storage 36
9.3 OTHER ASSESSMENTS 36
9.3.1 HLA Typing 36
9.3.2 HIV test 36
9.3.3 Pregnancy Test 36
9.3.4 Antibody response to the Ad35 vector 36
9.3.5 Viral inhibition assay 37
9.3.6 Viral shedding 37

10.0 ADVERSE EVENTS 37

10.1 DEFINITION 37
10.2 ASSESSMENT OF SEVERITY OF ADVERSE EVENTS 37
10.3 RELATIONSHIP TO INVESTIGATIONAL PRODUCT 38
10.4 SERIOUS ADVERSE EVENTS 39
10.5 CLINICAL MANAGEMENT OF ADVERSE EVENTS 39
10.6 PREGNANCY 40
10.7 INTERCURRENT HIV INFECTION 40

11.0 MANAGEMENT OF HIV ISSUES DURING AND FOLLOWING THE STUDY 40

11.1 HIV TESTING 40
11.2 SOCIAL DISCRIMINATION AS A RESULT OF AN ANTIBODY RESPONSE TO VACCINE 41
11.3 HIV INFECTION 41
11.3.1 Counselling 41
11.3.2 Referral for Support and/or Care 42

12.0 DISCONTINUATION OF VACCINATIONS AND/OR WITHDRAWAL FROM STUDY 42

12.1 DISCONTINUATION OF VACCINATIONS 42
12.2 FOLLOW-UP AFTER DISCONTINUATION OF FURTHER VACCINATIONS 43
12.3 WITHDRAWAL FROM THE STUDY (EARLY TERMINATION) 43
12.4 FOLLOW-UP WITHDRAWAL FROM THE STUDY (EARLY TERMINATION) 43

13.0 DATA HANDLING 44

13.1 DATA COLLECTION AND RECORD KEEPING AT THE STUDY SITE 44
13.2 DATA COLLECTION AND TRANSFER AT THE IAVI CORE LABORATORY 44
13.3 DATA ENTRY AT THE STUDY SITE 44

14.0 STATISTICAL CONSIDERATIONS 45

14.1 SAMPLE SIZE 45
14.2 STATISTICAL POWER AND ANALYSIS 45

15.0 QUALITY CONTROL AND QUALITY ASSURANCE **47**
16.0 DATA AND BIOLOGICAL MATERIAL **47**
17.0 ADMINISTRATIVE STRUCTURE **47**
17.1 SAFETY REVIEW BOARD 47
17.1.1 Content of Interim Review 48
17.1.2 Indications for Discontinuation of Vaccinations in all Volunteers 48
17.2 STUDY SUPERVISION 48
17.3 STUDY MONITORING 49
17.4 INVESTIGATOR'S RECORDS 49
18.0 INDEMNITY **49**
19.0 PUBLICATION **49**
20.0 ETHICAL CONSIDERATIONS **50**
APPENDIX A: SCHEDULE OF PROCEDURES **51**
APPENDIX B: ADVERSE EVENT GRADING TOXICITY TABLE **53**
REFERENCES **66**

ABBREVIATIONS

Abbreviation	Term
Ad35	Adenovirus serotype 35
Ad35-GRIN/ENV HIV Vaccine	A mixture of replication-incompetent recombinant adenovirus serotype 35 vectors expressing HIV-1 subtype A *gag, RT, integrase*, *nef* (GRIN) and HIV-1 *envelope* (ENV)
Ad35-GRIN	Replication-incompetent recombinant adenovirus serotype 35 vectors expressing HIV-1 subtype A *gag, RT, integrase*, *nef* (GRIN)
AE	Adverse Event
AIDS	Acquired Immunodeficiency Syndrome
ALT	Alanine-Aminotransferase
AST	Aspartate-Aminotransferase
CFC	Cytokine Flow Cytometry
CMI	Cell Mediated Immunity
CMV	Cytomegalovirus
CRF	Case Report Form
CTL	Cytotoxic T Lymphocyte
DCC	Data Coordinating Center
DNA	Deoxyribonucleic Acid
ELISA	Enzyme Linked Immunosorbent Assay
ELISPOT	Enzyme Linked Immunospot
ENV	HIV-1 subtype A *envelope* gene
GCP	Good Clinical Practice
GRIN	Fused sequences of HIV-1 subtype A *gag, C-terminal two-thirds of the polymerase precursor comprised of reverse transcriptase, RNAse and integrase, and nef* genes
HIV	Human Immunodeficiency Virus
HLA	Human Leukocyte Antigen
HPLC	High Performance Liquid Chromatography
HSV	Herpes Simplex Virus

Abbreviation	Term
IAVI	International AIDS Vaccine Initiative
ICH	International Conference on Harmonisation
ICS	Intracellular Cytokine Staining
IFN-γ	Interferon-gamma
kg	Kilogram
mg	Milligram
PCR	Polymerase Chain Reaction
pfu	Plaque Forming Units
PBMC	Peripheral Blood Mononuclear Cells
PCP	*Pneumocystis carinii* pneumonia
rAd35	Replication-incompetent recombinant adenovirus serotype 35 vector
RPR	Rapid Plasma Reagin
SAE	Serious Adverse Event
SIV	Simian Immunodeficiency Virus
SOP	Standard Operating Procedure
SOM	Study Operations Manual
SRB	Safety Review Board
STD	Sexually Transmitted Disease
TPHA	*Treponema Pallidum* Hemagglutination Assay
VIA	Viral Inhibition Assay
vp	Virus particle

1.0. CONTACT INFORMATION

1.1 Sponsor Contact Information

Trial Sponsor

International AIDS Vaccine Initiative (IAVI)<br>110 William Street, 27th Floor<br>New York, NY 10038-3901<br>USA	Telephone:<br>Fax:<br>Mobile:	+ 1 212 847 1068<br>+ 1 212 847 1113<br>+ 1 646 206 6611

Chief Medical Officer

Patricia Fast MD, PhD<br>Executive Director, Medical Affairs<br>International AIDS Vaccine Initiative (IAVI)<br>110 William Street, 27th Floor<br>New York, NY 10038-3901, USA	Telephone:<br>Fax:<br>Mobile:<br>E-mail:	+1 212 847 1068<br>+ 1 212 847 1113<br>+ 1 646 206 6611<br>pfast@iavi.org

Medical Monitor

Jean-Louis Excler, MD<br>Senior Medical Director<br>International Aids Vaccine Initiative (IAVI)<br>Route de Duillier 11A<br>1270 Trelex<br>Switzerland	Telephone:<br>Mobile:<br>E-mail:	+ 41 22 369 1391<br>+ 41 76 418 4269<br>jlexcler@iavi.org

Data Coordinating Center

The EMMES Corporation<br>401 N. Washington Street<br>Suite 700<br>Rockville, Maryland 20850<br>USA	Telephone:<br>Fax:<br>E-mail:	+ 1 301 251 1161<br>+ 1 301 251 1355<br>iavitrials@emmes.com

Trial Monitoring

International AIDS Vaccine Initiative (IAVI)<br>110 William Street, 27th Floor<br>New York, NY 10038-3901, USA	Telephone:<br>Fax:	+ 1 212 847 1111<br>+ 1 212 847 1113

Sponsor's Clinical Program Manager

Wendy Komaroff<br>Senior Clinical Program Manager<br>International AIDS Vaccine Initiative (IAVI)<br>110 William Street, 27th Floor<br>New York, NY 10038-3901, USA	Telephone:<br>Fax:<br>Mobile:<br>E-mail:	+1 212 847 1070<br>+ 1 212 847 1113<br>+ 1 646 431 7697<br>wkomaroff@iavi.org

Core Laboratory

Jill Gilmour, Ph.D	Telephone:	+44 208 746 5098\
Senior Director, Clinical Research	Fax:	+44 208 746 5096
International AIDS Vaccine Initiative, Core Laboratory	Mobile:	+ 44 7810 202 699
5th Floor St Stephen's Centre	E-mail	jgilmour@iavi.org
Chelsea and Westminster Hospital		
369 Fulham Road		
London SW10 9NH		

Josephine H Cox, Ph.D	Telephone:	+1 301 251 8360
International AIDS Vaccine Initiative	Fax:	+1 301 424 3120
1600 Gude Drive, Room 114	Mobile:	+1 646 639 5904
Rockville, MD 20850, USA	E-mail	jcox@iavi.org

Gwynn Stevens, Ph.D	Telephone:	+27 11 325 4737
International Aids Vaccine Initiative	Fax:	+27 11 759 6210
Director Clinical Laboratory Program	Mobile:	+27 83 415 0818
6 Albury Park, Dunkeld West	E-mail:	gstevens@iavi.org
Johannesburg, South Africa		

Tony Tarragona, Ph.D.	Telephone:	+ 44 20 8846 6300
Laboratory Manager/Administrator	Fax:	+ 44 7940 510090
IAVI Core Lab	Mobile:	+ 44 20 8846 6301
Imperial College London, Faculty of Medicine	E-mail	t.tarragona@imperial.ac.uk
St. Stephen’s Centre, 5th Floor		
Chelsea Campus		
369 Fulham Road		
London SW10 9NH, UK		

1.2 Investigator Contact Information

Principal Investigator

Michael Keefer	Telephone:	+1 585 275 8058
Box # 689 (Room # 3-6206)	Fax:	+1 585 442 9328
University of Rochester Medical Center	Mobile:	+1 585 732 2434
601 Elmwood Ave.	Email:	Michael_Keefer@URMC.Rochester.edu
Rochester, NY 14642, USA		

2.0 SIGNATURE PAGE

The signatures below constitute the approval of this protocol and the appendices and provide the necessary assurances that this study will be conducted in compliance with the protocol, GCP and the applicable regulatory requirement(s).

Sponsor:

Signed: ____ Date: ________________

Dr. Patricia Fast
Chief Medical Officer, IAVI

Principal Investigator:

Signed: ____ Date: ________________

Michael Keefer, MD
University of Rochester Medical Center

Instructions: The Principal Investigator at the study site will sign and date two copies of the protocol signature page indicating that he/she agrees to conduct the study in accordance with the protocol.

One copy of the original, signed protocol signature page will be returned to IAVI where it will be archived. The other copy of the original signed and dated protocol signature page must be filed in the investigator's site file.

3.0 INTRODUCTION AND BACKGROUND INFORMATION

According to the Joint United Nations Program on HIV/AIDS and the World Health Organization, as of the end of 2007, 33 million people were estimated to be living with HIV/AIDS, with 96% residing in the developing world It is estimated that in 2007 alone, 2.7 million were newly infected with HIV and 2.1 million died of AIDS [1].

Sub-Saharan Africa has the largest burden of HIV/AIDS. In sub-Saharan Africa there is a disproportionate impact on females and young people of ages 15-24 years; the ratio of HIV-infected females to males, on average, is 3 to 2, but in the age 15-24 year group the ratio is 3 to 1. HIV prevalence on average is 1.7 times higher in urban areas than in rural areas. HIV prevalence is leveling off, but at an exceptionally high level and with the apparent stabilization in the prevalence attributed to the numbers of newly infected people being roughly the same as the number dying of AIDS-related causes. Worldwide, AIDS is the leading cause of premature death among both men and women aged 15-59 years. Average life expectancy has declined in 38 countries since 1999 primarily as a result of AIDS. In seven African countries where the prevalence exceeds 20%, the average life expectancy of a person born between 1995 and 2000 is 50 years, which is 12 years less than in the absence of AIDS [2].

Despite considerable progress in care and treatment and prevention efforts, the HIV/AIDS epidemic is still rampant. Beyond the human tragedy of HIV/AIDS, the costs of the epidemic pose a significant impediment to the economic growth and political stability of many countries. In developing countries and in segments of the U.S. population, anti-HIV therapies are frequently beyond financial reach. Accordingly, effective, low-cost tools for HIV prevention, such as a vaccine, are urgently needed to bring the HIV epidemic under control. For this reason, IAVI is committed to the development of safe, effective vaccines to prevent HIV infection and AIDS worldwide.

The effort to develop an effective preventive vaccine against HIV-1 infection is challenged by the wide genetic diversity of HIV-1 among different isolates. Analysis of genomic sequences from different regions in the world has identified at least 9 major subtypes (A, B, C, D, F, G, H, J and K) and dozens of recombinant forms [3], but together, the A, B and C subtypes represent the viral subtypes responsible for about 75%-85% of new HIV infections in the world [4,5]. Subtype D is present in parts of East Africa. Subtypes B and D are phylogenetically closer to each other than other HIV subtypes [6].

To be effective, an HIV vaccine will have to induce appropriate immune responses that are potent and long-lasting. Ideally a vaccine would be delivered prior to risk of exposure to HIV. The immune correlates for protection that may be required are not known, but experimental and epidemiological evidence suggests that both high levels of HIV-specific neutralizing antibody and long-lasting CD8+ and CD4+ T-cell responses are needed [7,8].

3.1 Study Rationale

This study is a Phase I dose-escalation clinical trial to evaluate the safety and immunogenicity of Ad35-GRIN/ENV or Ad35-GRIN HIV Vaccines (replication-incompetent recombinant adenovirus serotype 35 expressing HIV-1 subtype A *gag, RT, integrase*, *nef* (GRIN) and HIV-1 *envelope* (ENV)) and administered in a homologous prime-boost regimen by intramuscular route at Months 0 and 6.

The recombinant adenovirus vector (rAd) vaccine design is based on the concept of immunization by gene transfer. Recombinant adenovirus vector vaccines offer the positive attributes of immune stimulation by replication incompetent viral vaccines, without adjuvant. The hope is that by using a vaccine that can infect cells and present endogenously produced HIV proteins, such a vaccine could mimic the effectiveness of live attenuated simian AIDS vaccine in macaques. Preclinical studies [9 10 11 12 13 14] and clinical studies [15 16 17 18 19] show that immune responses against HIV can be elicited by direct gene transfer of immunogen-expressing HIV genes via rAd. The major advantage of rAd immunization appears to be its efficacy in transducing host cells and priming the induction of $CD8^+$ cytotoxic T lymphocytes (CTL) responses, which are considered an important element in controlling HIV-1 viral replication [9 20 21 22 23 24 25 26]. There is an additional safety feature in that following entry into the target cells, the HIV-1 gene products will be expressed without the production of infectious adenovirus (Ad) or integration into the host genome. These gene products can be produced in cells that are not actively dividing.

Immunization with more than one immunogen (co-immunization) is an efficient regimen to induce immunity to multiple antigens. HIV-1 envelope (Env) and Gag gene products are the predominant immunogens used in current AIDS vaccines. Few studies however have evaluated possible immune interference when these two antigens are co-administered. Some studies have shown that it is possible to induce immune responses to all proteins of a multi-component vaccine [27]. Other studies have shown loss of immunogenicity against one or more vaccine components[28][29].

Reduced levels of specific antibodies as well as decreased cellular responses have been observed with combinations of genetic immunogens[30 31]. More specifically, immune interference during co-inoculation was studied in mice using *env* gp160 HIV-1 subtypes A, B and C, DNA *gag* p37 subtypes A and B, DNA *rev* subtype B and DNA *RT* subtype B genes all carried by separate DNA plasmids. The *env* genes alone induced significantly stronger cellular responses than when *env* genes were injected together with *gag* and *RT* genes [32].

Mice vaccinated with HIV-1 Env gp120 and Gag p55 plasmids in separate hind legs with each plasmid individually elicited high titer immune responses; however, when plasmids were co-inoculated, there was a reduction in the immune responses elicited to HIV-1 Gag p55 suggesting a possible Env interference [33]. A similar antigen competition, manifested by a relative reduction of CD8+ T-cell responses to Gag and Tat and lymphoproliferation responses to Gag, Env, Tat, and Nef was observed in macaques immunized with combined vaccines [34].

In humans, DNA plus NYVAC HIV-1 subtype C vaccine regimen induced T cell responses in 90% of vaccinees compared to 33% with NYVAC-HIV-1 vaccine alone. The vaccine-induced T cell responses were however strongest and most frequently directed against Env (91% of vaccines), but lower responses against Gag-Pol-Nef were also observed in 48% of vaccinees [28]. These findings may suggest Env interference. A similar pattern was observed following vaccination with MVA expressing HIV-1 Clade B'/C *env, gag, pol, nef*, and *tat* genes. T cell responses were mostly directed to Env, almost none against Gag and a few against other HIV proteins [29].

3.1.1 Experience with Adenoviral Vector Serotype 5 HIV Vaccines

The MRK adenovirus type 5 human immunodeficiency virus type 1 clade B *gag/pol/nef* vaccine was tested in phase I trial followed by a Phase IIB Test-of-Concept trial jointly with the HIV Vaccine Trial Network. The Vaccine Research Center (VRC) has developed a recombinant serotype 5 adenovector (rAd5) product composed of four rAd5 vectors that encode HIV-1 Env glycoproteins from subtypes A, B, and C, and Gag/Pol polyproteins from subtype B respectively. This vaccine has been evaluated as a single agent in Phase I studies and in Phase I and II studies in healthy human subjects as a boost following vaccination with a plasmid DNA prime vaccine. The study data obtained thus far from the clinical trials with the VRC rAd5 vaccine, as well as from the human clinical trials of rAd5-based vaccines developed by Merck [1335] suggest that rAd5 vaccines are well-tolerated and immunogenic at dosages from 10^9 through 10^{11} particle units.

A major potential limitation of rAd5 vector vaccines however, is the high prevalence of pre-existing immunity to adenovirus serotype 5 (Ad5) in human populations, which may diminish vaccine-induced immune responses [9] In addition, interim results of the Phase IIB efficacy study of the Merck rAd5 vaccine (HVTN 502, also known as the STEP Trial) suggest caution in administering rAd5 vector vaccines to subjects with pre-existing Ad5 antibody (Ab) at enrollment [36]. The STEP Trial enrolled 3000 men and women with an increased risk of exposure to HIV infection. The STEP Trial was halted for futility when the interim data reviewed by the Data and Safety Monitoring Board indicated that the MRK-rAd5 HIV vaccine was not preventing HIV infections and was not reducing the HIV viral load in participants who became HIV-infected. An unexpected safety concern was that there were more HIV infections in male vaccinated participants who already had neutralizing antibody to Ad5 at the time of enrollment (from a prior Ad5 naturally occurring infection) than the male placebo recipients from the same group, particularly among those who were not circumcised. There was only one HIV infection among the women in the STEP Trial, but preliminary data from the halted Phambili trial of the same regimen in South Africa suggest that the pattern in women is similar. Further evaluations of STEP Trial results are ongoing to try to identify the basis for this unexpected observation.

One strategy to overcome the hurdle of pre-existing humoral immunity is to select a human adenovirus serotype with low sero-prevalence, such as Ad35.

3.1.2 Adenoviral Vector Serotype 35 Selection

Many reports have confirmed high rates of Ad5 seroprevalence (with high levels of Ad5 neutralizing antibody), particularly in regions, such as sub-Saharan Africa, which are most at risk for HIV infection and where the need for safe and effective vaccine is most critical [37 38]. Therefore, an active area of research is the development of adenoviral vectors designed to evade dominant Ad5-specific immunity because they are based upon alternative adenovirus serotypes, which are less common.

In adult Africans representative of those who might be enrolled in a Phase I trial, the prevalence of Ad35 neutralizing antibodies was 19% with geometric mean titer (GMT) of 97 (N=360) compared to 89% and GMT at 846 (N=388) for Ad5 (IAVI, unpublished data). In another study, the percentage of Ad35 positive Sub-

Sahara African subjects (N=200, 18-65 years) was 17% and GMT 10-fold lower compared to Ad5 (GMT: 60 vs. 600) [29]. In the United States of America, the Ad35 seroprevalence is <7% in adults aged 20-70 years. Serum collected in Europe (Belgium, United Kingdom, and The Netherlands) demonstrated an even lower seroprevalence of 2-7% in healthy volunteers [29 38].

Thus, serotype Ad35 was selected for development as vaccine vector for the following reasons: (1) In Africa, there is a relatively low prevalence of antibodies to Ad35 (as described above); (2) Pre-existing immunity to Ad5 does not interfere with Ad35 immunogenicity in animals [39]; (3) Technology is available to IAVI with an improved vector construct back-bone able to generate product at a low virus particle to infectivity (VP:IU) ratio [30]; (4) Ad35 belongs to adenovirus subtype B that uses highly-expressed CD46 as receptor in contrast to Ad5 belonging to subtype C using the coxsackie-adenovirus receptor (CAR). Ad35 efficiently infects human monocyte-derived immature dendritic cells, which are important targets for eliciting potent and persistent immune responses [30]; (5) Since Ad35 only weakly interacts with coagulation factor X (FX), it may be preferable to Ad5 whose hexon binds to FX, leading to efficient liver targeting [40]; (6) Ad35 grows efficiently in HER96 cells; (7) the Ad35 used as vector in this protocol is replication-incompetent.

Outbreaks of acute respiratory disease caused by Ad35 wild type virus are rarely documented in civilian populations. Ad35 wild type is an uncommon serotype associated with very rare cases of serious pulmonary disease, hemorrhagic cystitis, and conjunctivitis, mostly in immuno-compromised patients [41 42 43 44 45] and very rarely in healthy individuals [46 47 48]. More recently, in large cohorts of military recruits and reviews of adenovirus-associated acute respiratory disease in healthy adolescents and adults, Ad35 wild type virus had not been incriminated as a cause of adenovirus illness [49 50 51].

The recombinant adenoviral serotype 35 vector (rAd35) HIV vaccine in this study, designated Ad35-GRIN/ENV, is designed to test the concept that rAd35 can deliver multiple HIV genes and produce beneficial immune responses with an acceptable safety profile. The *gag, RT, IN, nef* genes (abbreviated as GRIN) were identified within the HIV-1 sequence, designed as a fusion product, and codon optimized for human cell expression and translation. GRIN was selected based on the evidence that in a worldwide study assessing HIV-1-infected humans, the highest levels of T-cell responses (75–100%) were observed against gag, pol, and nef, regardless of either the donor's origin or the subtype of the infecting virus [52]. ENV gp140 was selected based on data showing that it appears immunogenic as a T-cell based vaccine antigen in humans and is capable of generating unexpectedly significant CTL cross-reactivity to ENV among the different HIV-1 subtypes [53 54 55 56]. Where appropriate, mutations have been introduced to abrogate the normal functions of the HIV antigens.

4.0 STUDY OBJECTIVES

4.1 Primary Objectives

To evaluate the safety of Ad35-GRIN/ENV administered intramuscularly at 0 and 6 months.

To evaluate the safety of Ad35-GRIN administered intramuscularly at 0 and 6 months

4.2 Secondary Objectives

To assess the immunogenicity Ad35-GRIN/ENV administered intramuscularly at 0 and 6 months.

To evaluate the immunogenicity of Ad35-GRIN administered intramuscularly at 0 and 6 Months

To compare the immunogenicity of Ad35-GRIN administered with and without Ad35-Env.

4.3 Other

To study Ad35-GRIN/ENV shedding

5.0 STUDY ENDPOINTS AND STUDY DESIGN

5.1 Study Endpoints

5.1.1 Primary Endpoints

Safety:

- Proportion of volunteers with severe and very severe local reactogenicity events (pain, tenderness, erythema, skin discoloration, skin damage (vesiculation, ulceration), induration, formation of crust or scab)
- Proportion of volunteers with severe and very severe systemic reactogenicity events (fever, chills, headache, nausea, vomiting, malaise, myalgia, arthralgia)
- Proportion of volunteers with severe and very severe other adverse events (including laboratory abnormalities)
- Proportion of volunteers with Serious Adverse Events
- Proportion of volunteers with mild and moderate local and systemic reactogenicity events
- Proportion of volunteers with mild and moderate other adverse events

5.1.2 Secondary Endpoints

Immunogenicity:

- Proportion of volunteers with HIV-1 specific T-cell responses by ELISPOT assay If robust responses occur, they will be characterized by multiparameter flow cytometry for detection of intracellular cytokines, functional, surface and memory markers
- Proportion of volunteers showing *in vitro* inhibition of HIV replication
- Proportion of volunteers with antibodies to HIV antigens
- Proportion of volunteers with neutralizing antibodies to Ad35
- Proportion of volunteers with Ad35 vector-specific cell-mediated response assessed by ELISPOT assay

5.2 Study Design

This study is a phase I dose-escalation randomized, placebo-controlled study designed to evaluate the safety and immunogenicity of Ad35-GRIN and Ad35-ENV filled in the same vial and administered as a single, combined vaccine. This is the first administration of this vaccine in humans. The study will be double blind with respect to vaccine or placebo. The vaccine will be administered intramuscularly at months 0 and 6 at three dose levels: 2×10^9, 2×10^{10}, and 2×10^{11} vp per dose. Volunteers in Group D will receive Ad35-GRIN at 1×10^{10} vp administered intramuscularly at months 0 and 6. Volunteers will be randomized to vaccine: placebo in a 10:4 ratio in each group.

The Safety Review Board will authorize the advance to the next dosage level after review of safety data from the first vaccination in the lower dosage group. Prior to enrolment into the mid-dosage group, the SRB will review the clinical and laboratory safety data of the low dosage, based on a compilation of blinded data from the first 9 volunteers enrolled (at least 50% vaccine recipients for a given dosage group). Any additional or subsequent severe or very severe events will be provided to the SRB as an update prior to proceeding with the next dosage level. SRB members will recommend dose-escalation on their medical judgment.

Enrolment into the high dosage group (Group C) will depend on the review of the safety data of the mid-dosage group, following the procedure described above.

Table 1
Study Design

		Dose	N	Months	
				0	6
**A**	Ad35-GRIN/ENV	$2x10^{9}$ vp	10/4	X	X
**B**	Ad35-GRIN/ENV	$2x10^{10}$ vp	10/4	X	X
**C**	Ad35-GRIN/ENV	$2x10^{11}$ vp	10/4	X	X
**D**	Ad35-GRIN	$1x10^{10}$ vp	10/4	X	X

5.2.1 Duration of the Study

Volunteers will be screened up to 42 days before vaccination (90 days for Ad35 neutralizing antibody screening) and will be followed for 12 months after the last vaccination (18 months total study participation). It will take approximately 5 months to enroll 56 volunteers. Thus, the total duration of the study would be approximately 23 months.

5.2.2 Study Population

The study population consists of healthy male or female adults aged 18-50 years who are not infected with HIV, do not report risk for HIV infection, available for the duration of the trial, willing to undergo HIV testing, use an effective method of contraception, and who, in the opinion of the investigator or designee, understand the study and provide written informed consent.

Approximately 56 volunteers (40 Vaccine recipients, 16 placebo recipients) who meet all eligibility criteria will be included in the study.

5.2.3 Inclusion Criteria

The Investigator will use his/her best clinical judgment in considering a volunteer's overall fitness for trial participation even if all inclusion/exclusion criteria are met.

1. Healthy males and females, as assessed by a medical history, physical exam, and laboratory tests;

2. At least 18 years of age on the day of screening and no greater than 50 years (not yet reached their 51st birthday) on the day of first vaccination;

3. Willing to comply with the requirements of the protocol and available for follow-up for the planned duration of the study (screening plus 18 months, see schedule of procedures);

4. In the opinion of the Principal Investigator or designee, has understood the information provided. Written informed consent needs to be given before any study-related procedures are performed;

5. Amenable to HIV risk reduction counseling, committed to maintaining behavior consistent with low risk of HIV exposure through the last required protocol clinic visit, and willing to continue 5 years of annual follow-up contact.

6. Demonstrates understanding (assessment of understanding will be performed) of the risk for harm observed in the STEP Study results.

7. Assessed by the clinic staff as being at “low risk” for HIV infection on the basis of sexual behaviors within the 12 months prior to enrolment defined as follows:

   - Sexually abstinent OR
   - Had two or fewer mutually monogamous relationships with partners believed to be HIV-uninfected and who did not use illicit drugs (methamphetamines (crystal meth), heroin, cocaine, including crack cocaine or chronic marijuana abuse)OR
   - Had two or fewer partners believed to be HIV-uninfected and who did not use illicit drugs (methamphetamines (crystal meth), heroin, cocaine, including crack cocaine or chronic marijuana abuse), and with whom he/she regularly used condoms for vaginal and anal intercourse

8. Willing to undergo HIV Testing, HIV counseling and receive HIV Test results;

9. If sexually active female, using an effective method of contraception (hormonal contraceptive; diaphragm; Intra Uterine Device (IUD); condoms; anatomical sterility in self or partner) from screening until at least 4 months after last vaccination. All female volunteers must be willing to undergo urine pregnancy tests at time points as indicated in the Schedule of Procedures (Appendix A)

10. If sexually active male, willing to use an effective method of contraception (such as condoms, anatomical sterility) from screening until 4 months after the last vaccination.

5.2.4 Exclusion Criteria

1. Confirmed HIV-1 or HIV-2 infection

2. Detection of Ad35-specific serum neutralizing antibody

3. Reported high-risk behavior for HIV infection defined as:
   Within 12 months before vaccination, the volunteer has:
   - Had unprotected vaginal or anal sex with a known HIV infected person or a casual partner (i.e., no continuing established relationship)
   - Engaged in sex work for money or drugs.
   - Excessive daily alcohol use or frequent binge drinking or chronic marijuana use or use of other illicit drugs.
   - Recently acquired a sexually transmitted disease (STD) including syphilis, gonorrhoea, non-gonococcal urethritis, *Trichomonas vaginalis*, symptomatic *Herpes genitalis* (HSV-2), chlamydia, pelvic inflammatory disease (PID), mucopurulent cervicitis, epididymitis, proctitis, lymphogranuloma venereum, chancroid, or hepatitis B).
   - Has a high-risk partner either currently or had such a partner within the previous 12 months.

4. Any clinically significant abnormality on history or examination, including history of immunodeficiency or autoimmune disease; use of systemic corticosteroids (the use of topical steroids and inhaled steroids for sinus decongestion are permitted), immunosuppressive, antiviral, anticancer, anti-tuberculosis, or other medications considered significant by the investigator within the previous 6 months;

5. Any clinically significant acute or chronic medical condition that is considered progressive or, in the opinion of the investigator, would make the volunteer unsuitable for the study.

6. Any of the following abnormal laboratory parameters listed below:
   - Hemoglobin <11.0 g/dL for women and <12.5 g/dL for men
   - Absolute Neutrophil Count (ANL): ≤ 999/mm^3
   - Absolute Lymphocyte Count (ALC): ≤ 500/mm^3
   - Platelets: ≤ 90,000 ≥ 550,000/mm^3
   - Creatinine: >1.1 ULN
   - AST: >1.25 x ULN
   - ALT: >1.25 x ULN
   - Urinalysis 2+ by urine dipstick
     - Blood (not due to menses);

- o Protein
- o Leucocytes

7. Confirmed diagnosis of hepatitis B (surface antigen HbsAg), hepatitis C (HCV antibodies), or active syphilis;

8. If female, pregnant or planning a pregnancy within 4 months after last vaccination; or lactating;

9. Receipt of live attenuated vaccine within the previous 60 days (live attenuated flu vaccine within 14 days) or planned receipt within 60 days after vaccination with Investigational Product or receipt of other vaccine within the previous 14 days or planned receipt within 14 days after vaccination with Investigational Product;

10. Receipt of blood transfusion or blood products within the previous 6 months;

11. Participation in another clinical study of an investigational product currently, within the previous 3 months or expected participation during this study;

12. Receipt of another investigational HIV vaccine candidate at any time;

13. History of severe or very severe local or systemic reactogenicity to vaccines or history of severe allergic reactions;

14. Major psychiatric illness, including any history of schizophrenia or severe psychosis, bipolar disorder requiring therapy, suicidal attempt or ideation in the previous 3 years.

15. Unwilling to forgo donations of blood, sperm, eggs, bone marrow or organs during the study.

16. Asplenia: any condition resulting in the absence of a spleen

5.2.5 Recruitment of Study Volunteers

Healthy adult male and female volunteers may be recruited through information presented via Internet, in community organizations, hospitals, colleges, other institutions and/or advertisements to the general public. This information will contain contact details and the basic criteria for enrolment in the study.

All methods used for recruitment must be approved by the Ethics Committee prior to implementation.

If other recruitment strategies are used, the sponsor needs to be informed. During the recruitment process it is important to ensure full counseling and full informed consent.

Study volunteers satisfying all criteria for enrolment after the screening visit will have to pass an Assessment of Understanding and verbalize understanding of any questions answered incorrectly.

6.0 STUDY VISITS

6.1 Screening Period

During screening, site personnel will perform the following procedures:

- Provide and/or review the Informed Consent Document and answer any questions about the study prior to obtaining written informed consent.
- Perform Assessment of Understanding with the volunteer.
- Obtain written informed consent prior to conducting any study procedures.
- Initially an IRB approved screening consent form may be used to allow for early screening of subjects.
- The results from this screening may be used to determine eligibility for this protocol as long as the tests and information are within the time period specified in the eligibility section.

If the volunteer agrees to participate, site personnel will:

- Provide a screening questionnaire to the volunteer for completion
- Perform an HIV risk assessment
- Perform a complete medical history (including concomitant medication)
- Perform a general physical examination, including height, weight, vital signs (pulse, respiratory rate, blood pressure and temperature), examination of skin, respiratory, cardiovascular and abdominal systems, and an assessment of cervical and axillary lymph nodes.
- Conduct pre-HIV test counseling
- Collect blood and urine specimens for all tests, as indicated in the Schedule of Procedures (Appendix A). Perform a pregnancy test for all female volunteers.

Screening laboratory test(s) may be repeated once at the discretion of the principal investigator or designee to investigate any isolated abnormalities.

Volunteers will be screened first for Ad35-specific serum neutralizing antibodies, within 90 days prior to the date of first vaccination. All other screening procedures must occur within 42 days prior to the date of first vaccination. If screening procedures are not performed within these time periods, they must be repeated. The complete medical history may be replaced by an interim medical

history and the Volunteer Information Sheet should be reviewed with the volunteer.

If a volunteer has signed the informed consent form, but does not meet the eligibility criteria, the records must be kept at the site.

6.2 VACCINATION VISIT

Prior to vaccination (Months 0 and 6), site personnel will:

- Review the Informed Consent Document with volunteers.
- Fill in eligibility checklist and decide the eligibility of the volunteer to participate in the vaccine trial.
- Perform an HIV risk assessment
- Answer any questions about the study
- Review interim medical history (including concomitant medications)
- Review (screening) safety laboratory data of previous visit.
- Perform a directed physical examination, including vital signs (pulse, respiratory rate, blood pressure and temperature), examination of vaccination site as well as an assessment of axillary lymph nodes and any further examination indicated by history or observation).
- Conduct pre HIV-test counseling.
- Collect blood, urine specimens as well as oropharyngeal swabs for all tests including viral shedding study, as indicated in the Schedule of Procedures (Appendix A).
- Perform a pregnancy test for all female volunteers and obtain results prior to vaccination.
- Baseline assessment of the site of vaccination and any systemic symptoms

The volunteer will be assigned a unique allocation number according to the instructions specified in the Study Operations Manual.

The Investigational Product will be administered as specified in Section 8.4, Administration.

Site personnel will closely observe volunteers for at least 30 minutes after vaccination for any acute reactogenicity events. At the end of the observation period site personnel will:

- Record vital signs (pulse, respiratory rate, blood pressure and temperature)
- Assess any local and systemic reactogenicity
- Assess any other adverse events.
- Provide the volunteer with a Memory Card and instructions to assist in the collection of reactogenicity events and AEs following vaccination

For subsequent vaccination visits (the 'preferred' window for the 2nd vaccination is +/- 7 days and the 'allowable' window is +/- 14 days) site personnel will perform the same procedures as above with the following exceptions:

- Review the routine safety laboratory parameters (Section 9.1.5) from the previous visit prior to each vaccination. If a volunteer has an abnormal laboratory value that is known at the time of vaccination, follow the specified guidelines (Section 12.1)
- Conduct pre HIV-test counseling if an HIV Test is required (Appendix A) or provide post-test counseling if the results of a prior HIV test are being provided to the volunteer.

A volunteer will be considered as ENROLLED once she/he has been randomly allocated to a specific vaccination regimen.

6.3 Post-Vaccination Visits

The volunteer will be asked to return to the clinic on Day 3 (+/- 1 day) and on Day 7 (+/- 2 days) and Day 14 (+/- 2 days) after each vaccination for an assessment. The study personnel will review the Memory card with the volunteer and record the information in the clinic chart.

The following procedures will be conducted at this visit:

- Review of interim medical history and use of concomitant medications.
- If symptoms are present, perform a symptom-directed physical examination.
- Assess local and systemic reactogenicity, as well as any other adverse events.
- Collection of blood and urine specimens, as well as oropharyngeal swabs for all tests including viral shedding study, as indicated in the Schedule of Procedures (Appendix A).

In case of adverse event(s), the volunteer will be assessed and followed up by the clinical team. Supplemental visit(s) for further investigation can be planned at the discretion of the clinical and Principal Investigators. Supplemental visit(s) may be recommended if clinically indicated or to clarify observations. All the required supportive care will be provided and referral services will be facilitated.

6.4 Additional Follow-up Visits

Assessments and procedures will be performed according to the Schedule of Procedures (Appendix A).

6.5 Unscheduled Visits/Contact

Unscheduled Visits/contacts are visits/contacts that are not described in the Schedule of Procedures (Appendix A). They may be performed at any time during the study. Unscheduled visits may occur:

- For administrative reasons, e.g., the volunteer may have questions for study staff or may need to re-schedule a follow-up visit.
- To obtain laboratory test results from a previous visit.
- In the event that a volunteer presents to the study site after having missed a scheduled study visit outside a scheduled visit window.
- For other reasons, as requested by the volunteer or site investigator.

All unscheduled visits will be documented in the volunteers' study records and on applicable source documents.

6.6 Final Visit/Early Termination Visit

Assessments and procedures will be performed according to the Schedule of Procedures (Appendix A).

Site personnel will:

- Review any adverse events and concomitant medications
- Perform a general physical examination, including height, weight, vital signs (pulse, respiratory rate, blood pressure and temperature), examination of skin, respiratory, cardiovascular and abdominal systems, and an assessment of cervical and axillary lymph nodes.
- Assess any local and systemic reactogenicity
- Collect blood and urine specimens for tests, as specified in the Schedule of Procedures (Appendix A).
- Perform a urine pregnancy test for all female volunteers

7.0 STUDY PROCEDURES

7.1 Informed Consent Process

A sample informed consent document is provided by the sponsor to the site. The site specific Informed Consent Document will be submitted and approved by IAVI and then the Institutional Ethics Committees of the site before use.

<u>Volunteer Information Sheet</u>
A qualified authorized member of the study staff will obtain informed consent by reviewing the Volunteer Information Sheet with the volunteer.

The following study specific elements are included:

1. It is unknown whether or not the Investigational Product will protect against HIV infection or disease or might enhance susceptibility to infection or disease

2. It may be possible that the vaccinated volunteer will develop antibodies against HIV following vaccination, which may produce a positive result in a routine HIV Antibody Test. Provisions have been made to distinguish between response to vaccine and HIV infection during and after the study. In case the volunteer has a positive result in a routine HIV Antibody Test, he/she will be followed until the result is no longer positive.

3. It is imperative that each volunteer should avoid any risky behavior for HIV infection during the entire period of the trial.

4. A sexually active volunteer should use a reliable form of contraception from screening, during the vaccination period until 4 months after the last injection.

5. A placebo will be administered to some volunteers in this study and these volunteers will receive placebo throughout the study.

Informed Consent Form

All volunteers will give their written informed consent to participate in the study on the basis of appropriate information and with adequate time to consider this information and ask questions.

The volunteer's consent to participate must be obtained by him/her signing and dating the informed consent form witnessed by a member of the study team. The members of the site personnel who are involved in conducting the informed consent discussions must also sign and date the Informed Consent Form.

The signed/marked and dated informed consent document must remain at the study site. A copy of the signed and dated informed consent form will be offered to the volunteer to take home if the volunteer is willing to receive the consent form. Those volunteers who do not wish to take a copy will be required to document that they declined to do so.

Family members, sexual partner(s) or spouse(s) will be offered education and counseling regarding a volunteer's participation in the study ONLY with the written consent of the participating volunteer.

7.2 Medical History and Physical Examination

At screening, a comprehensive medical history will be collected, including details of any known previous reaction to vaccinations, history of sexually transmitted diseases, contraceptive practices, and history of epilepsy.

A general physical examination includes the following: height, weight, vital signs (pulse, respiratory rate, blood pressure and temperature), examination of skin, respiratory, cardiovascular and abdominal systems, and an assessment of cervical and axillary lymph nodes. This examination will be conducted at screening and termination visits.

At each other study visit, an interim medical history and symptom directed physical examination will be performed. A directed physical examination will include vital signs, examination of vaccination site and any further examination indicated by history or observation.

7.3 HIV Risk Assessment, HIV Testing and HIV Test Counseling

Study staff will assess volunteers for past and current risk of HIV infection. A screening questionnaire and other tools may be used, according to site-specific procedures.

Additionally, study staff will perform pre-HIV test counseling (prior to collecting blood for an HIV test) and post-HIV test counseling (when HIV test results are available) according to the Schedule of Procedures (Appendix A). For more information on HIV testing and HIV-test counseling, see Section 10.

7.4 Family Planning Counseling

Study staff will counsel volunteers about the importance of preventing pregnancies and use of condoms, as well as other effective family planning methods. Condoms may be provided and volunteers may be referred to a family planning clinic if a contraceptive prescription is required, according to standard practice of the study site.

The family planning counseling will be performed at time points according to the Schedule of Procedures (Appendix A).

7.5 Blood Collection

Up to 20 mL of blood will be collected at the Screening Visit and up to 85 mL of blood will be collected at later visits, usually from the antecubital fossa, according to the Schedule of Procedures (Appendix A).

All specimens will be handled according to the procedures specified in the Study Operations Manual, as specified by the Core Lab SOPs and Study Analytical Plan.

In the event of an abnormal laboratory value, volunteers may be asked to have an additional sample collected at the discretion of the principal investigator or designee.

7.6 Compensation for Participation

Volunteers will be compensated for their time, effort and for costs to cover their travel expenses to the study site and any inconvenience caused due to study participation. Reimbursement will be made after the completion of each study visit. Site-specific reimbursement amounts will be documented in the consent form or the site-specific Volunteer Information Sheet approved by the site Ethics Committee.

7.7 Randomization and Blinding

Volunteers will be identified by a unique volunteer identification number.

The randomization schedule will be prepared by the statisticians at the Data Coordinating Center (DCC) prior to the start of the study. Volunteers will be assigned a specific allocation number. An unblinding list will be provided to the Pharmacist by the DCC for emergency use only.

This study is double-blinded. Study staff (investigator and clinical personnel monitoring the safety and laboratory assay results) and volunteers will be blinded with respect to the allocation of Investigational Product or Placebo.

Blinding will not apply to the dosage group assignment (A, B, C or D).

An unblinded pharmacist will prepare the syringe and deliver it to the person who injects the investigational product. Since vaccine and placebo may differ in their physical appearance, the person administering the dose should not be the same person who performs the assessment of safety and reactogenicity.

Volunteers will be informed about their assignment (vaccine or placebo) at the end of the study when all data are collected and all queries are resolved. If the study volunteer is unblinded during the course of the study and becomes aware of treatment assignment, further administration of the investigational product (vaccine or placebo) will be discontinued. The study volunteer will be followed up until the end of the study.

7.8 Unblinding Procedure for Individual Volunteers

Unblinding of an individual volunteer may be indicated in the event of a medical emergency for which the clinical management/medical treatment of the volunteer would be altered by knowledge of the group assignment. Whenever feasible, the IAVI Medical Monitor should be contacted prior to unblinding.

The unblinded information should be restricted only to a small group of individuals involved in clinical management/medical treatment of the volunteer (e.g., treating physician) and the blind should be maintained for those responsible for the study assessments.

The reasons for unblinding should be documented and the Data Coordinating Centre should be notified. The procedures and contact numbers for unblinding are outlined in the Study Operations Manual.

8.0 INVESTIGATIONAL PRODUCT

8.1 Description

The Ad35-GRIN/ENV and Ad35-GRIN HIV Vaccines and Placebo are manufactured by Transgene (France).

8.1.1 Active vaccine

Ad35-GRIN/ENV consists of two vectors Ad35-GRIN and Ad35-ENV formulated in a 1:1 ratio and filled into single use vials for intramuscular injection

- Ad35-GRIN is a recombinant replication-incompetent adenovirus serotype 35 expressing HIV-1 subtype A *gag*, *reverse transcriptase, integrase*, and *nef* genes.
- Ad35 ENV is a recombinant replication-incompetent adenovirus serotype 35 expressing HIV-1 subtype A gp140 *env* gene
- Where appropriate, mutations have been introduced to abrogate the normal functions of the HIV antigens.

Ad35-GRIN/ENV is supplied as a frozen sterile formulation in a 2-mL vial with a butyl stopper and aluminum seal. Each vial contains 0.725 mL of vaccine. The volume of administration is 0.5 mL, which will deliver a final dosage of $2x10^9$ vp or $2x10^{10}$ vp or $2x10^{11}$ vp per dose. Ad35-GRIN vials contain 0.725 mL of vaccine. The volume of administration, 0.5 mL, will deliver a final dosage of $1x10^{10}$ vp. The dose of the vaccine is provided as a total virus particle count measured by HPLC and expressed as viral particle (vp). The vaccine is formulated in buffer composed of Tris 10 mM pH 8.5, Sucrose 342,3 g/L, 1mM $MgCl_2$, Tween80 54 mg/L and 150mm NaCl in water for injection (used for diluting the purified bulk). Vaccine is a whitish liquid and limpid or slightly turbid liquid depending on the virus concentration.

8.1.2 Placebo

Placebo is provided as a frozen sterile suspension in a 2 mL vial with a butyl rubber stopper and aluminum seal. Each vial contains 0.725 mL of placebo. The volume of administration is 0.5 mL. The final formulation buffer (see description above) will be used as a placebo. The placebo will be manufactured, filter sterilized and filled under GMP in the same fill-finish container as will be used for the vaccine. Placebo is colorless.

The summary of the Investigational Product is shown in Table 2.

Table 2
Formulation of Investigational Product

Vaccine/Placebo	Dosage Level	Total Volume in Vial (mL)	Total Volume to be injected (mL)	Route of Administration
Ad35-GRIN/ENV	$2x10^9$ vp	0.725	0.50	IM
Ad35-GRIN/ENV	$2x10^{10}$ vp	0.725	0.50	IM
Ad35-GRIN/ENV	$2x10^{11}$ vp	0.725	0.50	IM
Ad35-GRIN	$1x10^{10}$ vp	0.725	0.50	IM
Ad35 Placebo	NA	0.725	0.50	IM

IM = Intramuscular

8.2 Shipment and Storage

Authorization to ship the Investigational Product to the site will be provided in writing by the sponsor, upon confirmation that all required critical documents for shipment authorization are completed. The Investigational Product will be shipped to the site according to the required storage conditions.

Ad35-GRIN/ENV and placebo are stored at -70°C or below. The different formulations of the Ad35 GRIN/ENV and Ad35-GRIN vaccines and placebo are identified by unique lot numbers. Additionally, all the vials from all the three dosage levels and placebo have a date of manufacturing, storage temperature, dose volume, the name of the manufacturer and the US cautionary statement.

8.3 Dispensing and Handling

The Investigational Product will be dispensed as specified in the Study Operations Manual.

Each vial containing placebo or vaccine should be thawed at ambient temperature in the pharmacy. Thawed vials to be gently swirled to mix the contents and aspirated into a syringe as soon as possible. In case of an unplanned delay, keep the vial at 2-8°C. Vaccine should be utilized as soon as possible. The vaccine should be used within 3 hours post thawing. Draw 0.5 mL of the vaccine into the syringe, label the syringe and transfer to the pharmacist for vaccine administration without delay.

8.4 Administration

Investigational Product will be administered according to the Schedule of Procedures (Appendix A).

The preferred site of first administration is the deltoid muscle of the non-dominant upper arm (for example, injection in the left arm if the volunteer uses mainly the right arm) unless contraindicated for another reason when receiving a single injection of Ad35-GRIN/ENV or Ad35-GRIN or placebo. The booster injection will be injected in the same arm.

Further information on the administration of the Investigational Product is supplied in the Study Operations Manual.

8.5 Accountability and Disposal

All used vials will be returned to the Investigational Product dispenser or pharmacy at the end of each vaccination visit. The date, vial allocation number and location of storage of the returned vials will be recorded.

During the study, the Investigational Product accountability form, the dispensing log and the log of returned vials will be kept and monitored.

At the end of the study, the used and unused vials will be destroyed; destruction will be witnessed, according to IAVI and site specific Standard Operating Procedures.

9.0 ASSESSMENTS

9.1 Safety Assessments

Data on local and systemic reactogenicity will be collected by structured interview, using specific questions. Data on other adverse events will be collected with an open-ended question. All data will be recorded on the appropriate source documents.

9.1.1 Local reactogenicity

The presence of local reactogenicity will be assessed at the time points specified in the Schedule of Procedures (Appendix A).

Local reactogenicity (pain, tenderness, erythema/skin discoloration, skin damage [vesiculation/ulceration], induration, formation of crust or scab) will be assessed and graded using the Appendix B, Adverse Event Severity Assessment Table, as a guideline.

9.1.2 Systemic reactogenicity

The presence of systemic reactogenicity will be assessed at the time points specified in the Schedule of Procedures (Appendix A).

Vital signs (pulse, respiratory rate, blood pressure and temperature) will be measured by study staff prior to vaccination and at least 30 minutes post-vaccination.

Fever, chills, headache, nausea, vomiting, malaise, myalgia and arthralgia will be assessed and graded using the Appendix B, Adverse Event Severity Assessment Table, as a guideline.

9.1.3 Other adverse events

Occurrence of other adverse events (including Serious Adverse Events) will be collected following an open question to volunteers at the time points indicated in the Schedule of Procedures (Appendix A). The adverse events will be graded using the Appendix B, Adverse Event Severity Assessment Table, as a guideline.

For more information regarding adverse events refer to Section 10.0, Adverse Events.

9.1.4 Concomitant Medications

During the study, information regarding concomitant medications and reasons for their use will be solicited from the study volunteers at each visit and recorded.

Concomitant receipt of Investigational Products, including other HIV vaccines is prohibited during the study.

If clinically indicated, non-live vaccines (non-HIV) or live attenuated influenza vaccine may be given up to 14 days before study vaccination(s) or after post-vaccination blood draw (i.e., 2 weeks after study vaccinations).

Live-attenuated vaccines (non-HIV) may be given 60 days before study vaccination(s) or after the post-vaccination blood draw. However, the study vaccination(s) should not be given if there are any continuing symptoms from recently administered non-HIV vaccines. In this situation, the Principal Investigator should consult with the IAVI Medical Monitor before administering the next study vaccination.

The administration of immunoglobulin will be followed by the discontinuation of vaccinations. In this situation, the Principal Investigator should consult with the IAVI Medical Monitor before administering the next study vaccination.

If the use of a short tapering (< 2 weeks) of corticosteroids is required, the study vaccinations may be continued after a 4-week washout period, provided that the medical condition requiring this therapy has completely resolved and, in the opinion of both the site investigator and the IAVI Medical Monitor, the continuation of the study vaccinations will not jeopardize the safety of the volunteer. Volunteers requiring chronic (> 2 weeks) or long term therapy will not receive any further vaccinations but will continue with follow-up visits until the end of the study.

9.1.5 Routine laboratory parameters

Table 3 shows the laboratory parameters that will be measured routinely. These parameters will include hematology, clinical chemistry, immunological assays and urinalysis. The samples for these tests will be collected at the time points indicated in the Schedule of Procedures (Appendix A).

Table 3
Laboratory Parameters

Laboratory Parameter	Test
Hematology	Complete blood count (hemoglobin, hematocrit, erythrocytes, leucocytes, platelets)<br>Differential count, absolute neutrophil count, absolute lymphocyte count
Clinical Chemistry	Liver function tests: aspartate transferase (AST), alanine transferase

	(ALT), creatinine, total and direct bilirubin
Immunology	CD4 and CD8 T-cells (absolute count)
Urinalysis	Dipstick test for protein, blood glucose, ketones, esterase (leukocytes), nitrite.<br>If abnormalities (≥2+ blood, protein, leucocytes) are found on dipstick test then further test will be performed (e.g., microscopy, culture)

9.1.6 Specific screening tests:

Volunteers will be screened to exclude the following diseases:

- Hepatitis B: positive for hepatitis B surface antigen (HbsAg)
- Hepatitis C: positive for hepatitis C antibodies (HCV antibodies)
- Syphilis: confirmed diagnosis of active syphilis (RPR & TPHA or equivalent)

9.2 Immunogenicity Assessments

9.2.1 Antibody Responses

- Antibodies against HIV proteins will be measured at times indicated on the Schedule of Procedures (Appendix A).
- Anti-Ad35 vector neutralizing antibodies will be assessed at times indicated in the Schedule of Procedures (Appendix A).

9.2.2 Cellular Responses

Immunogenicity assays, including ELISPOT for monitoring the number of circulating T-cells that can be stimulated to produce cytokines, will be performed at time points indicated in the Schedule of Procedure (Appendix A, using peptide pools representing all or a portion of the encoded antigen(s). Further characterization of phenotype and functional properties of responding T-cells will be performed using multi-parameter flow cytometry and/or the Virus Inhibition Assay (ability of PBMC to restrict the growth of HIV in vitro). An algorithm may be applied to determine which time points are analyzed.

Further studies may be carried out using a.) Peptide pools designed to determine the specific epitopes recognized and b.) Peptides from different HIV-subtypes and c.) Peptides from Ad35 vector proteins. Selected T-cell responses may be further characterized for HLA restriction and additional markers on the responding cells, such as markers for activation or homing to mucosal tissues.

At each time point indicated in the Schedule of Procedures (Appendix A), using the procedure provided by the IAVI Core Laboratory, vials of frozen peripheral blood mononuclear cells (PBMC) each containing approximately 10^7 PBMC will be taken for immunogenicity analysis (ELISPOT, CFC) and/or quality control assays at the IAVI Core Laboratory. These samples will be shipped promptly, according to an agreed upon schedule, included in the Study Analytical Plan.

9.2.3 PBMC, Serum and Plasma Storage

Samples of cryo-preserved PBMC, plasma and serum will be taken at time points indicated in the Schedule of Procedures (Appendix A) for purposes of standardization, quality control and for future assays related to HIV vaccine research and development. These samples will be archived and only a code will identify the samples.

For the PBMC processing and potentially some immunogenicity assessments, the laboratory personnel will be trained as necessary by the sponsor and provided with a written procedure manual.

The samples described in Sections 9.2.2 and 9.2.3 will be shipped routinely from the site to the IAVI Core Laboratory. The majority of the immunological testing will be performed at the IAVI Core Laboratory in accordance with IAVI standard operating procedures and standard reagents.

9.3 Other Assessments

9.3.1 HLA Typing

Samples for HLA typing will be collected at the time point indicated in the Schedule of Procedures (Appendix A).

HLA typing will be performed on samples for volunteers vaccinated at *each* dosage level, provided that T-cell responses are detected at that dosage level.

9.3.2 HIV test

Samples will be tested at the time points indicated in the Schedule of Procedures (Appendix A). Further information is specified in Section 11.1 HIV Testing.

9.3.3 Pregnancy Test

A urine pregnancy test for all female volunteers will be performed by measurement of Human Chorionic Gonadotrophin (βhCG) at the time points indicated in the Schedule of Procedures (Appendix A).

The results of the pregnancy test must be negative prior to vaccination.

9.3.4 Antibody response to the Ad35 vector

Since pre-existing immunity to Ad35, as well as antibodies induced by Ad35 vector itself, may impair subsequent immune responses to the proteins of interest expressed by the vector, antibodies to the vector will be assessed at time points specified in the Schedule of Procedures

(Appendix A). Samples for anti-Ad35 neutralizing antibodies will be sent to the Core Laboratory or another suitable laboratory for testing.

9.3.5 Viral inhibition assay

Viral inhibition assays will be carried out at IAVI Core Laboratory at time points indicated in the Schedule of Procedures (Appendix A).

9.3.6 Viral shedding

Oropharyngeal swabs and urine specimens will be collected in 9 volunteers in each dose group at time points indicated in the Schedule of Procedures (Appendix A), basically day 0, day 14 post 1st injection and prior to booster injection at month 6. The samples will be frozen and adenovirus culture will be performed if Ad35-specific DNA PCR is found positive. In addition, specimens for adenovirus investigation will be collected as clinically indicated according to the medical judgment of the investigator within the first 14 days post immunization for any reported respiratory or genito-urinary tract or diarrheal illness or conjunctivitis, unless another cause is revealed by the diagnostic investigations (e.g. bacterial UTI, streptococcal pharyngitis, positive test for influenza antigen). .

10.0 ADVERSE EVENTS

10.1 Definition

An adverse event (AE) is any untoward medical occurrence in a volunteer administered Investigational Product; an AE does not necessarily have a causal relationship with the Investigational Product. An AE can therefore be any unfavorable or unintended sign (including an abnormal laboratory finding), symptom, or disease, temporally associated with the use of the Investigational Product whether or not related to the Investigational Product.

10.2 Assessment of Severity of Adverse Events

Assessment of severity of all AEs is ultimately the responsibility of the principal investigator.

The following general criteria should be used in assessing adverse events as mild, moderate or severe at the time of evaluation:

- Mild: Mild discomfort; Minimal or no limitation of daily activities; Medical intervention not required;
- Moderate: Moderate discomfort; Some limitation of daily activities but able to work part-time or full-time with some assistance; May require minimal or no medical intervention;
- Severe: Severe discomfort; Marked limitation of daily activities, unable to work; Requires medical intervention;

- Very severe: Symptoms causing inability to perform basic self-care functions OR Medical or operative intervention indicated to prevent permanent impairment, persistent disability, or death

Guidelines for assessing the severity of specific adverse events and laboratory abnormalities are listed in Appendix B, Adverse Event Severity Assessment Table.

10.3 Relationship to Investigational Product

The relationship of an (S)AE is assessed and determined by the Principal Investigator or designee. All medically indicated and available diagnostic methods (e.g., lab, blood smear, culture, X-ray, etc.) should be used to assess the nature and cause of the AE/SAE. Best clinical and scientific judgment should be used to assess relationship of AE/SAEs to the Investigational Product and/or other cause.

The following should be considered for the assessment of relationship of adverse events to the investigational Product:

- Presence/absence of a clear temporal (time) sequence between administration of the Investigational Product and the onset of AE/SAE

- Presence/absence of another cause that could more likely explain the AE/SAE (concurrent disease, concomitant medication, environmental or toxic factors, etc.)

- Whether or not the AE/SAE follows a known response pattern associated with the investigational Product

The relationship assessment should be reported as one of the following:
Not Related: clearly explained by another cause (concurrent disease, concomitant medication, environmental or toxic factors, etc.).

Unlikely: more likely explained by another cause (concurrent disease, concomitant medication, environmental or toxic factors, etc.).

Possibly: equally likely explained by another cause, but the possibility of the Investigational Product relationship cannot be ruled out (e.g., reasonably well temporally related and/or follows a known Investigational Product response pattern but equally well explained by another cause).

Probably: more likely explained by the investigational Product (e.g., reasonably well temporally related and/or follows a known Investigational Product response pattern and less likely explained by another cause).

Definitely: clearly explained by the Investigational Product

For the purpose of expedited safety reporting, all possibly, probably or definitely related SAEs are considered Investigational Product-related SAEs.

10.4 Serious Adverse Events

An adverse event is reported as a "Serious Adverse Event" if it meets any the following criteria (as per ICH GCP Guidelines):

- Results in death
- Is life threatening
- Results in persistent or significant disability/incapacity.
- Requires in-patient hospitalization or prolongs existing hospitalization.
- Is a congenital anomaly/birth defect or spontaneous abortion.
- Any other important medical condition that requires medical or surgical intervention to prevent permanent impairment of a body function or structure.

Serious Adverse Events (SAEs) should be reported to IAVI within 24 hours of the study staff becoming aware of the event. All SAEs should be sent by fax to a designated fax number or e-mailed to MAreport@iavi.org according to SAE Reporting Guidelines (see Study Operations Manual).

To discuss Investigational Product related SAEs or any urgent medical questions related to the SAE, the site investigator should contact the IAVI medical monitor directly (see the Contact List).

The IAVI SAE Report Form should be completed with all the available information at the time of reporting. The minimum data required in reporting an SAE are the volunteer identification number, date of birth, gender, event description (in as much detail as is known at the time), onset date of event (if available), reason event is classified as Serious, reporting source (name of principal investigator or designee), relationship assessment to the investigational product by the investigator.

The Principal Investigator or designee is required to write a detailed written report with follow up until resolution or until the medical condition is judged by the principal investigator or designee to have stabilized.

The Principal Investigator or designee must notify the local IRB/IEC of all SAEs as appropriate. In case of Investigational Product related SAEs, the sponsor will notify the FDA, the Safety Review Board and other study sites where the same Investigational Product is being tested.

More details on SAE definitions and reporting requirements are provided in the SAE Reporting Guidelines (see Study Operations Manual).

10.5 Clinical Management of Adverse Events

Adverse events (AEs) will be managed by the clinical study team who will assess and treat the volunteer as appropriate, including referral. If any treatment/medical care is required as a result of the harm caused by the Investigational Product or study procedures, this treatment will be provided free of charge.

If a volunteer has an adverse event and/or abnormal laboratory value that is known at the time of study vaccination(s), the specifications of Section 12.1 will be followed.

Volunteers will be followed until the adverse event resolves or stabilizes or up to the end of the study, whichever comes last. If at the end of the study, an adverse event (including clinically significant laboratory abnormality) that is considered possibly, probably or definitely related to the Investigational Product is unresolved, follow-up will continue until resolution if possible and the volunteer will be referred.

10.6 Pregnancy

Although not considered an adverse event, if a female volunteer becomes pregnant during the study, it is the responsibility of the Principal Investigator or designee to report the pregnancy promptly to IAVI using the designated case report forms. However, serious complications of pregnancy that meet SAE criteria specified in the Section 10.4 of this Protocol (e.g., eclampsia, spontaneous abortion, etc.) should be reported as SAEs. For follow up on a pregnancy, refer to Section 12.2.

If a female volunteer becomes pregnant during the study, vaccinations will be discontinued and the volunteer followed until the end of pregnancy. Approximately 2–4 weeks after delivery, the baby will be examined by a physician to assess his/her health status and the results will be reported to IAVI.

10.7 Intercurrent HIV Infection

HIV infection cannot be caused by the Investigational Product. If a volunteer is found to be HIV-infected, study vaccinations must be discontinued and the volunteer followed according to procedures described in Section 12.2.

Intercurrent HIV infection in study volunteers, although not considered an SAE, must be reported promptly to IAVI using the designated case report forms. IAVI will report intercurrent HIV infections to the FDA using the same procedure as SAE reports. However, serious medical conditions associated with the HIV infection that meet SAE criteria specified in the section 10.4 of this Protocol (e.g. sepsis, PCP pneumonia, etc.) should be reported as SAEs using SAE Report Form.

11.0 MANAGEMENT OF HIV ISSUES DURING AND FOLLOWING THE STUDY

11.1 HIV Testing

Only volunteers who are not HIV-infected at screening will be enrolled into the study.

Volunteers will be tested for HIV-1 and HIV-2 antibodies as indicated in the Schedule of Procedures (Appendix A) or as needed, if medical or social circumstances arise.

If the routine post-vaccination HIV Antibody test is positive, a predetermined algorithm will be followed to distinguish an immune response to the vaccine from an HIV infection through exposure in the community.

If a volunteer is found to be HIV-infected, a newly drawn blood specimen will be collected for confirmation.

Volunteers who have a positive HIV-antibody test(s) as a result of vaccine-induced HIV antibodies, rather than a true HIV infection (false positive HIV test), will have their test result reported as "Not infected with HIV-1 or HIV-2" (to prevent unblinding of volunteer and staff) and will be followed up until the test becomes negative. At the end of the study, these volunteers will be offered a continuing follow-up until the test becomes negative.

Should a volunteer require an HIV test outside the study for personal reasons, it is recommended that the volunteer contact the site personnel first. The HIV test can be drawn at the clinical site and then processed at the independent laboratory as above. Written evidence of HIV status (HIV-infected or HIV-uninfected) will be provided upon request.

All volunteers will receive HIV prevention counseling and pre-HIV-test and post-HIV-test counseling as specified in Section 11.3.1 Counseling.

11.2 Social Discrimination as a Result of an Antibody Response to Vaccine

The aim is to minimize the possibility of social discrimination in volunteers (if any) who develop vaccine-induced HIV antibodies and test positive on a diagnostic HIV antibody test. Appropriate diagnostic HIV testing and certification will be provided both during and after the study as needed, according to site procedures.

11.3 HIV infection

Volunteers who are found to be HIV-infected at screening and volunteers who acquire HIV infection during the study will be provided the following:

11.3.1 Counseling

The volunteer will be counseled by the study counselors. The counseling process will assist the volunteer with the following issues:

- Psychological and social implications of HIV infection
- Whom to inform and what to say
- Implications for sexual partners
- Implications for child-bearing

- Avoidance of transmission to others in future

11.3.2 Referral for Support and/or Care

Volunteers will be referred to a patient support centre or institution of his/her choice for a full discussion of the clinical aspects of HIV infection. Referral will be made to a designated physician or centre for discussion of options of treatment of HIV-infection.

If a volunteer becomes HIV-infected during the study, he/she will be referred for medical care.

HIV-infected pregnant women will be referred for prenatal care and to a program for the Prevention of Mother to Child Transmission (PMTCT). The pregnant volunteer will be followed according to timeline as specified in Section 10.6

12.0 DISCONTINUATION OF VACCINATIONS AND/OR WITHDRAWAL FROM STUDY

12.1 Discontinuation of Vaccinations

The Principal Investigator as well as the IAVI Medical Monitor will discuss the circumstances relating to any volunteer discontinuing further vaccinations or being considered for discontinuation or deferring of vaccinations. Volunteers will be discontinued from further vaccination for any of the following reasons:

1. Pregnancy
2. Intercurrent HIV Infection
3. Use of immunoglobulin, systemic corticosteroids, immunosuppressive, antiviral, anticancer, or other medications.
4. A disease or condition or an adverse event that may develop, regardless of relationship to the Investigational Product, if the Principal Investigator or designee is of the opinion that further study vaccinations will jeopardize the safety of the volunteer.
5. Any of the following abnormal laboratory parameters (after possible repeated measurements) that are known at the time of vaccination and discussed with sponsor:
   Hematology
   - Hemoglobin <10.0 g/dL
   - Absolute Neutrophil Count (ANC): ≤ 999/mm^3
   - Absolute Lymphocyte Count (ALC): ≤ 500/mm^3
   - Platelets: ≤ 90,000 ≥ 550,000/ mm^3

   Chemistry
   - Creatinine: > 1.4 x ULN
   - AST: >3.0 x ULN
   - ALT: >3.0 x ULN

Urinalysis: Dipstick 2+ confirmed by microscopy for

- Protein
- Blood (not due to menses)
- Leukocytes

6. Receipt of live-attenuated vaccine within the previous 60 days (live attenuated flu vaccine within 14 days) or planned receipt within 60 days after vaccination with Investigational Product or receipt of other vaccine within the previous 14 days or planned receipt within 14 days after vaccination with Investigational Product.
7. A severe local reaction involving the major part of the injected arm circumference.
8. Anaphylaxis; bronchospasm; laryngeal oedema; convulsions or encephalopathy following study vaccinations.
9. Life-threatening adverse event following study vaccinations, unless not related to the Investigational Product and fully resolved.
10. Any immediate hypersensitivity reaction judged to be definitely related to the Investigational Product.
11. Volunteer request to discontinue further vaccination.
12. Participating in another clinical study of an Investigational Product.

12.2 Follow-up after Discontinuation of Further Vaccinations

Volunteers in whom study vaccinations are discontinued due to adverse events will be followed until the adverse event resolves or stabilizes or up to the end of the study, whichever comes last. These volunteers will not be replaced.

Follow-up of HIV-infected individuals who have received Investigational Product will be determined by the Principal Investigator and the IAVI Medical Monitor.

Follow-up of pregnant volunteers will be done as specified in Section 10.6.

12.3 Withdrawal from the Study (Early Termination)

Volunteers may be withdrawn from the study permanently for the following reasons:

1. Volunteers may withdraw from the study at any time if they wish, for any reason.
2. The principal investigator or designee has reason to believe that the volunteer is not complying with the protocol.
3. If the sponsor decides to terminate or suspend the study.

12.4 Follow-up Withdrawal from the Study (Early Termination)

If the volunteer withdraws from the study, all termination visit procedures will be performed according to the Schedule of Procedures (Appendix A) if possible.

Every effort will be made to determine and document the reason for withdrawal from the study.

13.0 DATA HANDLING

13.1 Data Collection and Record Keeping at the Study Site

Data Collection: All study data will be collected by the clinical study staff using designated source documents and entered onto the appropriate case report forms (CRFs). CRFs will be provided by IAVI and should be handled in accordance with the instructions from IAVI. All study data must be verifiable to the source documentation. A file will be held for each volunteer at the clinic(s) containing all the source documentation. Source documentation will be available for review to ensure that the collected data are consistent with the CRFs.

All CRFs and laboratory reports will be reviewed by the clinical team, who will ensure that they are accurate and complete.

Source documents and other supporting documents will be kept in a secure location and remain separate from volunteer identification information (name, address, etc.) to ensure confidentiality.

Standard GCP practices will be followed to ensure accurate, reliable and consistent data collection.

Source documents include but are not limited to:

- Signed Informed Consent Documents
- Dates of visits including dates of vaccinations
- Documentation of any existing conditions or past conditions relevant to eligibility
- Reported laboratory results
- All adverse events
- Concomitant medications
- Local and systemic reactogenicity

13.2 Data Collection and Transfer at the IAVI Core Laboratory

Data generated at the IAVI Core laboratory will be transferred directly to the Data Coordinating Centre.

13.3 Data Entry at the Study Site

The data collected at the site will be entered onto the electronic CRFs by the designated study staff. To provide for real time assessment of safety, data should be entered as soon as reasonably feasible following a study visit.

14.0 STATISTICAL CONSIDERATIONS

The statistician at the Data Coordinating Centre (EMMES Corporation), in collaboration with the sponsor and the principal investigator (or designee), will create tables according to a data analysis plan that has been reviewed and agreed to by the principal investigator (or designee). The EMMES Corporation will conduct the data analysis and will provide interim and final study reports for the principal investigator (or designee), the sponsor and the regulatory authorities, as appropriate. Prior to an analysis, additional monitoring visits will take place if necessary to validate the data held on the database, as well as all consent forms and dispensing records. Data files will be prepared by EMMES from a 'frozen' dataset for that particular analysis.

14.1 Sample Size

A total of 56 volunteers (40 Vaccine/16 placebo) will be entered into each of the 4 groups (10 vaccine/ 4 placebo recipients in each group) scheduled to receive Ad35-GRIN/ENV or Ad35-GRIN vaccines or placebo. All injections will be intramuscular (IM). An over-enrolment of about 10% (1-2 volunteers per arm) will be permitted to facilitate prompt enrolment.

14.2 Statistical Power and Analysis

Safety will be assessed by analyses of the following primary end-points (events), where the unit of analysis in each case will be the proportion of volunteers with at least one event:

a) Severe and very severe local reactogenicity events
b) Severe and very severe systemic reactogenicity events
c) Severe unsolicited adverse events, including severe and very severe laboratory abnormalities
d) Severe unsolicited adverse events that are possibly, probably or definitely related to vaccine or placebo, including any severe and very severe laboratory abnormalities
e) Serious adverse events
f) Mild or moderate local or systemic reactogenicity events, or adverse events

The rate of Serious Adverse Events related to the Investigational Product will be used as one measure of the safety of the Investigational Product. Adverse Events that may be temporarily incapacitating (for example, loss or cancellation of work or social activities), which could make an Investigational Product impractical for large scale use if they occur in more than a small proportion of cases, will also be assessed.

All adverse events will be reported, grouped as to whether or not they qualify as SAEs, their severity assessment, and their relationship to the Investigational Product (as judged by the investigator).

Cellular immune responses will be analyzed using binomial methods to examine for the presence or absence of HIV specific T-cell responses quantified by

ELISPOT and cytokine flow cytometry (CFC). Assays will be performed using the IAVI Core Laboratory SOPs and standard reagents for all volunteers.

Presence or absence of antibodies to HIV proteins will be also analyzed. Assays will be performed in a similar fashion in all volunteers.

Ad35-specific neutralizing antibodies will be analyzed post vaccination (percentage of volunteers positive and mean titer).

Based on the previous experience with IAVI Phase I Investigational Product studies, it is expected that the amount of missing, unused or spurious data will be insignificant. Unused and spurious data will be listed separately and excluded from the statistical analysis. Missing data will be excluded from the statistical analysis.

Prior to enrolment of the mid-dose group:
Prior to enrolment into the mid-dose group, the SRB will review the safety of the low dose, based on a compilation of blinded data from the first 9 volunteers enrolled. Any additional subsequent severe or very severe events will be provided to the SRB as an update prior to proceeding with the next dosage level. Based on the binomial distribution, if none of the 9 volunteers experiences an event, then the upper 95% confidence limit (CL) for the rate of these adverse events in the population is 33.6%. Similarly, if one or two events are observed then the corresponding upper 95% CLs are 48.2% and 60.0%, respectively.

Prior to enrolment of the high-dose group:
Enrolment into the high dose group (Groups C) will depend on the review of the safety data of the mid-dose group, following the procedure described above.

Final analysis:

Vaccine versus Placebo within a dose group
For comparison of active vaccine (N=10) versus placebo (N=4) within the same dosage group, there will be 80% power to detect a statistically significant ($p<0.05$) difference of 76% if the event rate in the placebo group is 1% to 5%, based on Fisher's exact 1-tailed test. This power will apply to a comparison of the anti-GRIN responses in Ad35-GRIN and AD35-GRIN/ENV high dose group.

Overall Vaccine versus Placebo
For comparison of active vaccine (N=40) versus placebo (N=16), there will be 80% power to detect a statistically significant ($p<0.05$) difference of 28.5% and 32.7% if the event rate in the placebo group is 1% or 5%, respectively, based on Fisher's exact 1-tailed test.

Comparison of Active Vaccine groups
With 10 volunteers receiving active vaccine in each dose group, if the rate of events in one group is 5%, 10%, 20% or 30%, then there will be 80% power to detect statistically significant ($p<0.05$) differences of about 65%, 67%, 68% and 65%, respectively, based on Fisher's exact 2-tailed test.

15.0 QUALITY CONTROL AND QUALITY ASSURANCE

To ensure the quality and reliability of the data gathered and the ethical conduct of this study, a Study Operations Manual has been developed.

Regular monitoring will be performed according to ICH-GCP as indicated in Section 17.3.

An independent audit of the study may be performed, if appropriate, at the discretion of the sponsor.

By signing the protocol, the Principal Investigator, agrees to facilitate study related monitoring, audits, IRB/IEC review and regulatory inspection(s) and direct access to source documents. Such information will be treated as strictly confidential and under no circumstances be made publicly available.

16.0 DATA AND BIOLOGICAL MATERIAL

All data and all biological material collected throughout the clinical trial shall be the joint property of the investigators and IAVI and managed in accordance with the Clinical Trial Agreement. Distribution and use of these data will be conducted by agreement of both parties.

The computerized raw data generated will be held by the DCC on behalf of the sponsor. The study site will also hold the final data files and tables generated for the purpose of analysis. The Principal Investigator or designees will have access to the clinical study database with appropriate blinding.

17.0 ADMINISTRATIVE STRUCTURE

The Principal Investigator will be responsible for all aspects of the study at the study site.

17.1 Safety Review Board

The SRB will review blinded safety data and make recommendations regarding the dose-escalation. The SRB will also be convened to consider any significant safety issue which arises during the study. The SRB will consist of independent clinicians/scientists/statisticians who are not involved in the study. Investigators responsible for the clinical care of volunteers or representative of the sponsor may not be members of the SRB.

However, the SRB may invite the Principal Investigator or designee and a sponsor representative to an open session of the meeting to provide information on study conduct, present data or to respond to questions.

The review of study data by the SRB will take place at a pre-determined interval or may be specifically requested (see Section 17.1.2 Indications for Discontinuation of Vaccinations in all Volunteers).

17.1.1 Content of Interim Review

The SRB will be asked to review the following data:

- All moderate, severe and very severe clinical adverse events/reactogenicity judged by the Principal Investigator or designee to be possibly, probably or definitely related to the Investigational Product, or

- All moderate, severe and very severe laboratory adverse events confirmed on retest and judged by the Principal Investigator or designee to be possibly, probably, or definitely related to Investigational Product.

- All Serious Adverse Events, independent of relationship to the Investigational Product.

- All available safety data prior to the administration of the 6-month booster dose.

The SRB will then recommend to the Principal Investigator and the Sponsor whether or not to escalate the dose.

17.1.2 Indications for Discontinuation of Vaccinations in all Volunteers

If 3 or more of the volunteers participating in this study develop an SAE judged definitely, probably or possibly related to the Investigational Product, the Principal Investigator or designee and the sponsor will request a review by the SRB. The study will be suspended pending a review of all safety data by the SRB. The study may be unblinded at the discretion of the SRB.

Following this review, the SRB will make a recommendation to the Sponsor and the Principal Investigator regarding the continuation of the study.

17.2 Study Supervision

The Principal Investigator, the IAVI Chief Medical Officer, the Medical Monitor and the Senior Clinical Program Manager will be provided progress report(s) of this study. Close cooperation will be necessary to track study progress, respond to queries about proper study implementation and management, address issues in a timely manner, and assure consistent documentation, and effective information sharing. Rates of accrual, retention, and other parameters relevant to the site's performance will be regularly and closely monitored by the study team, as well as the SRB.

17.3 Study Monitoring

On-site monitoring will be conducted to ensure that the study is conducted in compliance with human subjects and other research regulations and guidelines, recorded and reported in accordance with the protocol, is consistent with locally accepted HIV counseling practices, standard operating procedures, Good Clinical Practice (GCP) and applicable regulatory requirements.

The monitor will confirm the quality and accuracy of data at the site by validation against the source documents, such as clinical records, and against the database when applicable. The Investigators and volunteers, by giving consent, agree that the monitor may inspect study facilities and source records (e.g., informed consent forms, clinic and laboratory records, other source documents), as well as observe the performance of study procedures. Such information will be treated as strictly confidential and will under no circumstances be made publicly available.

The monitoring will adhere to Good Clinical Practice guidelines. The Principal Investigator will permit inspection of the facilities and all study related documentation by authorized representatives of IAVI, and Government and Regulatory Authorities relevant to this study.

17.4 Investigator's Records

Study records include administrative documentation—including reports and correspondence relating to the study—as well as documentation related to each volunteer screened for and/or enrolled in the study—including informed consent forms, case report forms, and all other source documents. The investigator will maintain and store, in a secure manner, complete, accurate, and current study records for a minimum of 2 years after marketing application approval or the study is discontinued and applicable national and local health authorities are notified. IAVI will notify the Principal Investigator of these events.

18.0 INDEMNITY

The Sponsor and Institution are responsible to have appropriate liability insurance. For research-related injuries and/or medical problems determined to result from receiving the Investigational Product, treatment including necessary emergency treatment and proper follow-up care will be made available to the volunteer free of charge at the expense of the Sponsor.

19.0 PUBLICATION

A primary manuscript describing safety and immune responses in this trial will be prepared promptly after the data analysis is available, based on the data compiled by the IAVI statistical centre. Authors will be representatives of the trial site, the statistical centre, the laboratories and IAVI, subject to the generally accepted criteria of contributions to the design, work, analysis and writing of the study. Manuscripts will be reviewed by representatives of each participating group as specified in the Clinical Trial Agreement.

20.0 ETHICAL CONSIDERATIONS

The Principal Investigator will ensure that the study is conducted in compliance with the protocol, Standard Operating Procedures in accordance with guidelines laid down by the International Conference on Harmonisation for Good Clinical Practice in clinical studies, the ethical principles that have their origins in the Declaration of Helsinki and applicable regulatory requirements.

APPENDIX A: SCHEDULE OF PROCEDURES

	Screen																Final visit/ET[A]
Study Month		**M0**				**M1**	**M3**	**M6**				**M7**	**M8**	**M9**	**M13**	**M16**	**M18**
Study Week		0		W1	W2	W4	W12	W24		W25	W26	W28	W32	W38	W52	W64	W72
Study Day		0	D3	D7	D14	D28	D84	D168	D171	D175	D182	D197	D224	D266	D364	D448	D504
Visit Windows (Days)		-90/-42[3]	± 1	± 2	± 2	± 3	± 3	± 7[2]	± 1	± 2	± 2	±2	± 7[2]	± 7[2]	± 7[2]	± 7[2]	± 7[2]
Investigational Product/Placebo		**X**						**X**									
Local and Systemic Reactogenicity Assessment (pre-and post vaccination)		X X[1]	X	X	X			XX[1]	X	X	X						
Informed Consent	X																
Screening Questionnaire	X																
Medical History (including concomitant medications)	X	X	X	X	X	X	X	X	X	X	X	X	X	X	X	X	X
General Physical Exam	X																X
Directed Physical Exam		X	X	X	X	X	X	X	X	X	X	X	X	X	X	X	
Adverse Events		X	X	X	X	X	X	X	X	X	X	X	X	X			
Serious Adverse Events		X	X	X	X	X	X	X	X	X	X	X	X	X	X	X	X
Vital Signs (pre-and post vaccination)		X X	X					XX	X								
Hep HBsAg, Hep C, active syphilis	X																
Haematology (+ plasma storage)	X	X		X	X		X	X		X	X			X			X
Clinical Chemistry (+ serum storage)	X	X		X	X		X	X		X	X			X			X
Immunology (CD4, CD8)		X			X			X			X				X		X
Urinalysis	X	X			X			X			X						X
HIV test Pre-/Post HIV-test Counselling	X	X				X		X					X		X		X

	Screen																Final visit/ET[A]
Study Month		M0				M1	M3	M6				M7	M8	M9	M13	M16	M18
Study Week		0		W1	W2	W4	W12	W24		W25	W26	W28	W32	W38	W52	W64	W72
Study Day		0	D3	D7	D14	D28	D84	D168	D171	D175	D182	D197	D224	D266	D364	D448	D504
Visit Windows (Days)		-90/-42[3]	± 1	± 2	± 2	± 3	± 3	± 7[2]	± 1	± 2	± 2	±2	± 7[2]	± 7[2]	± 7[2]	± 7[2]	± 7[2]
Investigational Product/Placebo		**X**						**X**									
Family planning counselling	X	X						X					X		X		
Pregnancy Test (all female volunteers)	X	X						X									X
PBMCs for Cellular immunogenicity assays (ELISPOT assay and CFC)<br>+ PBMC storage		X			X	X		X		X	X	X	X	X	[X]	[X]	[X]
Ad35 Antibody Assay	X					X		X			X			[X]		[X]	[X]
Viral Inhibition Assay		X			X	X					X	X		[X]	[X]	[X]	[X]
Antibody Immunogenicity Assays		X			X			X		X	X		X	X	[X]	[X]	[X]
Sample HLA Typing		X															
Viral Shedding* (oropharyngeal swabs+ urine specimen)		X	X		X			X									
Blood Volume (visit/total)	20/20	85/105	0/105	20/125	85/210	70/280	20/300	85/385	0/385	70/455	85/540	75/615	75/690	80/770	80/850	70/920	85/1005

ET = Early Termination
[1]Taken 30 minutes post injection.
[2] Preferred window; Allowable window is ± 14 day
[3]Allowable window for screening for Ad35 antibodies is - 90 days; all other screening procedures is - 42 days
[X] Performed only if previous test positive
* Only for first 9 volunteers of each dose group

APPENDIX B: ADVERSE EVENT GRADING TOXICITY TABLE

ADAPTED FROM: Division of AIDS table for grading the severity of adult adverse events
Version 1.0, December, 2004

CLINICAL				
**PARAMETER**	**GRADE 1**<br>**Mild**	**GRADE 2**<br>**Moderate**	**GRADE 3**<br>**Severe**	**GRADE 4**<br>**Very Severe**
**ESTIMATING SEVERITY GRADE**				
Clinical adverse event NOT identified elsewhere in this DAIDS AE grading table	Symptoms causing no or minimal interference with usual social & functional activities	Symptoms causing greater than minimal interference with usual social & functional activities	Symptoms causing inability to perform usual social & functional activities	Symptoms causing inability to perform basic self-care functions OR Medical or operative intervention indicated to prevent permanent impairment, persistent disability, or death
**SYSTEMIC**				
Acute systemic allergic reaction	Localized urticaria (wheals) with no medical intervention indicated	Localized urticaria with medical intervention indicated OR Mild angioedema with no medical intervention indicated	Generalized urticaria OR Angioedema with medical intervention indicated OR Symptomatic mild bronchospasm	Acute anaphylaxis OR Life-threatening bronchospasm OR laryngeal edema
Chills	Symptoms causing no or minimal interference with usual social & functional activities	Symptoms causing greater than minimal interference with usual social & functional activities	Symptoms causing inability to perform usual social & functional activities	NA
Fatigue<br>Malaise	Symptoms causing no or minimal interference with usual social & functional activities	Symptoms causing greater than minimal interference with usual social & functional activities	Symptoms causing inability to perform usual social & functional activities	Incapacitating fatigue/ malaise symptoms causing inability to perform basic self-care functions
Fever (nonaxillary)	37.7 – 38.6°C	38.7 – 39.3°C	39.4 – 40.5°C	> 40.5°C
Pain (indicate body site)<br>DO NOT use for pain due to injection (See Injection Site Reactions: Injection site pain)	Pain causing no or minimal interference with usual social & functional activities	Pain causing greater than minimal interference with usual social & functional activities	Pain causing inability to perform usual social & functional activities	Disabling pain causing inability to perform basic self-care functions OR Hospitalization (other than emergency room visit) indicated

CLINICAL				
**PARAMETER**	**GRADE 1**<br>**Mild**	**GRADE 2**<br>**Moderate**	**GRADE 3**<br>**Severe**	**GRADE 4**<br>**Very Severe**
Unintentional weight loss	NA	5 – 9% loss in body weight from baseline	10 – 19% loss in body weight from baseline	≥ 20% loss in body weight from baseline OR Aggressive intervention indicated [e.g., tube feeding or total parenteral nutrition (TPN)]
**INFECTION**				
Infection (any other than HIV infection)	Localized, no systemic antimicrobial treatment indicated AND Symptoms causing no or minimal interference with usual social & functional activities	Systemic antimicrobial treatment indicated OR Symptoms causing greater than minimal interference with usual social & functional activities	Systemic antimicrobial treatment indicated AND Symptoms causing inability to perform usual social & functional activities OR Operative intervention (other than simple incision and drainage) indicated	Life-threatening consequences (e.g., septic shock)
**INJECTION SITE REACTIONS**				
Injection site pain (pain without touching)<br>Or<br>Tenderness (pain when area is touched)	Pain/tenderness causing no or minimal limitation of use of limb	Pain/tenderness limiting use of limb OR Pain/tenderness causing greater than minimal interference with usual social & functional activities	Pain/tenderness causing inability to perform usual social & functional activities	Pain/tenderness causing inability to perform basic self-care function OR Hospitalization (other than emergency room visit) indicated for management of pain/tenderness
Injection site reaction (localized)				
**Adult**	Erythema OR Induration of 5x5 cm – 9x9 cm (or 25 cm^2 – 81cm^2)	Erythema OR Induration OR Edema > 9 cm any diameter (or > 81 cm^2)	Ulceration OR Secondary infection OR Phlebitis OR Sterile abscess OR Drainage	Necrosis (involving dermis and deeper tissue)
Pruritis associated with injection<br>See also Skin: Pruritis (itching - no skin lesions)	Itching localized to injection site AND Relieved spontaneously or with < 48 hours treatment	Itching beyond the injection site but not generalized OR Itching localized to injection site requiring ≥ 48 hours treatment	Generalized itching causing inability to perform usual social & functional activities	NA
**SKIN – DERMATOLOGICAL**				
Alopecia	Thinning detectable by study participant (or by caregiver for young children and disabled adults)	Thinning or patchy hair loss detectable by health care provider	Complete hair loss	NA

CLINICAL				
**PARAMETER**	**GRADE 1**<br>**Mild**	**GRADE 2**<br>**Moderate**	**GRADE 3**<br>**Severe**	**GRADE 4**<br>**Very Severe**
Cutaneous reaction – rash	Localized macular rash	Diffuse macular, maculopapular, or morbilliform rash OR Target lesions	Diffuse macular, maculopapular, or morbilliform rash with vesicles or limited number of bullae OR Superficial ulcerations of mucous membrane limited to one site	Extensive or generalized bullous lesions OR Stevens-Johnson syndrome OR Ulceration of mucous membrane involving two or more distinct mucosal sites OR Toxic epidermal necrolysis (TEN)
Hyperpigmentation	Slight or localized	Marked or generalized	NA	NA
Hypopigmentation	Slight or localized	Marked or generalized	NA	NA
Pruritis (itching – no skin lesions)<br>(See also Injection Site Reactions: Pruritis associated with injection)	Itching causing no or minimal interference with usual social & functional activities	Itching causing greater than minimal interference with usual social & functional activities	Itching causing inability to perform usual social & functional activities	NA
**CARDIOVASCULAR**				
Cardiac arrhythmia (general)<br>(By ECG or physical exam)	Asymptomatic AND No intervention indicated	Asymptomatic AND Non-urgent medical intervention indicated	Symptomatic, non-life-threatening AND Non-urgent medical intervention indicated	Life-threatening arrhythmia OR Urgent intervention indicated
Cardiac-ischemia/infarction	NA	NA	Symptomatic ischemia (stable angina) OR Testing consistent with ischemia	Unstable angina OR Acute myocardial infarction
Hemorrhage (significant acute blood loss)	NA	Symptomatic AND No transfusion indicated	Symptomatic AND Transfusion of ≤ 2 units packed RBCs (for children ≤ 10 cc/kg) indicated	Life-threatening hypotension OR Transfusion of > 2 units packed RBCs (for children > 10 cc/kg) indicated
Hypertension				
**Adult**<br>(with repeat testing at same visit)	> 140 – 159 mmHg systolic<br>OR<br>> 90 – 99 mmHg diastolic	> 160 – 179 mmHg systolic<br>OR<br>> 100 – 109 mmHg diastolic	> 180 mmHg systolic<br>OR<br>> 110 mmHg diastolic	Life-threatening consequences (e.g., malignant hypertension) OR Hospitalization indicated (other than emergency room visit)

CLINICAL				
**PARAMETER**	**GRADE 1**<br>**Mild**	**GRADE 2**<br>**Moderate**	**GRADE 3**<br>**Severe**	**GRADE 4**<br>**Very Severe**
Hypotension	NA	Symptomatic, corrected with oral fluid replacement	Symptomatic, IV fluids indicated	Shock requiring use of vasopressors or mechanical assistance to maintain blood pressure
Pericardial effusion	Asymptomatic, small effusion requiring no intervention	Asymptomatic, moderate or larger effusion requiring no intervention	Effusion with non-life threatening physiologic consequences OR Effusion with non-urgent intervention indicated	Life-threatening consequences (e.g., tamponade) OR Urgent intervention indicated
Prolonged PR interval				
**Adult**	PR interval<br>0.21 – 0.25 sec	PR interval<br>> 0.25 sec	Type II 2nd degree AV block OR Ventricular pause > 3.0 sec	Complete AV block
Prolonged QTc				
**Adult**	Asymptomatic, QTc interval 0.45 – 0.47 sec OR Increase interval < 0.03 sec above baseline	Asymptomatic, QTc interval 0.48 – 0.49 sec OR Increase in interval 0.03 – 0.05 sec above baseline	Asymptomatic, QTc interval ≥ 0.50 sec OR Increase in interval ≥ 0.06 sec above baseline	Life-threatening consequences, e.g. Torsade de pointes or other associated serious ventricular dysrhythmia
Thrombosis/embolism	NA	Deep vein thrombosis AND No intervention indicated (e.g., anticoagulation, lysis filter, invasive procedure)	Deep vein thrombosis AND Intervention indicated (e.g., anticoagulation, lysis filter, invasive procedure)	Embolic event (e.g., pulmonary embolism, life-threatening thrombus)
Vasovagal episode (associated with a procedure of any kind)	Present without loss of consciousness	Present with transient loss of consciousness	NA	NA
Ventricular dysfunction (congestive heart failure)	NA	Asymptomatic diagnostic finding AND intervention indicated	New onset with symptoms OR Worsening symptomatic congestive heart failure	Life-threatening congestive heart failure

CLINICAL				
**PARAMETER**	**GRADE 1**<br>**Mild**	**GRADE 2**<br>**Moderate**	**GRADE 3**<br>**Severe**	**GRADE 4**<br>**Very Severe**
**GASTROINTESTINAL**				
Anorexia	Loss of appetite without decreased oral intake	Loss of appetite associated with decreased oral intake without significant weight loss	Loss of appetite associated with significant weight loss	Life-threatening consequences OR Aggressive intervention indicated [e.g., tube feeding or total parenteral nutrition (TPN)]
Ascites	Asymptomatic	Symptomatic AND Intervention indicated (e.g., diuretics or therapeutic paracentesis)	Symptomatic despite intervention	Life-threatening consequences
Cholecystitis	NA	Symptomatic AND Medical intervention indicated	Radiologic, endoscopic, or operative intervention indicated	Life-threatening consequences (e.g., sepsis or perforation)
Constipation	NA	Persistent constipation requiring regular use of dietary modifications, laxatives, or enemas	Obstipation with manual evacuation indicated	Life-threatening consequences (e.g., obstruction)
Diarrhea				
**Adult**	Transient or intermittent episodes of unformed stools OR Increase of ≤ 3 stools over baseline per 24-hour period	Persistent episodes of unformed to watery stools OR Increase of 4 – 6 stools over baseline per 24-hour period	Bloody diarrhea OR Increase of ≥ 7 stools per 24-hour period OR IV fluid replacement indicated	Life-threatening consequences (e.g., hypotensive shock)
Dysphagia-Odynophagia	Symptomatic but able to eat usual diet	Symptoms causing altered dietary intake without medical intervention indicated	Symptoms causing severely altered dietary intake with medical intervention indicated	Life-threatening reduction in oral intake
Mucositis/stomatitis (clinical exam)<br>Indicate site (e.g., larynx, oral)	Erythema of the mucosa	Patchy pseudomembranes or ulcerations	Confluent pseudomembranes or ulcerations OR Mucosal bleeding with minor trauma	Tissue necrosis OR Diffuse spontaneous mucosal bleeding OR Life-threatening consequences (e.g., aspiration, choking)
Nausea	Transient (< 24 hours) or intermittent nausea with no or minimal interference with oral intake	Persistent nausea resulting in decreased oral intake for 24 – 48 hours	Persistent nausea resulting in minimal oral intake for > 48 hours OR Aggressive rehydration indicated (e.g., IV fluids)	Life-threatening consequences (e.g., hypotensive shock)

CLINICAL				
**PARAMETER**	**GRADE 1 Mild**	**GRADE 2 Moderate**	**GRADE 3 Severe**	**GRADE 4 Very Severe**
Pancreatitis	NA	Symptomatic AND Hospitalization not indicated (other than emergency room visit)	Symptomatic AND Hospitalization indicated (other than emergency room visit)	Life-threatening consequences (e.g., circulatory failure, hemorrhage, sepsis)
Proctitis (functional-symptomatic)	Rectal discomfort AND No intervention indicated	Symptoms causing greater than minimal interference with usual social & functional activities OR Medical intervention indicated	Symptoms causing inability to perform usual social & functional activities OR Operative intervention indicated	Life-threatening consequences (e.g., perforation)
Vomiting	Transient or intermittent vomiting with no or minimal interference with oral intake	Frequent episodes of vomiting with no or mild dehydration	Persistent vomiting resulting in orthostatic hypotension OR Aggressive rehydration indicated (e.g., IV fluids)	Life-threatening consequences (e.g., hypotensive shock)
**NEUROLOGIC**				
Alteration in personality-behavior or in mood (e.g., agitation, anxiety, depression, mania, psychosis)	Alteration causing no or minimal interference with usual social & functional activities	Alteration causing greater than minimal interference with usual social & functional activities	Alteration causing inability to perform usual social & functional activities	Behavior potentially harmful to self or others (e.g., suicidal and homicidal ideation or attempt, acute psychosis) OR Causing inability to perform basic self-care functions
Altered Mental Status<br>For Dementia, see Cognitive and behavioral/attentional disturbance (including dementia and attention deficit disorder)	Changes causing no or minimal interference with usual social & functional activities	Mild lethargy or somnolence causing greater than minimal interference with usual social & functional activities	Confusion, memory impairment, lethargy, or somnolence causing inability to perform usual social & functional activities	Delirium OR obtundation, OR coma
Ataxia	Asymptomatic ataxia detectable on exam OR Minimal ataxia causing no or minimal interference with usual social & functional activities	Symptomatic ataxia causing greater than minimal interference with usual social & functional activities	Symptomatic ataxia causing inability to perform usual social & functional activities	Disabling ataxia causing inability to perform basic self-care functions

CLINICAL				
**PARAMETER**	**GRADE 1 Mild**	**GRADE 2 Moderate**	**GRADE 3 Severe**	**GRADE 4 Very Severe**
Cognitive and behavioral/attentional disturbance (including dementia and attention deficit disorder)	Disability causing no or minimal interference with usual social & functional activities OR Specialized resources not indicated	Disability causing greater than minimal interference with usual social & functional activities OR Specialized resources on part-time basis indicated	Disability causing inability to perform usual social & functional activities OR Specialized resources on a full-time basis indicated	Disability causing inability to perform basic self-care functions OR Institutionalization indicated
CNS ischemia (acute)	NA	NA	Transient ischemic attack	Cerebral vascular accident (CVA, stroke) with neurological deficit
Headache	Symptoms causing no or minimal interference with usual social & functional activities	Symptoms causing greater than minimal interference with usual social & functional activities	Symptoms causing inability to perform usual social & functional activities	Symptoms causing inability to perform basic self-care functions OR Hospitalization indicated (other than emergency room visit) OR Headache with significant impairment of alertness or other neurologic function
Insomnia	NA	Difficulty sleeping causing greater than minimal interference with usual social & functional activities	Difficulty sleeping causing inability to perform usual social & functional activities	Disabling insomnia causing inability to perform basic self-care functions
Neuromuscular weakness (including myopathy & neuropathy)	Asymptomatic with decreased strength on exam OR Minimal muscle weakness causing no or minimal interference with usual social & functional activities	Muscle weakness causing greater than minimal interference with usual social & functional activities	Muscle weakness causing inability to perform usual social & functional activities	Disabling muscle weakness causing inability to perform basic self-care functions OR Respiratory muscle weakness impairing ventilation
Neurosensory alteration (including paresthesia and painful neuropathy)	Asymptomatic with sensory alteration on exam or minimal paresthesia causing no or minimal interference with usual social & functional activities	Sensory alteration or paresthesia causing greater than minimal interference with usual social & functional activities	Sensory alteration or paresthesia causing inability to perform usual social & functional activities	Disabling sensory alteration or paresthesia causing inability to perform basic self-care functions

CLINICAL				
**PARAMETER**	**GRADE 1**<br>**Mild**	**GRADE 2**<br>**Moderate**	**GRADE 3**<br>**Severe**	**GRADE 4**<br>**Very Severe**
Seizure: (new onset)<br>**Adult**	NA	1 seizure	2 – 4 seizures	Seizures of any kind which are prolonged, repetitive (e.g., status epilepticus), or difficult to control (e.g., refractory epilepsy)
Seizure: (known pre-existing seizure disorder)<br>**– Adult**<br>For worsening of existing epilepsy the grades should be based on an increase from previous level of control to any of these levels.	NA	Increased frequency of pre-existing seizures (non-repetitive) without change in seizure character OR Infrequent break-through seizures while on stable medication in a previously controlled seizure disorder	Change in seizure character from baseline either in duration or quality (e.g., severity or focality)	Seizures of any kind which are prolonged, repetitive (e.g., status epilepticus), or difficult to control (e.g., refractory epilepsy)
Syncope (not associated with a procedure)	NA	Present	NA	NA
Vertigo	Vertigo causing no or minimal interference with usual social & functional activities	Vertigo causing greater than minimal interference with usual social & functional activities	Vertigo causing inability to perform usual social & functional activities	Disabling vertigo causing inability to perform basic self-care functions
**RESPIRATORY**				
Bronchospasm (acute)	FEV1 or peak flow reduced to<br>70 – 80%	FEV1 or peak flow<br>50 – 69%	FEV1 or peak flow<br>25 – 49%	Cyanosis OR FEV1 or peak flow < 25% OR Intubation
Dyspnea or respiratory distress				
**Adult**	Dyspnea on exertion with no or minimal interference with usual social & functional activities	Dyspnea on exertion causing greater than minimal interference with usual social & functional activities	Dyspnea at rest causing inability to perform usual social & functional activities	Respiratory failure with ventilatory support indicated
**MUSCULOSKELETAL**				
Arthralgia	Joint pain causing no or minimal interference with usual social & functional activities	Joint pain causing greater than minimal interference with usual social & functional activities	Joint pain causing inability to perform usual social & functional activities	Disabling joint pain causing inability to perform basic self-care functions

CLINICAL				
**PARAMETER**	**GRADE 1 Mild**	**GRADE 2 Moderate**	**GRADE 3 Severe**	**GRADE 4 Very Severe**
Arthritis	Stiffness or joint swelling causing no or minimal interference with usual social & functional activities	Stiffness or joint swelling causing greater than minimal interference with usual social & functional activities	Stiffness or joint swelling causing inability to perform usual social & functional activities	Disabling joint stiffness or swelling causing inability to perform basic self-care functions
Bone Mineral Loss				
**Adult**	BMD t-score -2.5 to -1.0	BMD t-score < -2.5	Pathological fracture (including loss of vertebral height)	Pathologic fracture causing life-threatening consequences
Myalgia (non-injection site)	Muscle pain causing no or minimal interference with usual social & functional activities	Muscle pain causing greater than minimal interference with usual social & functional activities	Muscle pain causing inability to perform usual social & functional activities	Disabling muscle pain causing inability to perform basic self-care functions
Osteonecrosis	NA	Asymptomatic with radiographic findings AND No operative intervention indicated	Symptomatic bone pain with radiographic findings OR Operative intervention indicated	Disabling bone pain with radiographic findings causing inability to perform basic self-care functions
**GENITOURINARY**				
Inter-menstrual bleeding (IMB)	Spotting observed by participant OR Minimal blood observed during clinical or colposcopic examination	Inter-menstrual bleeding not greater in duration or amount than usual menstrual cycle	Inter-menstrual bleeding greater in duration or amount than usual menstrual cycle	Hemorrhage with life-threatening hypotension OR Operative intervention indicated
Urinary tract obstruction (e.g., stone)	NA	Signs or symptoms of urinary tract obstruction without hydronephrosis or renal dysfunction	Signs or symptoms of urinary tract obstruction with hydronephrosis or renal dysfunction	Obstruction causing life-threatening consequences
**OCULAR/VISUAL**				
Uveitis	Asymptomatic but detectable on exam	Symptomatic anterior uveitis OR Medical intervention indicated	Posterior or pan-uveitis OR Operative intervention indicated	Disabling visual loss in affected eye(s)
Visual changes (from baseline)	Visual changes causing no or minimal interference with usual social & functional activities	Visual changes causing greater than minimal interference with usual social & functional activities	Visual changes causing inability to perform usual social & functional activities	Disabling visual loss in affected eye(s)
**ENDOCRINE/METABOLIC**				

CLINICAL				
**PARAMETER**	**GRADE 1**<br>**Mild**	**GRADE 2**<br>**Moderate**	**GRADE 3**<br>**Severe**	**GRADE 4**<br>**Very Severe**
Abnormal fat accumulation<br>(e.g., back of neck, breasts, abdomen)	Detectable by study participant (or by caregiver for young children and disabled adults)	Detectable on physical exam by health care provider	Disfiguring OR Obvious changes on casual visual inspection	NA
Diabetes mellitus	NA	New onset without need to initiate medication OR Modification of current medications to regain glucose control	New onset with initiation of medication indicated OR Diabetes uncontrolled despite treatment modification	Life-threatening consequences (e.g., ketoacidosis, hyperosmolar non-ketotic coma)
Gynecomastia	Detectable by study participant or caregiver (for young children and disabled adults)	Detectable on physical exam by health care provider	Disfiguring OR Obvious on casual visual inspection	NA
Hyperthyroidism	Asymptomatic	Symptomatic causing greater than minimal interference with usual social & functional activities OR Thyroid suppression therapy indicated	Symptoms causing inability to perform usual social & functional activities OR Uncontrolled despite treatment modification	Life-threatening consequences (e.g., thyroid storm)
Hypothyroidism	Asymptomatic	Symptomatic causing greater than minimal interference with usual social & functional activities OR Thyroid replacement therapy indicated	Symptoms causing inability to perform usual social & functional activities OR Uncontrolled despite treatment modification	Life-threatening consequences (e.g., myxedema coma)
Lipoatrophy<br>(e.g., fat loss from the face, extremities, buttocks)	Detectable by study participant (or by caregiver for young children and disabled adults)	Detectable on physical exam by health care provider	Disfiguring OR Obvious on casual visual inspection	NA

LABORATORY				
**HEMATOLOGY**	**Standard International Units are listed in italics**			
Absolute CD4+ count<br>**– Adult**	300 – 400/mm^3<br>300 – 400/µL	200 – 299/mm^3<br>200 – 299/µL	100 – 199/mm^3<br>100 – 199/µL	< 100/mm^3<br>< 100/µL
Absolute lymphocyte count<br>**– Adult**	600 – 650/mm^3<br>0.600 x 10^9 –<br>0.650 x 10^9/L	500 – 599/mm^3<br>0.500 x 10^9 –<br>0.599 x 10^9/L	350 – 499/mm^3<br>0.350 x 10^9 –<br>0.499 x 10^9/L	< 350/mm^3<br>< 0.350 x 10^9/L

CLINICAL				
**PARAMETER**	**GRADE 1**<br>**Mild**	**GRADE 2**<br>**Moderate**	**GRADE 3**<br>**Severe**	**GRADE 4**<br>**Very Severe**
Absolute neutrophil count (ANC)				
**Adult**	1,000 – 1,300/mm^3<br>1.000 x 10^9 –<br>1.300 x 10^9/L	750 – 999/mm^3<br>0.750 x 10^9 –<br>0.999 x 10^9/L	500 – 749/mm^3<br>0.500 x 10^9 –<br>0.749 x 10^9/L	< 500/mm^3<br>< 0.500 x 10^9/L
Fibrinogen, decreased	100 – 200 mg/dL<br>1.00 – 2.00 g/L<br>OR<br>0.75 – 0.99 x LLN	75 – 99 mg/dL<br>0.75 – 0.99 g/L<br>OR<br>0.50 – 0.74 x LLN	50 – 74 mg/dL<br>0.50 – 0.74 g/L<br>OR<br>0.25 – 0.49 x LLN	< 50 mg/dL<br>< 0.50 g/L<br>OR<br>< 0.25 x LLN<br>OR<br>Associated with gross bleeding
Hemoglobin (Hgb)				
**Adult**	10.0 – 10.9 g/dL<br>1.55 – 1.69 mmol/L<br>OR<br>Any decrease<br>2.5 – 3.4 g/dL<br>0.39 – 0.53 mmol/L	9.0 – 9.9 g/dL<br>1.40 – 1.54 mmol/L<br>OR<br>Any decrease<br>3.5 – 4.4 g/dL<br>0.54 – 0.68 mmol/L	7.0 – 8.9 g/dL<br>1.09 – 1.39 mmol/L<br>OR<br>Any decrease<br>≥ 4.5 g/dL<br>≥ 0.69 mmol/L	< 7.0 g/dL<br>< 1.09 mmol/L
International Normalized Ratio of prothrombin time (INR)	1.1 – 1.5 x ULN	1.6 – 2.0 x ULN	2.1 – 3.0 x ULN	> 3.0 x ULN
Methemoglobin	5.0 – 10.0%	10.1 – 15.0%	15.1 – 20.0%	> 20.0%
Prothrombin Time (PT)	1.1 – 1.25 x ULN	1.26 – 1.50 x ULN	1.51 – 3.00 x ULN	> 3.00 x ULN
Partial Thromboplastin Time (PTT)	1.1 – 1.66 x ULN	1.67 – 2.33 x ULN	2.34 – 3.00 x ULN	> 3.00 x ULN
Platelets, decreased	100,000 –<br>124,999/mm^3<br>100.000 x 10^9 –<br>124.999 x 10^9/L	50,000 –<br>99,999/mm^3<br>50.000 x 10^9 –<br>99.999 x 10^9/L	25,000 –<br>49,999/mm^3<br>25.000 x 10^9 –<br>49.999 x 10^9/L	< 25,000/mm^3<br>< 25.000 x 10^9/L
WBC, decreased	2,000 – 2,500/mm^3<br>2.000 x 10^9 –<br>2.500 x 10^9/L	1,500 – 1,999/mm^3<br>1.500 x 10^9 –<br>1.999 x 10^9/L	1,000 – 1,499/mm^3<br>1.000 x 10^9 –<br>1.499 x 10^9/L	< 1,000/mm^3<br>< 1.000 x 10^9/L
**CHEMISTRIES**	**Standard International Units are listed in italics**			
Acidosis	NA	pH < normal, but ≥ 7.3	pH < 7.3 without life-threatening consequences	pH < 7.3 with life-threatening consequences
Albumin, serum, low	3.0 g/dL – < LLN<br>30 g/L – < LLN	2.0 – 2.9 g/dL<br>20 – 29 g/L	< 2.0 g/dL<br>< 20 g/L	NA
Alkaline Phosphatase	1.25 – 2.5 x ULN[†]	2.6 – 5.0 x ULN[†]	5.1 – 10.0 x ULN[†]	> 10.0 x ULN[†]

CLINICAL				
**PARAMETER**	**GRADE 1**<br>**Mild**	**GRADE 2**<br>**Moderate**	**GRADE 3**<br>**Severe**	**GRADE 4**<br>**Very Severe**
Alkalosis	NA	pH > normal, but ≤ 7.5	pH > 7.5 without life-threatening consequences	pH > 7.5 with life-threatening consequences
ALT (SGPT)	1.25 – 2.5 x ULN	2.6 – 5.0 x ULN	5.1 – 10.0 x ULN	> 10.0 x ULN
AST (SGOT)	1.25 – 2.5 x ULN	2.6 – 5.0 x ULN	5.1 – 10.0 x ULN	> 10.0 x ULN
Bicarbonate, serum, low	16.0 mEq/L – < LLN<br>16.0 mmol/L – < LLN	11.0 – 15.9 mEq/L<br>11.0 – 15.9 mmol/L	8.0 – 10.9 mEq/L<br>8.0 – 10.9 mmol/L	< 8.0 mEq/L<br>< 8.0 mmol/L
Bilirubin (Total)				
**Adult**	1.1 – 1.5 x ULN	1.6 – 2.5 x ULN	2.6 – 5.0 x ULN	> 5.0 x ULN
Calcium, serum, high (corrected for albumin)				
**Adult**	10.6 – 11.5 mg/dL<br>2.65 – 2.88 mmol/L	11.6 – 12.5 mg/dL<br>2.89 – 3.13 mmol/L	12.6 – 13.5 mg/dL<br>3.14 – 3.38 mmol/L	> 13.5 mg/dL<br>> 3.38 mmol/L
Calcium, serum, low (corrected for albumin)				
**Adult**	7.8 – 8.4 mg/dL<br>1.95 – 2.10 mmol/L	7.0 – 7.7 mg/dL<br>1.75 – 1.94 mmol/L	6.1 – 6.9 mg/dL<br>1.53 – 1.74 mmol/L	< 6.1 mg/dL<br>< 1.53 mmol/L
Cardiac troponin I (cTnI)	NA	NA	NA	Levels consistent with myocardial infarction or unstable angina as defined by the manufacturer
Cardiac troponin T (cTnT)	NA	NA	NA	≥ 0.20 ng/mL<br>OR<br>Levels consistent with myocardial infarction or unstable angina as defined by the manufacturer
Cholesterol (fasting)				
**Adult**	200 – 239 mg/dL<br>5.18 – 6.19 mmol/L	240 – 300 mg/dL<br>6.20 – 7.77 mmol/L	> 300 mg/dL<br>> 7.77 mmol/L	NA
Creatine Kinase	3.0 – 5.9 x ULN†	6.0 – 9.9 x ULN†	10.0 – 19.9 x ULN†	≥ 20.0 x ULN†
Creatinine	1.1 – 1.3 x ULN†	1.4 – 1.8 x ULN†	1.9 – 3.4 x ULN†	≥ 3.5 x ULN†
Glucose, serum, high				
Nonfasting	116 – 160 mg/dL<br>6.44 – 8.88 mmol/L	161 – 250 mg/dL<br>8.89 – 13.88 mmol/L	251 – 500 mg/dL<br>13.89 – 27.75 mmol/L	> 500 mg/dL<br>> 27.75 mmol/L
Fasting	110 – 125 mg/dL<br>6.11 – 6.94 mmol/L	126 – 250 mg/dL<br>6.95 – 13.88 mmol/L	251 – 500 mg/dL<br>13.89 – 27.75 mmol/L	> 500 mg/dL<br>> 27.75 mmol/L
Glucose, serum, low				

CLINICAL				
**PARAMETER**	**GRADE 1 Mild**	**GRADE 2 Moderate**	**GRADE 3 Severe**	**GRADE 4 Very Severe**
**Adult**	55 – 64 mg/dL<br>3.05 – 3.55 mmol/L	40 – 54 mg/dL<br>2.22 – 3.06 mmol/L	30 – 39 mg/dL<br>1.67 – 2.23 mmol/L	< 30 mg/dL<br>< 1.67 mmol/L
Lactate	< 2.0 x ULN without acidosis	≥ 2.0 x ULN without acidosis	Increased lactate with pH < 7.3 without life-threatening consequences	Increased lactate with pH < 7.3 with life-threatening consequences
LDL cholesterol (fasting)				
**Adult**	130 – 159 mg/dL<br>3.37 – 4.12 mmol/L	160 – 190 mg/dL<br>4.13 – 4.90 mmol/L	≥ 190 mg/dL<br>≥ 4.91 mmol/L	NA
Lipase	1.1 – 1.5 x ULN	1.6 – 3.0 x ULN	3.1 – 5.0 x ULN	> 5.0 x ULN
Magnesium, serum, low	1.2 – 1.4 mEq/L<br>0.60 – 0.70 mmol/L	0.9 – 1.1 mEq/L<br>0.45 – 0.59 mmol/L	0.6 – 0.8 mEq/L<br>0.30 – 0.44 mmol/L	< 0.60 mEq/L<br>< 0.30 mmol/L
Pancreatic amylase	1.1 – 1.5 x ULN	1.6 – 2.0 x ULN	2.1 – 5.0 x ULN	> 5.0 x ULN
Phosphate, serum, low				
**Adult**	2.5 mg/dL – < LLN<br>0.81 mmol/L – < LLN	2.0 – 2.4 mg/dL<br>0.65 – 0.80 mmol/L	1.0 – 1.9 mg/dL<br>0.32 – 0.64 mmol/L	< 1.00 mg/dL<br>< 0.32 mmol/L
Potassium, serum, high	5.6 – 6.0 mEq/L<br>5.6 – 6.0 mmol/L	6.1 – 6.5 mEq/L<br>6.1 – 6.5 mmol/L	6.6 – 7.0 mEq/L<br>6.6 – 7.0 mmol/L	> 7.0 mEq/L<br>> 7.0 mmol/L
Potassium, serum, low	3.0 – 3.4 mEq/L<br>3.0 – 3.4 mmol/L	2.5 – 2.9 mEq/L<br>2.5 – 2.9 mmol/L	2.0 – 2.4 mEq/L<br>2.0 – 2.4 mmol/L	< 2.0 mEq/L<br>< 2.0 mmol/L
Sodium, serum, high	146 – 150 mEq/L<br>146 – 150 mmol/L	151 – 154 mEq/L<br>151 – 154 mmol/L	155 – 159 mEq/L<br>155 – 159 mmol/L	≥ 160 mEq/L<br>≥ 160 mmol/L
Sodium, serum, low	130 – 135 mEq/L<br>130 – 135 mmol/L	125 – 129 mEq/L<br>125 – 129 mmol/L	121 – 124 mEq/L<br>121 – 124 mmol/L	≤ 120 mEq/L<br>≤ 120 mmol/L
Triglycerides (fasting)	NA	500 – 750 mg/dL<br>5.65 – 8.48 mmol/L	751 – 1,200 mg/dL<br>8.49 – 13.56 mmol/L	> 1,200 mg/dL<br>> 13.56 mmol/L
Uric acid	7.5 – 10.0 mg/dL<br>0.45 – 0.59 mmol/L	10.1 – 12.0 mg/dL<br>0.60 – 0.71 mmol/L	12.1 – 15.0 mg/dL<br>0.72 – 0.89 mmol/L	> 15.0 mg/dL<br>> 0.89 mmol/L
**URINALYSIS**	**Standard International Units are listed in italics**			
Hematuria (microscopic)	6 – 10 RBC/HPF	> 10 RBC/HPF	Gross, with or without clots OR with RBC casts	Transfusion indicated

REFERENCES

[1] UNAIDS. Report on the Global AIDS Epidemic. Geneva: UNAIDS; 2008.

[2] Gillespie S, Kadiyala S, Greener R. Is poverty or wealth driving HIV transmission? AIDS 2007; 21 Suppl 7: S5-S16.

[3] McCutchan FE. Understanding the genetic diversity of HIV-1. AIDS 2000; 14(Suppl 3): S31-44.

[4] Osmanov S, Pattou C, Walker N, Schwardlander B, Esparza J. Estimated global distribution and regional spread of HIV-1 genetic subtypes in the year 2000. J Acquir Immune Defic Syndr 2002; 29: 184-90.

[5] Hemelaar J, Gouws E, Ghys PD, Osmanov S. Global and regional distribution of HIV-1 genetic subtypes and recombinants in 2004. AIDS 2006; 20: W13-23.

[6] Leitner T, Korber B, Daniels M, Calef C, Foley B. HIV-1 subtype and circulating recombinant form (CRF) reference sequences, 2005. 2005 [http://hiv-web.lanl.gov/content/hiv-db/REVIEWS/RefSeqs2005/RefSeqs05.html.

[7] Emini EA, Koff WC. AIDS/HIV. Developing an AIDS vaccine: need, uncertainty, hope. Science 2004; 5679: 1913-4.

[8] Berkley SF, Koff WC. Scientific and policy challenges to development of an AIDS vaccine. Lancet. 2007; 370: 94-101.

[9] Barouch DH, Nabel GJ. Adenovirus vector-based vaccines for human immunodeficiency virus type 1. Hum Gene Ther 2005; 16: 149-56.

[10] Casimiro DR, Chen L, Fu TM, Evans RK, Caulfield MJ, et al. Comparative immunogenicity in rhesus monkeys of DNA plasmid, recombinant vaccinia virus, and replication-defective adenovirus vectors expressing a human immunodeficiency virus type 1 gag gene. J Virol 2003; 77: 6305-13.

[11] Letvin NL, Huang Y, Chakrabarti BK, Xu L, Seaman MS, et al. Heterologous envelope immunogens contribute to AIDS vaccine protection in rhesus monkeys. J Virol 2004; 78: 7490-7.

[12] Santra S, Seaman MS, Xu L, Barouch DH, Lord CI, et al. Replication-defective adenovirus serotype 5 vectors elicit durable cellular and humoral immune responses in nonhuman primates. J Virol 2005; 79: 6516-22.

[13] Shiver JW, Emini EA. Recent advances in the development of HIV-1 vaccines using replication-incompetent adenovirus vectors. Annu Rev Med 2004; 55: 355-72.

[14] Shiver JW, Fu TM, Chen L, Casimiro DR, Davies ME, et al. Replication-incompetent adenoviral vaccine vector elicits effective anti-immunodeficiency-virus immunity. Nature 2002; 415: 331-5.

[15] Graham BS, Koup RA, Roederer M, Bailer RT, Enama ME, et al., Phase 1 safety and immunogenicity evaluation of a multiclade HIV-1 DNA candidate vaccine. J Infect Dis 2006; 194: 1650-60.

[16] Isaacs R. Evaluating the efficacy of the Merck adenovirus serotype 5-based trivalent MRKAD5GAG/POL/NEF (paper #42), in AIDS Vaccine 2005. 2005: Montreal, Canada.

[17] Shiver JW. Development of an HIV-1 vaccine based on replication-defective adenovirus, in Keystone Symposium on HIV Vaccine Development: Process and Prospects. 2004: Whistler, B.C., Canada.

[18] Catanzaro AT, Koup RA, Roederer M, Bailer RT, Enama ME, et al. Phase 1 safety and immunogenicity evaluation of a multiclade HIV-1 candidate vaccine delivered by a replication-defective recombinant adenovirus vector. J Infect Dis 2006; 194: 1638-49.

[19] Shiver JW, Emini EA. Recent advances in the development of HIV-1 vaccines using replication-incompetent adenovirus vectors. Annu Rev Med 2004; 55: 355-72.

[20] Jin X, Bauer DE, Tuttleton SE, Lewin S, Gettie A, et al. Dramatic rise in plasma viremia after CD8(+) T cell depletion in simian immunodeficiency virus-infected macaques. J Exp Med, 1999; 189: 991-8.

[21] Klein MR, van Baalen CA, Holwerda AM, Kerkhof Garde SR, Bende RJ, et al. Kinetics of Gag-specific cytotoxic T lymphocyte responses during the clinical course of HIV-1 infection: a longitudinal analysis of rapid progressors and long-term asymptomatics. J Exp Med 1995; 181: 1365-72.

[22] Moss RB, Webb E, Giermakowska WK, Jensen FC, Savary JR, et al. HIV-1-Specific CD4 helper function in persons with chronic HIV-1 infection on antiviral drug therapy as measured by ELISPOT after treatment with an inactivated, gp120-depleted HIV-1 in incomplete Freund's adjuvant. J Acquir Immune Defic Syndr 2000; 24: 264-9.

[23] Musey L, Hughes J, Schacker T, Shea T, Corey L, et al. Cytotoxic-T-cell responses, viral load, and disease progression in early human immunodeficiency virus type 1 infection. N Engl J Med 1997; 337: 1267-74.

[24] Nixon DF, Townsend AR, Elvin JG, Rizza CR, Gallwey J, et al. HIV-1 gag-specific cytotoxic T lymphocytes defined with recombinant vaccinia virus and synthetic peptides. Nature 1988; 336: 484-7.

[25] Schmitz JE, Kuroda MJ, Santra S, Sasseville VG, Simon MA, et al., Control of viremia in simian immunodeficiency virus infection by CD8+ lymphocytes. Science 1999; 283: 857-60.

[26] Yang O, Kalams SA, Rosenzweig M, Trocha A, Jones N, et al. Efficient lysis of human immunodeficiency virus type 1-infected cells by cytotoxic T lymphocytes. J Virol 1996; 70: 5799-806.

[27] Catanzaro AT, Roederer M, Koup RA, Bailer RT, Enama ME, Nason MC, *et al*. Phase I clinical evaluation of a six-plasmid multiclade HIV-1 DNA candidate vaccine. *Vaccine* 2007;25:4085-92.

[28] Harari A, Bart PA, Stöhr W, Tapia G, Garcia M, Medjitna-Rais E, *et al*. An HIV-1 clade C DNA prime, NYVAC boost vaccine regimen induces reliable, polyfunctional, and long-lasting T cell responses. *JEM* 2008;205:63-77.

[29] Vasan S, Schlesinger SJ, Chen Z, Hurley A, Lombardo A, Than S, *et al*. Phase 1 Safety and Immunogenicity Evaluation of ADMVA, a Multigenic, Modified Vaccinia Ankara-HIV-1 B'/C Candidate Vaccine. (*Submitted*)

[30] Leung L, Srivastava IK, Kan E, *et al*. Immunogenicity of HIV-1 Env and Gag in baboons using a DNA prime/protein boost regimen. *AIDS* 2004;18:991–1001.

[31] Sedegah M, Charoenvit Y, Aguiar J, *et al.* Effect on antibody and T-cell responses of mixing five GMP-produced DNA plasmids and administration with plasmid expressing GM-CSF. *Genes Immun* 2004;5:553–61.

[32] Bråve A, Ljungberg K, Boberg A, Rollman E, Engstörm G, Hinkula J, Wahren B. Reduced cellular immune responses following immunization with a multi-gene HIV-1 vaccine. *Vaccine* 2006; 24:4524-6.

[33] Toapanta FR, Craigo JK, Montelaro RC, Ross TM. Reduction of Anti-HIV-1 Gag Immune Responses During Co-Immunization: Immune Interference by the HIV-1 Envelope. *Current HIV Research* 2007;*5*:199-209.

[34] Hel Z, Tsai WP, Tryniszewska E, Nacsa J, Markham PD, Lewis MG, *et al*. Improved vaccine protection from simian AIDS by the addition of nonstructural simian immunodeficiency virus genes. *J Immunol* 2006;176:85-96.

[35] Priddy FH, Brown D, Kublin J, Monahan K, Wright DP, Lalezari J, *et al*. Safety and immunogenicity of a replication-incompetent adenovirus type 5 HIV-1 clade B gag/pol/nef vaccine in healthy adults. Clin Infect Dis 2008; 46: 1769-81.

[36] Cohen J. Did Merck's Failed HIV Vaccine Cause Harm. Science 2007; 318: 1048-9.

[37] Kostense S, Koudstaal W, Sprangers M, Weverling GJ, Penders G, et al. Adenovirus types 5 and 35 seroprevalence in AIDS risk groups supports type 35 as a vaccine vector. AIDS 2004; 18: 1213-6.

[38] Vogels R, Zuijdgeest D, van Rijnsoever R, Hartkoorn E, Damen I, et al. Replication-deficient human adenovirus type 35 vectors for gene transfer and vaccination: efficient human cell infection and bypass of preexisting adenovirus immunity. J Virol 2003; 77: 8263-71.

[39] Lemckert AA, Sumida SM, Holterman L, Vogels R, Truitt DM, Lynch DM, et al. Immunogenicity of heterologous prime-boost regimens involving recombinant adenovirus serotype 11 (Ad11) and Ad35 vaccine vectors in the presence of anti-ad5 immunity. J Virol 2005; 79: 9694-701.

[40] Waddington SN, McVey JH, Bhella D, Parker AL, Barker K, Atoda H, *et al.* Adenovirus serotype 5 hexon mediates liver gene transfer. Cell. 2008; 132(3): 397-409.

[41] Baum S. Adenoviruses. In: Mandell G, Bennett J, *et al*, eds. Principles and Practice of Infectious Diseases. New York: Churchill-Livingstone, 2000: 1624-30.

[42] Brown M, Rossier E, *et al*. Fatal adenovirus type 35 infection in newborns. Pediatr Infect Dis J 1991; 10: 955-6.

[43] Flomenberg PR, Chen M, *et al*. Molecular epidemiology of adenovirus type 35 infections in immunocompromised hosts. J Infect Dis 1987; 155: 1127-34.

[44] Hierholzer JC. Adenoviruses in the immunocompromised host. Clin Microbiol Rev 1992; 5: 262-74.

[45] Kim KS, Gohd RS. Fatal pneumonia caused by adenovirus type 35. Am J Dis Child 1981; 135: 473-5.

[46] Larsen RA, Jacobson JT, *et al*. Hospital-associated epidemic of pharyngitis and conjunctivitis caused by adenovirus (21/H21 + 35). J Infect Dis 1986; 154: 706-9.

[47] Pinto A, Beck R, *et al*. Fatal neonatal pneumonia caused by adenovirus type 35. Report of one case and review of the literature. Arch Pathol Lab Med 1992; 116: 95-9.

[48] Sanchez M P, Erdman DD, *et al*. Outbreak of adenovirus 35 pneumonia among adult residents and staff of a chronic care psychiatric facility. J Infect Dis 1997; 176: 760-3.

[49] Gray GC, McCarthy T, Lebeck MG, Schnurr DP, Russell KL, Kajon AE, *et al*. Genotype prevalence and risk factors for severe clinical adenovirus infection, United States 2004-2006. Clin Infect Dis. 2007; 45(9): 1120-31.

[50] Metzgar D, Osuna M, Kajon AE, Hawksworth AW, Irvine M, Russell KL. Abrupt emergence of diverse species B adenoviruses at US military recruit training centers. J Infect Dis. 2007; 196(10): 1465-73.

[51] Sivan AV, Lee T, Binn LN, Gaydos JC. Adenovirus-associated acute respiratory disease in healthy adolescents and adults: a literature review. Mil Med. 2007; 172(11): 1198-203.

[52] Shiver JW, Emini EA. Recent advances in the development of HIV-1 vaccines using replication-incompetent adenovirus vectors. Annu Rev Med 2004; 55:355-72.

[53] Barugahare B, Baker C, K'Aluoch O, Donovan R, Elrefaei M, Eggena M, *et al*. Human immunodeficiency virus-specific responses in adult Ugandans: patterns of cross-clade recognition. J Virol 2005; 79: 4132-9.

[54] Cao H, Kanki P, Sankale JL, Dieng-Sarr A, Mazzara GP, Kalams SA, *et al*. Cytotoxic T-lymphocyte cross-reactivity among different human immunodeficiency virus type 1 clades: implications for vaccine development. J Virol 1997; 71: 8615-23.

[55] McKinnon LR, Ball TB, Kimani J, Wachihi C, Matu L, Luo M, *et al*. Cross-clade CD8(+) T-cell responses with a preference for the predominant circulating clade. J Acquir Immune Defic Syndr 2005; 40: 245-9.

[56] Wilson SE, Pedersen SL, Kunich JC, Wilkins VL, Mann DL, Mazzara GP, *et al*. Cross-clade envelope glycoprotein 160-specific CD8+ cytotoxic T lymphocyte responses in early HIV type 1 clade B infection. AIDS Res Hum Retroviruses 1998; 14: 925-37.
